# *CIAO1* and *MMS19* deficiency: A lethal neurodegenerative phenotype caused by cytosolic Fe-S cluster protein assembly disorders

**DOI:** 10.1016/j.gim.2024.101104

**Published:** 2024-02-24

**Authors:** Clara D.M. van Karnebeek, Maja Tarailo-Graovac, René Leen, Rutger Meinsma, Solenne Correard, Judith Jansen-Meijer, Sergey V. Prykhozhij, Izabella A. Pena, Kevin Ban, Sarah Schock, Vishal Saxena, Mia L. Pras-Raves, Britt I. Drögemöller, Anita E. Grootemaat, Nicole N. van der Wel, Doreen Dobritzsch, Winfried Roseboom, Bauke V. Schomakers, Yorrick R.J. Jaspers, Lida Zoetekouw, Jeroen Roelofsen, Carlos R. Ferreira, Robin van der Lee, Colin J. Ross, Jakub Kochan, Rebecca L. McIntyre, Jan B. van Klinken, Michel van Weeghel, Gertjan Kramer, Bernhard Weschke, Philippe Labrune, Michèl A. Willemsen, Daria Riva, Barbara Garavaglia, John B. Moeschler, James J. Filiano, Marc Ekker, Jason N. Berman, David Dyment, Frédéric M. Vaz, Wyeth W. Wasserman, Riekelt H. Houtkooper, André B.P. van Kuilenburg

**Affiliations:** 1 Amsterdam UMC location University of Amsterdam, Departments of Pediatrics and Human Genetics, Emma Center for Personalized Medicine, Amsterdam, The Netherlands; 2 Emma Center for Personalized Medicine, Amsterdam UMC, Amsterdam, The Netherlands; 3 Departments of Medical Genetics and Pediatrics, Centre for Molecular Medicine and Therapeutics, Faculty of Pharmaceutical Science, BC Children’s Hospital Research Institute, University of British Columbia, Vancouver, BC, Canada; 4 United for Metabolic Diseases, Amsterdam, The Netherlands; 5 Amsterdam Gastroenterology Endocrinology Metabolism, Amsterdam, The Netherlands; 6 Departments of Medical Genetics and Biochemistry & Molecular Biology, Alberta Children’s Hospital Research Institute (ACHRI), Cumming School of Medicine, University of Calgary, Calgary, AB, Canada; 7 Amsterdam UMC location University of Amsterdam, Department of Clinical Chemistry, Laboratory Genetic Metabolic Diseases, Amsterdam, The Netherlands; 8 Core Facility Metabolomics, Amsterdam UMC, Amsterdam, The Netherlands; 9 Faculty of Medicine, CHEO Research Institute, University of Ottawa, Ottawa, ON, Canada; 10 The Picower Institute for Learning and Memory, Massachusetts Institute of Technology-MIT, Boston, MA; 11 Department of Biology, University of Ottawa, Ottawa, ON, Canada; 12 Rady Faculty of Health Sciences, Department of Biochemistry and Medical Genetics, Children’s Hospital Research Institute of Manitoba, University of Manitoba, Winnipeg, Manitoba, Canada; 13 Amsterdam UMC Location University of Amsterdam, Department of Medical Biology, Amsterdam, The Netherlands; 14 Uppsala University, Department of Chemistry, Biomedical Center, Uppsala, Sweden; 15 Swammerdam Institute for Life Sciences, University of Amsterdam, Laboratory for Mass Spectrometry of Biomolecules, Amsterdam, The Netherlands; 16 National Human Genome Research Institute, National Institutes of Health, Bethesda, MD; 17 Jagiellonian University, Faculty of Biochemistry, Biophysics and Biotechnology, Department of Cell Biochemistry, Kraków, Poland; 18 Department of Human Genetics, Leiden University Medical Center, Leiden, The Netherlands; 19 Department of Neuropediatrics, Charité University Medicine Berlin, Berlin, Germany; 20 APHP-Université Paris-Saclay, Hôpital Antoine Béclère, Centre de Référence Maladies Héréditaires du Métabolisme Hépatique, Service de Pédiatrie, Clamart, and Paris-Saclay University, and INSERM U 1195, Clamart, France; 21 Department of Pediatric Neurology and Donders Institute for Brain, Cognition and Behaviour, Radboud University Medical Center, Nijmegen, The Netherlands; 22 Neurogenetic Syndromes and Autism Spectrum Disorders Unit, Fondazione IRCCS Istituto Neurologico “Carlo Besta,” Milan, Italy; 23 Medical Genetics and Neurogenetics Unit, Fondazione IRCCS Istituto Neurologico “Carlo Besta,” Milan, Italy; 24 Geisel School of Medicine, Dartmouth College and Departments of Pediatrics, Children’s Hospital at Dartmouth, Lebanon, NH

**Keywords:** CIAO1 and MMS19, Cofactor, Infection, Iron-sulfur clusters, Neurodegeneration

## Abstract

**Purpose:**

The functionality of many cellular proteins depends on cofactors; yet, they have only been implicated in a minority of Mendelian diseases. Here, we describe the first 2 inherited disorders of the cytosolic iron-sulfur protein assembly system.

**Methods:**

Genetic testing via genome sequencing was applied to identify the underlying disease cause in 3 patients with microcephaly, congenital brain malformations, progressive developmental and neurologic impairments, recurrent infections, and a fatal outcome. Studies in patient-derived skin fibroblasts and zebrafish models were performed to investigate the biochemical and cellular consequences.

**Results:**

Metabolic analysis showed elevated uracil and thymine levels in body fluids but no pathogenic variants in *DPYD*, encoding dihydropyrimidine dehydrogenase. Genome sequencing identified compound heterozygosity in 2 patients for missense variants in *CIAO1*, encoding cytosolic iron-sulfur assembly component 1, and homozygosity for an in-frame 3-nucleotide deletion in *MMS19,* encoding the MMS19 homolog, cytosolic iron-sulfur assembly component, in the third patient. Profound alterations in the proteome, metabolome, and lipidome were observed in patient-derived fibroblasts. We confirmed the detrimental effect of deficiencies in *CIAO1* and *MMS19* in zebrafish models.

**Conclusion:**

A general failure of cytosolic and nuclear iron-sulfur protein maturation caused pleiotropic effects. The critical function of the cytosolic iron-sulfur protein assembly machinery for antiviral host defense may well explain the recurrent severe infections occurring in our patients.

## Introduction

Cofactors are non-protein chemical compounds or metallic ions that are required for an enzyme’s role as a catalyst, and as such, they play a crucial role in the functionality of numerous cellular proteins. Although most cofactors can be either imported or synthesized, inorganic iron-sulfur clusters (ISCs) solely rely on intracellular synthesis in almost all known organisms.^1,2^ The ISCs represent one of the most ancient, versatile, and ubiquitous classes of metal cofactors participating in fundamental biological processes, such as mitochondrial respiration, numerous anabolic and catabolic reactions, protein translation, tRNA base modification, mitosis, the complex process of genome maintenance, and antiviral defense.^1,3,4^ In eukaryotes, approximately 50 to 70 unique iron-sulfur proteins have been identified to be located in mitochondria, plastids, cytosol, nucleus, and endoplasmic reticulum.^1,3^ The biogenesis of cytosolic and nuclear iron-sulfur proteins requires a close cooperation between the mitochondrial ISC assembly machinery and the cytosolic ISC machinery (CIA).^5–7^ In addition, there is evidence suggesting a de novo synthesis of ISC in the cytosolic and nuclear compartments.^8,9^

To date, only inherited defects of the mitochondrial ISC machinery have been reported, and these patients present with metabolic, neurological, and hematological phenotypes, often leading to early death during childhood.^1,9–12^ The general consensus is that complete loss-of-function variants in genes responsible for the mitochondrial ISC machinery lead to a severe phenotype and neonatal death, whereas partial loss-of-function variants are associated with a less severe clinical presentation that is tissue-specific.^10,13^ Although defects in the ISC machinery are associated with a broad range of phenotypic outcomes and low clinical predictability, the resulting biochemical consequences can be readily explained or predicted. In this article, we demonstrate the pivotal role of deep clinical and biochemical phenotyping in guiding genome sequencing to solve the unknown in rare phenotypes, ie, the identification of the first cytosolic iron-sulfur cluster protein assembly disorders as a source of apparent missing heritability in Mendelian diseases.

## Materials and Methods

### Clinical data and specimen collection

The study cohort consists of 3 affected patients who were referred to the Amsterdam UMC, in The Netherlands for diagnostic testing for dihydropyrimidine dehydrogenase (DPD) deficiency.

### Sequencing and genomic analysis

Genomic DNA was isolated from fibroblasts of the 3 probands and the coding exons, and flanking intronic sequences of *DPYD* were analyzed for sequence variants and copy-number changes, as described before.^14^ Singleton genome sequencing analysis was performed for each proband and analyzed using an in-house semi-automated approach described previously.^15^ Sanger sequencing was used to confirm the missense and deletion variants. Details regarding this analysis are provided in the Supplemental Methods. Variants were classified using the ACMG/AMP criteria, the details of the score attributed to each variant are available in Supplemental Tables S1.^16^

### Biochemical analysis

Concentrations of uracil and thymine in body fluids were determined using reversed-phase high-performance liquid chromatography coupled with electrospray tandem mass spectrometry. The activity of dihydropyrimidine dehydrogenase, encoded by the *DPYD* gene, was determined in a reaction mixture containing the substrate [4-^14^C]-thymine. Separation of radiolabeled thymine from the radiolabeled product dihydrothymine was performed by reversed-phase high-performance liquid chromatography with online detection of the radioactivity.^17^

### Computational and functional analysis

Functional analysis of the *CIAO1* and *MMS19* mutants was performed by in silico protein crystal structure analysis, zebrafish modeling, and viral transfection of patients’ fibroblasts with wild-type *CIAO1* and *MMS19* (Supplemental Tables S2–S3). The proteome, metabolome, and lipidome were analyzed using an ultra-high-pressure liquid chromatography system coupled with a high-resolution mass spectrometer. Technical details of all methods used are described in the Supplemental Appendix.

## Results

### Clinical presentation

**Figure 1:**
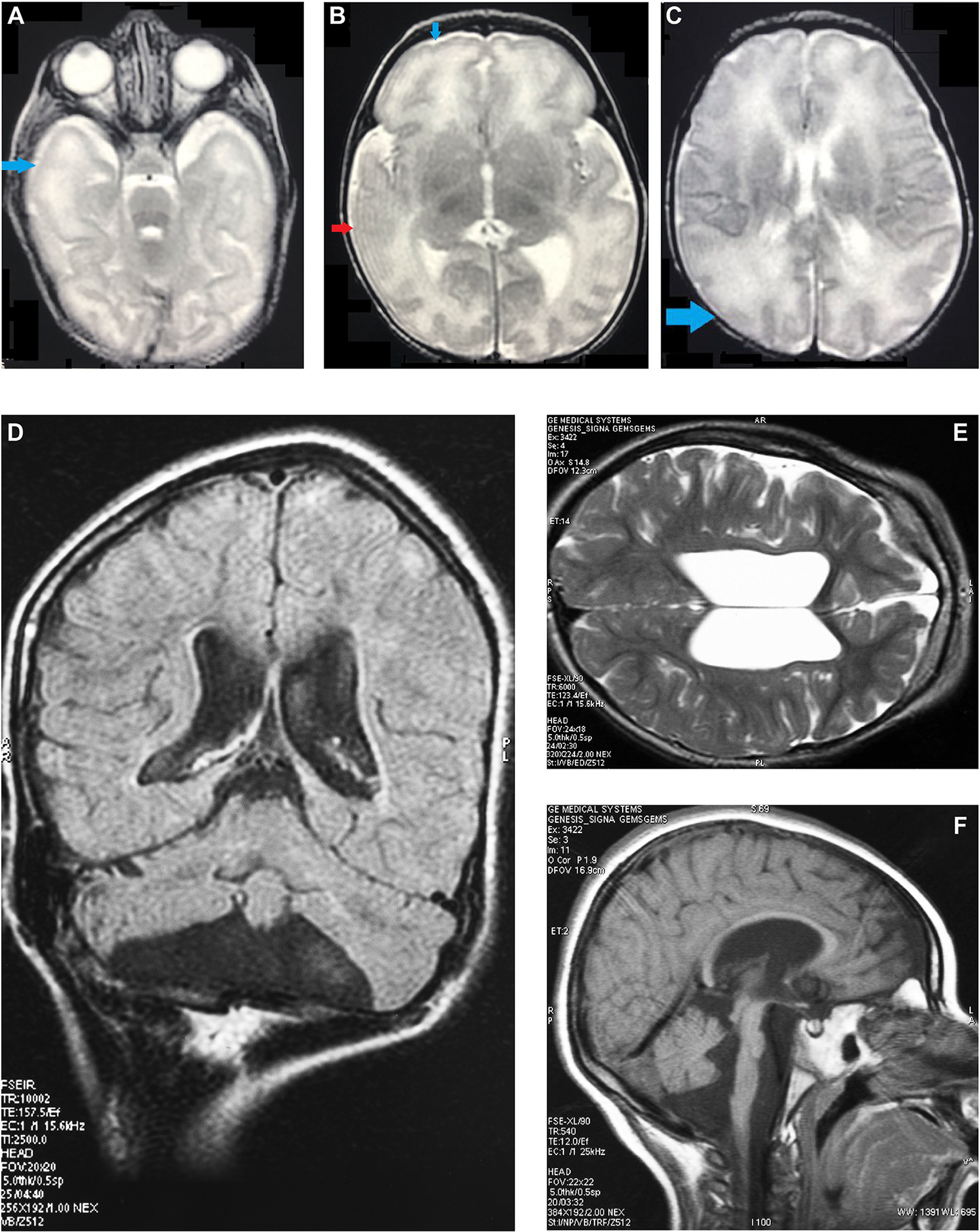
Magnetic Resonance Images (MRI) of patient 1 and 3. T2-weighted images at the age of 5 days of patient 1 showed regions in both hemispheres with a simplified gyral pattern, indicated by blue arrows in (A), (B), and (C). The red arrow in (B) marks a possible abnormal lateral right temporal lobe, but the actual anatomy is obscured by a Gibbs artifact. MRI of patient 3 at the age of 4 years ([D] (FLAIR), [E] (T2 weighted), and [F] (T1 weighted) showed enlarged lateral and third ventricles, decreased volume of cerebral white matter and incomplete myelination, and pontocerebellar atrophy.

Three unrelated patients with unremarkable family history were born at term. Patient 1 (female) was born full term after a pregnancy complicated by intra-uterine growth retardation and a small placenta, with a low birth weight, short stature, microcephaly, borderline hypertonia, and adducted thumbs. Apgar scores were normal, but soon after birth, she experienced severe neonatal desaturations and bradycardia, without overt convulsions. Electroencephalogram revealed independent, multifocal sharp waves in left and right temporal and right frontal regions. Following treatment with phenobarbital, her clinical status and electroencephalogram improved. Brain magnetic resonance images (MRI) at age 5 days showed regions with fewer gyri and sulci than expected for age (Figure 1) with unremarkable spectroscopy; skeletal gross morphology was normal by the combination of X-ray survey and clinical examination; abdominal ultrasound was normal. Microcephaly and poor growth persisted. She died at 18 months of respiratory failure from bronchiolitis complicated by apparent bacterial and fungal superinfection, not responsive to bronchial lavage, antibiotics, and oscillator ventilation.

Patient 2 (male) experienced failure to thrive with diarrhea during infancy, a hyperkinetic movement disorder, autism spectrum disorder, and behavioral problems. Cerebral MRI at age 6 years was unremarkable. During his late teens, he deteriorated with muscle weakness and wheelchair dependency, recurrent bouts of pneumonia with respiratory insufficiency, ultimately requiring intubation. He died at 19 years of age after multi-organ failure with carnificating pneumonia, septic cardiomyopathy, and intracranial hemorrhages. Immune deficiency was ruled out.

Patient 3 (female) was born after an uncomplicated pregnancy with normal growth parameters, torticollis and hypertonia of the hip adductor muscles, and facial dysmorphisms. She developed microcephaly, bilateral cataracts, failure to thrive, progressive spastic tetraparesis, scoliosis, myoclonic epilepsy, and precocious puberty. Cerebral MRI at age 4 years showed pontocerebellar atrophy and white matter abnormalities (Figure 1). Recurrent respiratory tract infections ultimately led to her demise at age 13 years. Additional clinical details of all 3 patients are provided in the Supplemental Appendix.

### Metabolic and enzyme analyses

**Figure 2:**
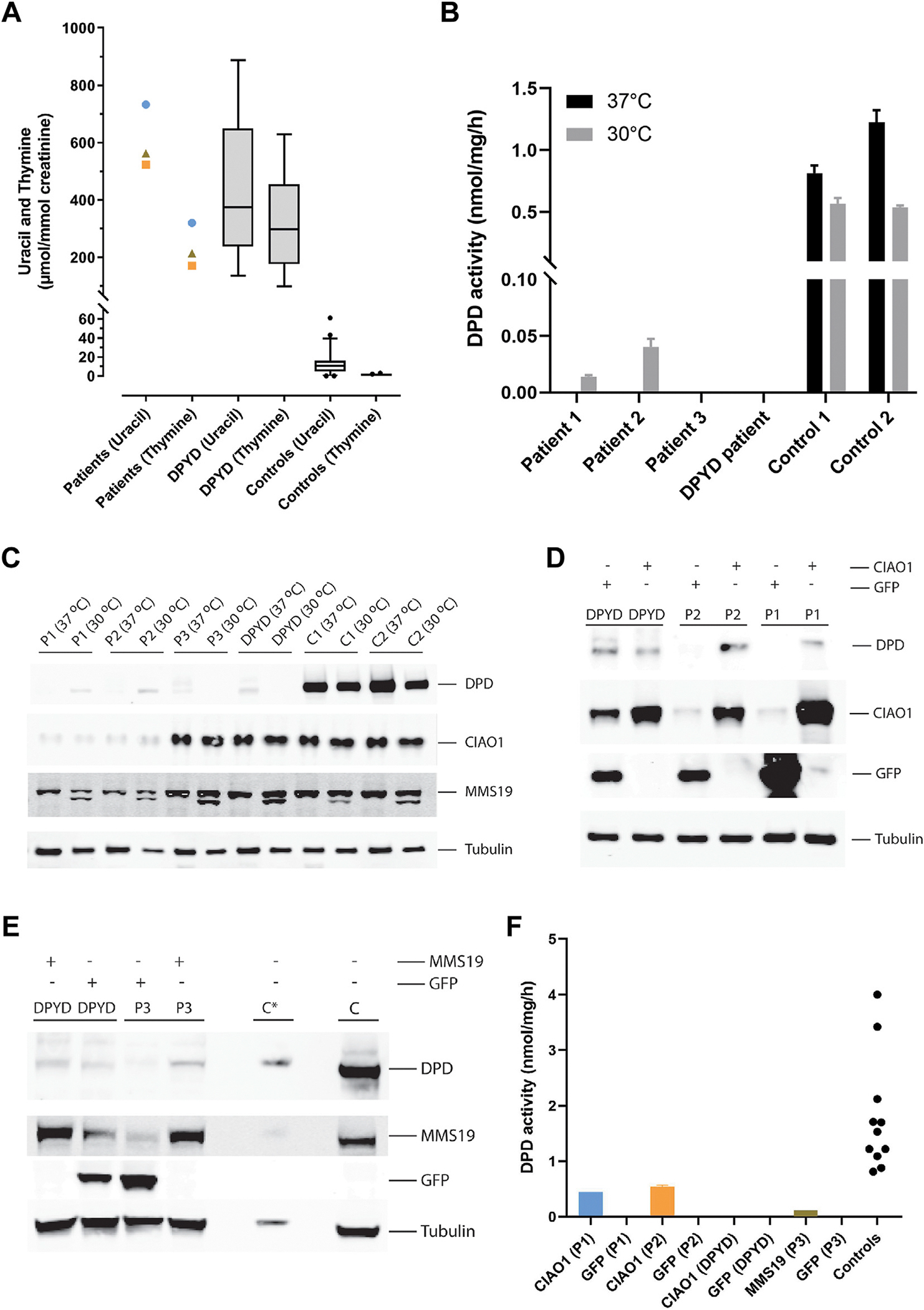
Biochemical characterization of CIAO1 and MMS19 deficiency. A. The urinary uracil and thymine concentrations from all 3 study patients (patient 1, blue circle; patient 2, orange square; and patient 3, brown triangle) and boxplots of the urinary uracil and thymine concentrations in *DPYD*-deficient patients and controls. B. The results of the DPD activity analysis in fibroblasts cultured at 37°C and 30°C. C. An immunoblot analysis of DPD, CIAO1, and MMS19 in fibroblasts from the 3 study patients, a *DPYD*-deficient patient and 2 controls, cultured at 37°C and 30°C. D. The immunoblot analysis of CIAO1 and DPD in fibroblasts of patients 1, 2, and a *DPYD*-deficient patient, stable-transfected with wild-type CIAO1. E. The immunoblot analysis of MMS19 and DPD in fibroblasts of patient 3 and a *DPYD*-deficient patient, stable-transfected with wild-type MMS19. Control fibroblasts C (10 μg protein) and C* (1 μg protein). F. The reappearance of the DPD activity in fibroblasts of the patients 1 and 2 stably transfected with wild-type *CIAO1* and in fibroblasts of patient 3 stably transfected with wild-type *MMS19*.

Metabolic testing revealed strongly elevated levels of uracil and thymine in urine (Figure 2A), plasma and cerebrospinal fluid (Supplemental Figure S1) in all 3 patients. Subsequent analysis of the DPD activity in fibroblasts of the 3 patients and a known patient with DPD deficiency, due to homozygosity for the NM_000110.4:c.1905+1G>A variant in *DPYD*, showed no DPD activity in fibroblasts cultured at 37°C. However, residual DPD activity and DPD protein could be detected in fibroblasts of patients 1 and 2 when cultured at 30°C (Figure 2), a temperature favoring the folding and maturation of (mutant) proteins.^18^ Thus, these investigations confirmed DPD deficiency in all 3 patients.

### *DPYD* and genome sequencing

Sequence and copy-number analysis of all 23 coding exons and flanking intronic regions of *DPYD* in the 3 patients did not reveal the presence of any variants of significance nor deletions or amplifications in *DPYD*. Quantitative PCR analysis in patients’ fibroblasts showed that *DPYD* mRNA was readily expressed (Supplemental Figure S2). Furthermore, cDNA analysis confirmed presence of a normal coding sequence for *DPYD*. Fusion of fibroblasts of the 3 patients with a known *DPYD* deficiency fibroblast line, due to homozygosity for the deleterious NM_000110.4:c.299_302del variant, resulted in the reappearance of detectable albeit low DPD activity (Supplemental Figure S3). These results demonstrate that the apparent DPD deficiency in the 3 patients may be caused by (an)other gene(s) and that *DPYD* itself is fully functional.

Genome-wide sequencing (details in Supplemental Appendix) showed that patient 1 was heterozygous for the variants NM_004804.2:c.193C>T p.(Arg65Trp), hereafter referred to as R65W, and NM_004804.2:c.577C>T p.(His193Tyr), hereafter referred to as H193Y, in *CIAO1*, encoding cytosolic iron-sulfur assembly component 1 (Supplemental Table S1). Patient 2 harbored the R65W variant in *CIAO1*, in addition to the NM_004804.2:c.552G>C p.(Trp184Cys) variant in *CIAO1* (Supplemental Table S1), hereafter referred to as W184C. In patient 3, homozygosity for a 3-nucleotide deletion NM_001289405.1:c.637_639del p.(Glu213del) in *MMS19*, hereafter referred to as E213del, encoding the MMS19 homolog, cytosolic iron-sulfur assembly component, was observed (Supplemental Table S1). The 3 missense variants in *CIAO1* were predicted as damaging by all tested in silico metrics (SIFT; Sorting Intolerant From Tolerant, PolyPhen-2, and CADD; Combined Annotation Dependent Depletion). For the E213del variant in *MMS19* no in silico metrics were available. The variant R65W (dbSNP: rs11544859) in *CIAO1* has an allele frequency of 1.01 × 10^−3^, with no individual homozygous for the alternate allele, in the Genome Aggregation Database (gnomADv2). The H193Y (rs902409423) and W184C variants in *CIAO1* and the E213del in *MMS19* were absent from this database.

The variants detected in *CIAO1* in both patients were located in trans (Supplemental Appendix). Parents of the patient 3, who was homozygous for the E213del variant in *MMS19*, were confirmed to be heterozygotes.

### *CIAO1* and *MMS19* variants

*CIAO1* and *MMS19* encode proteins indispensable for the maturation and activity of the homodimeric DPD enzyme, which contains 2 FAD, 2 FMN and 8 [4Fe-4S] clusters.^19–21^ Immunoblot analysis showed severely reduced protein levels of CIAO1 in fibroblasts of patients 1 and 2 compared with fibroblasts of a patient with *DPYD* deficiency and controls (Figure 2C). The expression of the mutant MMS19 protein in fibroblasts of patient 3 was comparable with that of the wild-type MMS19 protein in control fibroblasts. In addition, slightly reduced levels of MMS19 were observed in patients 1 and 2. To confirm the pathogenicity of the identified variants in *CIAO1* and *MMS19*, fibroblasts of the patients were transduced with wild-type *CIAO1* or *MMS19.* Stable-transfected fibroblasts of patients 1 and 2 with cDNA coding for wild-type *CIAO1* resulted in a profound expression of the wild-type CIAO1 protein and reappearance of the mature DPD protein (Figure 2D) and DPD activity (Figure 2F). For fibroblasts of patient 3, stable-transfection with cDNA encoding wild-type *MMS19* resulted in expression of the wild-type MMS19 protein, the mature DPD protein (Figure 2E), and a low but detectable DPD enzyme activity (Figure 2F). Introduction of either wild-type *CIAO1* or *MMS19* in fibroblasts of a patient with *DPYD* deficiency did not result in increased DPD protein expression or DPD activity (Figure 2).

### CIAO1 and MMS19 dysfunction

To investigate whether the severely reduced CIAO1 protein levels in fibroblasts of patient 1 and 2 might be caused by a decreased stability of the mutant CIAO1 proteins, we investigated the relative stability of recombinantly expressed mutant CIAO1 protein carrying the R65W, W184C, or H193Y variant. No significant difference in stability was observed for CIAO1 carrying the R65W variant, whereas the CIAO1 proteins carrying the W184C or H193Y variant proved to be less stable than that observed for wild-type CIAO1 (Supplemental Figure S4). These findings are in line with an analysis of the available crystal structure of CIAO1, which showed that the R65W variant is likely to abrogate interaction with (putative) proteins that bind at this surface and that the W184C and H193Y variants destabilize the structure and thus have direct or indirect effects on CIAO1’s ability to interact with other proteins (Supplemental Figure S5). The stability of the recombinantly expressed mutant MMS19 carrying the E213del variant was comparable to that observed for the wild-type MMS19 protein (Supplemental Figure S4). This observation is in agreement with the MMS19 structural model, which showed that the E213del variant should not affect the folding and stability of MMS19 (Supplemental Figure S6). However, the E213del variant most likely affects the interaction with client proteins and homodimerization of the core cytosolic Fe-S protein assembly (CIA) system (Supplemental Figure S6).

### Cytosolic Fe-S protein assembly system

CIAO1 and MMS19 are 2 components of the CIA system (Supplemental Figure S7). The CIA complex binds to and facilitates the assembly of most cytosolic-nuclear ISC-containing proteins.^19,20^ Co-immune precipitation of transiently expressed wild-type and mutant MMS19, carrying a C-terminal V5 tag in HEK293T cells, showed that both wild-type and mutant MMS19 were able to bind CIAO1 (Supplemental Figure S8). However, native gel electrophoresis showed that the mutant MMS19 protein was associated with a lower molecular weight protein complex when compared with that observed for the wild-type MMS19 protein. The recombinant expression of wild-type CIAO1 and mutant R65W CIAO1 resulted in the formation of a large protein complex with a comparable molecular weight. In contrast, only a very low amount of a normalsized and smaller-sized protein complex was observed for the recombinant CIAO1 carrying the W184C or H193Y variants (Supplemental Figure S9).

### Protein homeostasis

**Figure 3:**
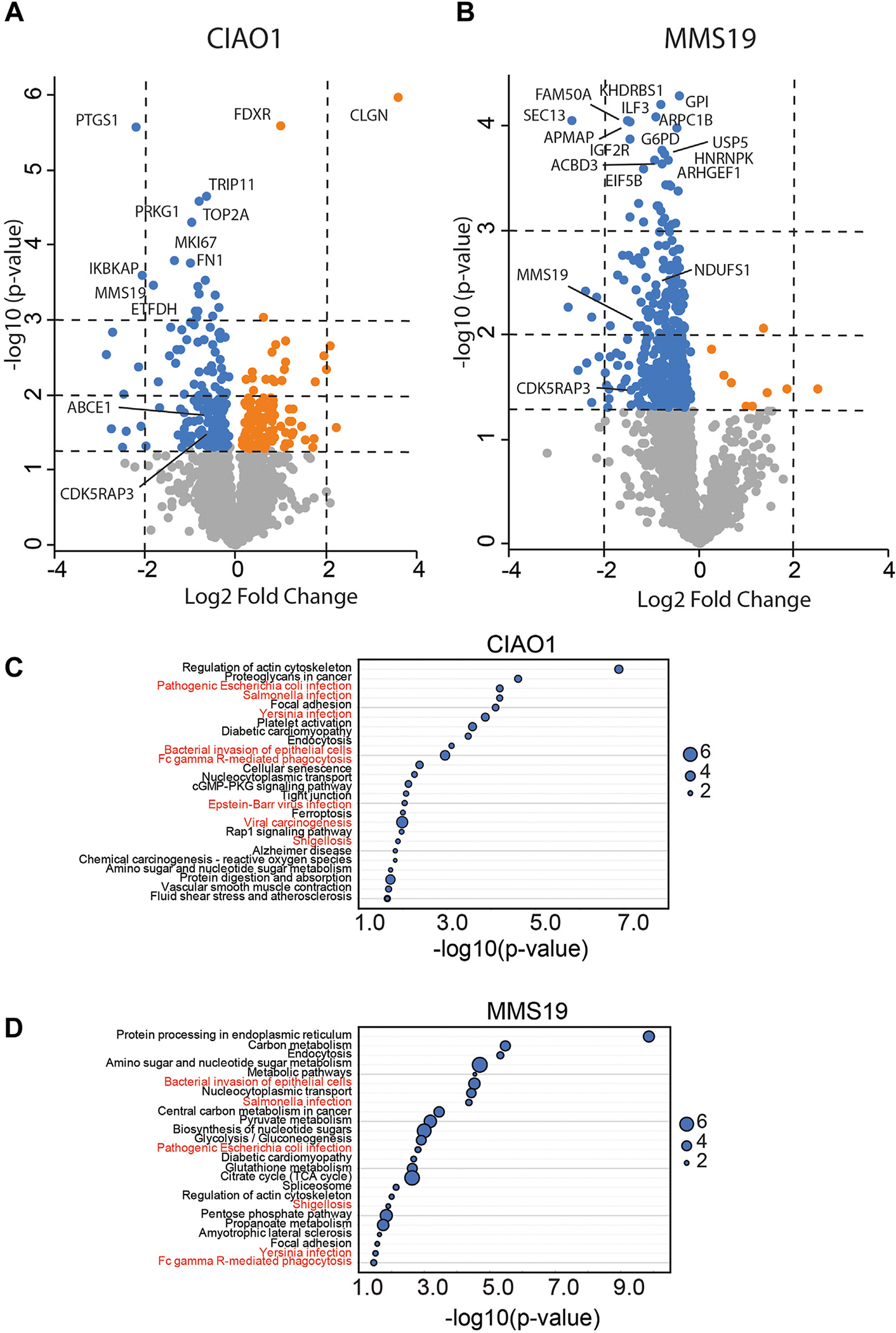
Volcano plot depicting proteomics data in CIAO1 and MMS19 deficient fibroblasts. (A) CIAO1-deficient fibroblasts and (B) MMS19-deficient fibroblasts. The blue and orange symbols represent significantly decreased and increased protein abundance, respectively. The most significantly changed proteins and known iron-sulfur proteins are labeled. The 3 horizontal dotted lines indicate significance cutoff *P* values of .05, .01, and .001, respectively. The 2 vertical dotted lines indicate log2(fold change) −2 and 2, respectively. C and D. Enrichment analysis showing the affected pathways in CIAO1- (C) and MMS19 (D)-deficient fibroblasts. The enrichment ratio reflects the number of significantly altered proteins versus the expected number of altered proteins for a given enrichment set. Pathways associated with bacterial and viral infections are depicted in red.

In fibroblasts of the 3 patients, no differences were observed in the protein expression, apart from DPD, of phosphoribosylpyrophosphate amidotransferase (PPAT), xeroderma pigmentosum protein D (XPD), and DNA Polymerase Delta 1 (POLD1), which are known client proteins of CIAO1 and MMS19^19,20,22^ (Supplemental Figure S10). The potential impact of CIAO1 and MMS19 dysfunction on protein homeostasis was investigated further using a proteomics approach. The analysis of 2347 proteins showed that the abundance of 283 proteins in CIAO1-deficient fibroblasts was significantly altered compared with control fibroblasts (Figure 3). Among these proteins, only a few known iron-sulfur proteins, such as ETFDH, ABCE1, and CDK5RAP3 were differentially regulated in the CIAO1-deficient fibroblasts. For MMS19-deficient fibroblasts, the abundance of 401 proteins (Figure 3) was significantly altered compared with control fibroblasts, including the iron-sulfur proteins NDUFS1 and CDK5RAP3. No differences were observed in the protein expression, apart from DPD, in known client proteins of CIAO1 and MMS19 (Supplemental Figure S10).

To identify pathways affected by CIAO1 and MMS19 deficiencies, an enrichment analysis was performed using predefined sets covering various pathways (Figure 3). Pathways associated with bacterial and viral infections were profoundly affected, as well as several metabolic pathways.

### Metabolic footprint

**Figure 4:**
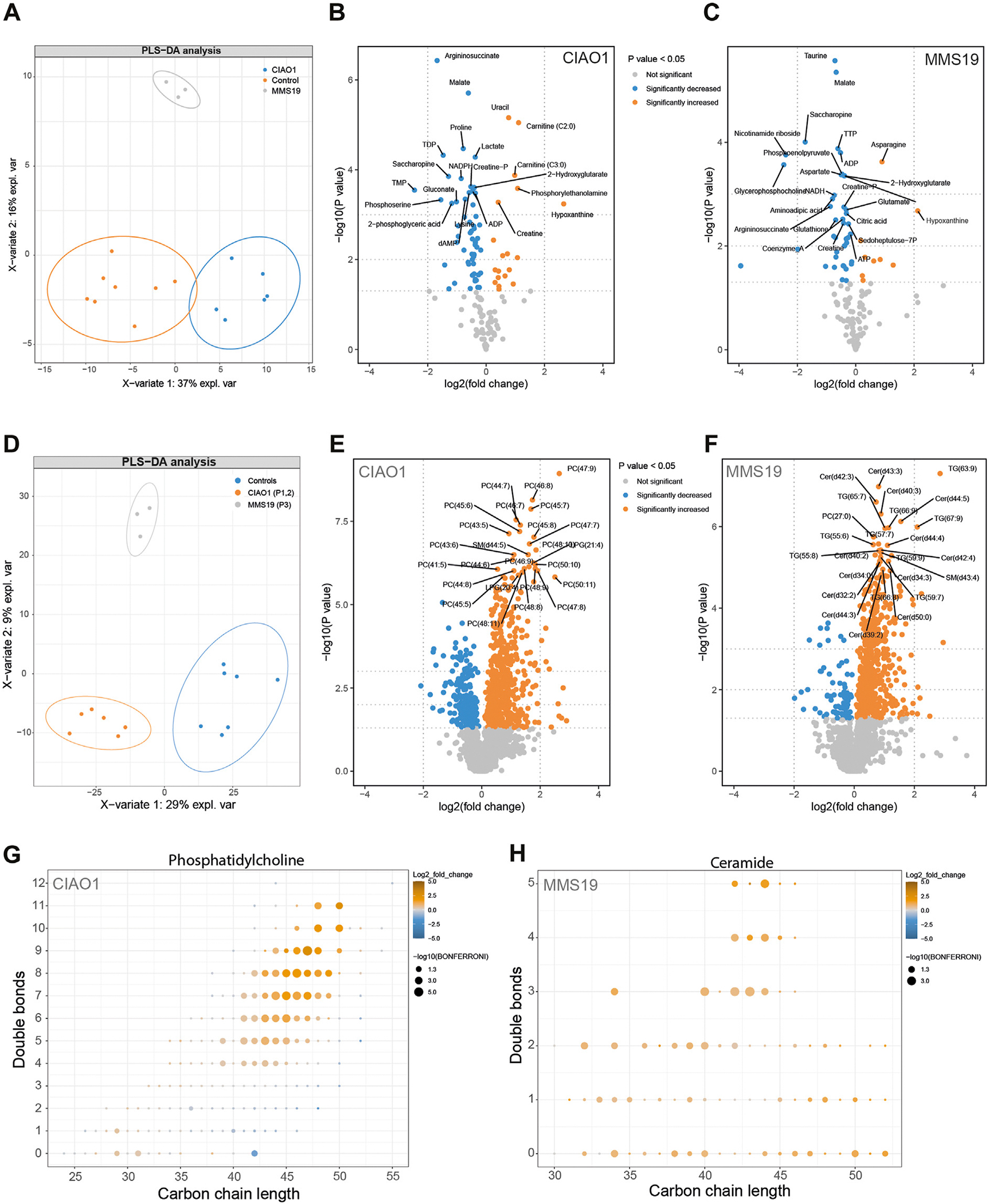
Altered metabolome and lipidome in CIAO1- and MMS19-deficient fibroblasts. A. PLS-DA analysis shows a clear distinction between the metabolome of CIAO1, MMS19, and control fibroblasts. B and C. Volcano plot depicting the metabolomics data in CIAO1 (B) and MMS19 deficient fibroblasts (C). Significance cutoff is shown in the legend of the volcano plot. The 25 most changed metabolites based on *P* value are labeled. The 3 horizontal dotted lines indicate *P* value .05, .01, and .001, respectively. The 2 vertical dotted lines indicate log2(fold change) −2 and 2, respectively. D. PLS-DA analysis shows a clear distinction between the lipidome of CIAO1, MMS19, and control fibroblasts. E and F. Volcano plot depicting the lipidomics data in CIAO1 (E) and MMS19 deficient fibroblasts (F). Significance cutoff is shown in the legend of the volcano plot. The 50 most changed metabolites based on *P* value are labeled. The 3 horizontal dotted lines indicate *P* value .05, .01, and .001, respectively. The 2 vertical dotted lines indicate log2(fold change) −2 and 2, respectively. G and H. The carbon-chain length of phosphatidylcholine and ceramide plotted versus the total number of double bonds in those chains. Changes in the phosphatidylcholine in CIAO1-deficient fibroblasts (G) and ceramide composition in MMS19-deficient fibroblasts (H) versus controls show significant increases of phospholipids with long carbon-chain length and double bonds.

Metabolomics analysis focusing on polar metabolites showed that the metabolome of fibroblasts of CIAO1- and MMS19-deficient fibroblasts was markedly different from that observed in control fibroblasts (Figure 4). In both CIAO1- and MMS19-deficient fibroblasts, the levels of uracil and thymine were increased compared with control fibroblasts, which is in line with the presence of a DPD deficiency in the patients’ cell lines. Network analysis to visualize the quantified metabolites and their biochemical relation in a holistic fashion (Supplemental Figure S11) showed a large variety of affected metabolic pathways, including amino acid metabolism, nucleotide metabolism, citric acid cycle, and methionine-cysteine pathway. In situ analysis of CIAO1 and MMS19 deficiency, using stableisotope-labeled metabolites, confirmed altered fluxes through the citric acid cycle and methionine-cysteine pathway (Supplemental Figures S12–S14).

Lipidomics analysis of 2047 lipid species showed that the lipidome of CIAO1- and MMS19-deficient fibroblasts was significantly different from control fibroblasts (Figure 4). The concentration of 779 lipid species (38%) in CIAO1 deficient fibroblasts (Figure 4) and 660 lipid species (32%) in MMS19 deficient fibroblasts (Figure 4) were significantly altered compared with control fibroblasts. A strong increase in phosphatidylcholines and ceramide, containing polyunsaturated long-chain fatty acids, were observed in CIAO1 and MMS19, respectively (Figure 4). A similar phenomenon was also observed for other lipid species such as triacylglycerols containing polyunsaturated long-chain fatty acids (Supplemental Figure S15).

### Cellular phenotype

The presence of a CIAO1 or MMS19 deficiency was associated with a reduced proliferation of fibroblasts of all 3 patients compared with control fibroblasts (Supplemental Figure S16), as well as an increased radio-sensitivity of MMS19 deficient fibroblasts (Supplemental Figure S17). Despite the profound effect of CIAO1 and MMS19 deficiencies on protein homeostasis and metabolism, both immunofluorescence and electron microscopy showed no evident differences for the cellular compartments and organelles (Supplemental Figure S18–S22).

### Zebrafish models and Ciao1 and Mms19 deficiency

**Figure 5:**
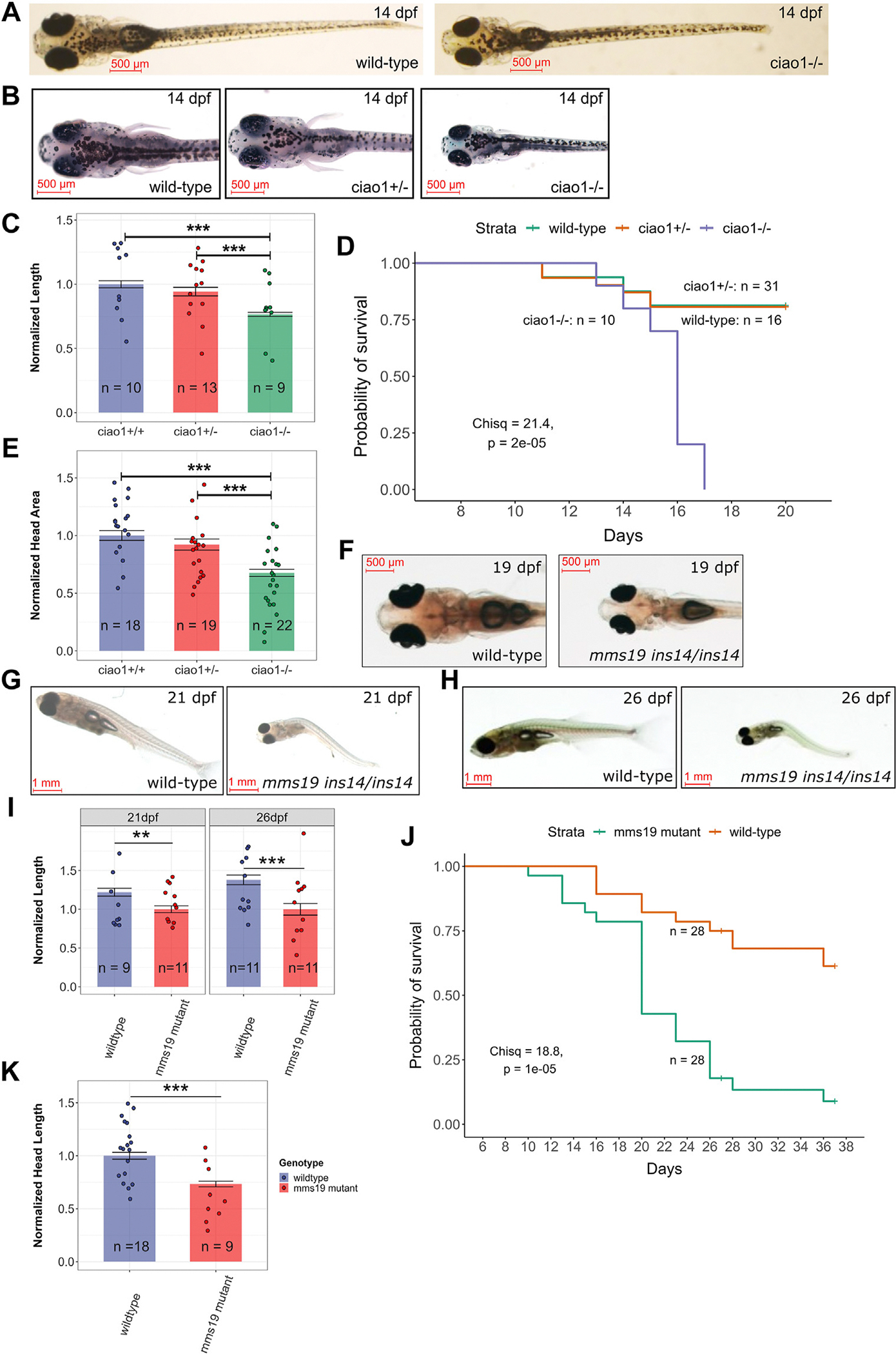
*ciao1*−/− and *mms19* −/− zebrafish phenotypes. A. Whole-body images of representative wild-type and *ciao1*−/− larvae at 14 dpf. B. Head images of representative wild-type, *ciao1*+/−, and *ciao1*−/− larvae at 14 dpf. C. Graph of larval lengths of zebrafish with different *ciao1* genotypes at 14 dpf, wild type, *n* = 10; *ciao1*+/−, *n* = 13; *ciao1*−/−, *n* = 9. D. Kaplan-Meier survival plot showing early death in the homozygous mutants *ciao1*−/−, *n* = 10; *ciao1*+/−, *n* = 31; wild type, *n* = 16. E. Graph of head areas of zebrafish with different *ciao1* genotypes at 14 dpf, wild type, *n* = 18; *ciao1*+/−, *n* = 19; *ciao1*−/−, *n* = 22. F. Representative head images of wild-type and *mms19*^*ins14/ins14*^ mutants at 19 dpf. Overall morphology of the wild-type and *mms19*^*ins14/ins14*^ mutants at 21 dpf (G) and 26 dpf (H) demonstrates serious abnormalities of the mutant adults. I. Normalized length (by mutant mean) quantification of wild-type and *mms19*^*ins14/ins14*^ juveniles at 21 and 26 dpf, 21 dpf: wild type, *n* = 9 and *mms19*^*ins14/ins14*^*,n* = 11; 26 dpf: wild type, *n* = 11 and *mms19*^*ins14/ins14*^*,n* = 11. J. Kaplan-Meier survival curves of the wild-type and *mms19*^*ins14/ins14*^ juvenile zebrafish adults are significantly different (χ^2^ = 18.8; *P* value = 10^−5^), wild type, *n* = 28 and *mms19*^*ins14/ins14*^*, n* = 28. K. Normalized head length (by wild-type mean) quantification of *mms19* mutant and wild-type juveniles at 19 dpf, wild type, *n* = 18 and *mms19*^*ins14/ins14*^*, n* = 9. Statistical significances of the differences between the groups in (C), (E), (I), and (K) were calculated using one-way ANOVA including the Tukey’s post-hoc test. Legend: **P < .01; ***P ≤ .001. In all graphs, bars indicate mean values and error bars indicate standard errors of the mean. Scale bars (500 μm or 1 mm) on all zebrafish images indicate their actual sizes.

The functional consequence of the pleiotropic effects of a dysfunctional CIA complex was studied using CRISPR/Cas9-engineered homozygous lines with loss-of-function alleles in *ciao1* (Supplemental Figures S23 and S24) and *mms19* (Supplemental Figure S25) in zebrafish. Phenotypic analysis of the *ciao1*^*del26/del26*^ revealed a reduction in body length (Figures 5A and C), head size (Figures 5B and E), and survival in the homozygous mutants, in which homozygotes *ciao1*−/− began to die at 13 days post fertilization (dpf) with complete loss of the fish by 17 dpf (Figure 5D). Similarly, analysis of the *mms19*^ins14/ins14^ mutants revealed that these mutants were frequently bent, thinner, less mature, and of reduced body length (Figures 5G–I) and head size (Figure 5G). Also, increased mortality of the *mms19*^ins14/ins14^ mutants was observed, starting at about 21 dpf and continuing until the conclusion of the experiment (Figure 5J). Beyond these morphological phenotypes, we confirmed that the loss of *mms19* and *ciao1* leads to a reduction in the level of DPD proteins in zebrafish (Supplemental Figures S26–S29). Therefore, our zebrafish work also showed conserved function of the Ciao1 and Mms19 and the detrimental effect of the deficiencies in these genes on zebrafish development (Figure 5).

## Discussion

The current report links *CIAO1* and *MMS19*, 2 genes coding for CIA proteins, to human disease for the first time and shows that impaired maturation of iron-sulfur containing proteins results in a lethal neurodegenerative phenotype. A large group of proteins, not linked to Fe-S cluster metabolism, proved to be exclusively associated with CIAO1, suggesting that this protein might fulfill additional roles in other biological pathways as well.^19^

Individuals with inherited defects of the mitochondrial ISC machinery are reported in the literature to exhibit metabolic, neurological, and hematological aberrations, which frequently resulted in premature death during childhood.^1,9–12^ The spectrum of these diseases is expanding and currently includes multiple rare human conditions, with distinctive combinations and severities of global and tissue-specific impairments.^9^ *CIAO1* and *MMS19* deficiencies are the first reported inherited disorders of the “cytosolic” CIA system, and the lethal neurodegeneration is consistent with a pleiotropic effect expected from a general failure of cytosolic and nuclear iron-sulfur protein biogenesis. Interestingly, heterozygosity for a variant in *CIAO1* appeared to co-segregate with Alzheimer’s disease in a Japanese family.^23^

The congenital brain malformations, microcephaly and growth delay observed in our *CIAO1* and *MMS19* deficiency patients are in line with the observation that loss of functional Mms19 drastically affected the growth and development of mitotic tissues in *Drosophila* larvae with brains showing a microcephaly phenotype.^24^ In addition, MMS19-beta-GEO gene trap mice are embryonically dead in the preimplantation stage.^22^ Furthermore, Ciao1 has been shown to regulate organ growth and to be mainly required for survival and proliferation of undifferentiated cells in *Drosophila*.^25^ Finally, CIAO1 has been shown to be instrumental in the maturation of the radical *S*-adenosylmethionine (SAM) protein viperin, and the iron-sulfur cluster of viperin is obligatory for its antiviral activity against many different viruses.^4,26^ The critical function of the CIA machinery for antiviral host defense, and its loss may well explain the recurrent severe infections occurring in our CIAO1- and MMS19-deficient patients.

Because of the essential role of iron-sulfur proteins in fundamental biological processes (Supplemental Figure S7), inherited defects in genes encoding iron-sulfur assembly proteins are often detrimental to cell and organism viability,^5,9,22,25^ and thus efforts to study the (metabolic) consequences of disruption of the iron-sulfur cluster assembly pathways have been stymied. Loss of iron-sulfur-containing proteins has been shown to provoke major metabolic reprogramming with the carbon flux being diverted to Fe-S-cluster-independent pathways and the induction of de novo fatty acid biosynthesis.^27^ A similar biochemical phenomenon was observed in the *CIAO1-* or *MMS19*-deficient fibroblasts of our patients, which showed decreased concentrations of the citric acid cycle metabolites, altered carbon fluxes, and a profoundly altered lipid composition.

In human cells, the components of the CIA-targeting complex appear to exhibit a striking preference for particular client proteins,^5,7,19^ and MMS19 is involved in the maturation of apoproteins involved in methionine biosynthesis.^20^ MMS19-deficient fibroblasts contained lower levels of methionine and showed an increased flux of methionine toward the synthesis of cysteine. In addition, CIAO1 and MMS19 are involved in the maturation of multiple proteins required for (organ) growth and genome stability and the impaired growth,^20,25,28^ which might well underlie the decreased proliferation rate of fibroblasts of our patients with a CIAO1 and MMS19 deficiency.

Finally, this report sheds further light on the pivotal role of cofactors in the pathophysiology of rare genetic diseases. Indeed, cofactors are essential for proper enzymatic functions and have the potential to affect multiple pathways. Given the fact that over 30% of all enzymes in the Protein Data Bank require cofactors to function,^29^ it is surprising that disorders in cofactor biogenesis have only been implicated in 6% (*n* = 105; see Supplemental Data) of the currently known 1800+ Inherited Metabolic Diseases.^30,31^ Cofactor genes, however, may not be the most “obvious” candidates for new gene-disease associations because of their broad and variable phenotypic presentation, both clinically and biochemically, and their indirect involvement, ie, via enzymes and proteins. Furthermore, major cofactor deficiencies may be fatal or not at all compatible with life. Therefore, we propose that cofactors should be considered in unsolved cases because they may be the missing link between rare disorder phenotypes and a genetic diagnosis. Meticulous phenotyping and functional analyses can provide the required clues to find the needle in the genomic haystack and thus remain the stepping-stones to solve the unsolved and maximize the success of genomic discovery.

## Supplementary Material

1

2

## Data Availability

Data collected and analyzed for this manuscript are available upon request.

## Abbreviations

CIA: cytosolic iron-sulfur cluster machinery
DPD: dihydropyrimidine dehydrogenase
dpf: days post fertilization
ER: Endoplasmic Reticulum
ISCs: iron-sulfur clusters
MRI: brain magnetic resonance image
PLS-DA: partial least squares discriminant analysis
