# Supplementary material for: CIAO1 and MMS19 deficiency: A lethal neurodegenerative phenotype caused by cytosolic Fe-S cluster protein assembly disorders
Source: Genet Med. Author manuscript; Available in PMC 2025 Feb 3. (PMC11788579; doi:10.1016/j.gim.2024.101104)
Supplement: 1 [file NIHMS2049943-supplement-1.pdf]

SUPPLEMENTARY APPENDIX

SUPPLEMENTARY METHODS

Analysis of gDNA and cDNA coding for DPD

DNA was isolated from whole blood and fibroblasts using the Wizard® Genomic DNA Purification Kit (Promega, The Netherlands). PCR amplification of all 23 coding exons and flanking intronic regions of *DPYD* was carried out as described before.[1] Sequence analysis of genomic fragments amplified by PCR was carried out on an Applied Biosystems model 3730 automated DNA sequencer using the dye-terminator method (Applied Biosystems, Nieuwerkerk a/d IJssel, The Netherlands). The *DPYD* sequences of DPD-deficient patients were compared to those observed in controls and the reference sequence of *DPYD* (Ref Seq NM_000110.4; Ensembl ENST00000370192.8).

Copy number changes were investigated using the MLPA test for *DPYD* (P103, MRC-Holland, Amsterdam, The Netherlands) containing 38 probes for *DPYD* and 9 control probes specific for DNA sequences outside *DPYD*. MLPA was performed as described before.[1,2]

Total RNA was isolated from cultured fibroblasts using Trizol extraction (Invitrogen, Carlsbad, CA, USA). Subsequently, cDNA was prepared using a transcriptor first strand cDNA synthesis kit for RT-PCR (Roche, Mannheim, Germany). PCR amplification of parts of the *DPYD* cDNA sequence was performed using various *DPYD* cDNA primer sets, essentially as described before.[3] Quantitative PCR analysis of cDNA coding for DPD and the reference gene DnaJ heat shock protein family (Hsp40) member B1 (DNAJB1) was performed using the primers 5′-ACATGGCATGGGAGAAAGAG -3′ (forward, located in exon 16) and 5′-GTGACATTTGGGGTCAGCTT-3 (reverse, located in exon 17); 5′-TTCAAGGAGATCGCTGAGGC-3′ (forward) and 5′-TTCTGCCACCGAAGAACTCA-3′ (reverse), respectively. The analysis was performed using the LightCycler® 480 SYBR Green I Master kit (Roche Diagnostics Nederland B·V, Almere, The Netherlands) on a Roche LightCycler® 480 machine and data was analyzed using LinRegPCR software (Ruijter et al., 2009).

Pyrimidine bases and DPD enzyme activity assay

The activity of DPD was determined in a reaction mixture containing 35 mM potassium phosphate (pH 7.4), 2.5 mM MgCl2, 1 mM dithiothreitol, 250 µM NADPH and 25 µM [4 14C]-thymine. Separation of radiolabeled thymine from radiolabeled dihydrothymine was performed by reversed-phase HPLC with online detection of the radioactivity, as described

before.[4] Concentrations of uracil and thymine were determined using reversed-phase HPLC hyphenated with electrospray tandem mass spectrometry.[5,6]

Cell culture

Fibroblasts of the 3 patients, controls and 2 DPD deficient cell lines were routinely cultured in DMEM with 25 mM HEPES, 80 U/ml penicillin/streptomycin, 0.25 µg/ml fungizone and 10% (v/v) fetal calf serum (FCS). Cells were grown at 37°C, 5% CO2 in 95% humidified air. For analysis of DPD activity and protein levels, fibroblasts were cultured in a T175 culture flask for two weeks on the same culture medium. The cells were harvested and the cell pellets were resuspended in 300 µl buffer (35 mM potassium phosphate, pH 7.4 and 2.5 mM $MgCl_2$) and lysed by sonication.[4] Lysates were centrifuged at 20,000×g for 20 min at 4°C and the supernatants were saved for further analysis.

Immunoblot analysis

Samples containing 20-30 µg of protein were fractionated on a 4–12% polyacrylamide gel and transferred to nitrocellulose (GE Healthcare Life Sciences).

Blocking of the membrane was performed with TBS containing 5% (w/v) non-fat-dried milk powder and 0.1% (v/v) Tween 20. The membrane was incubated overnight with a 1:1000 dilution of DPYD antibody (Abnova) in the same buffer. The membrane was washed three times with TBS containing 0.1% (v/v) Tween 20 and incubated for 1 h in TBS containing 0.1% (v/v) Tween 20, 5% (w/v) milk powder and a 1:2000 dilution of polyclonal goat anti-mouse secondary antibody conjugated to HRP (DAKO/Agilent). After rinsing the membrane three times, twice with TBS containing 0.1% Tween20 and once with TBS, detection was performed with ECL Prime Western Blotting Detection Reagent (GE Healthcare Life Sciences) and the membranes scanned with the ImageQuant LAS 4000 imaging system (GE Healthcare Life Sciences).

For analysis of CIAO1, MMS19, PPAT, IRP1, XPD and TUBA membranes were blocked using 50% Odyssey blocking buffer (LI-COR, Lincoln, NE, USA) and 50% PBS (phosphate-buffered saline). Blots were incubated overnight with the first antibody in blocking buffer (50% Odyssey blocking buffer, 50% PBS and 0.1% (v/v) Tween 20). The dilutions of all primary antibodies are summarized in supplementary table S2. Membranes were washed three times with PBS containing 0.1% (v/v) Tween 20 and then incubated for one hour with a Goat or Donkey Anti-rabbit or mouse antibody (LI-COR) in the same blocking buffer as used for the

primary antibodies. After rinsing the membrane three times with PBS containing 0.1% (v/v) Tween 20 the blots were scanned using the LI-COR Odyssey infrared imaging system.

Complementation analysis

Fibroblasts of the 3 patients, a control and *DPYD* deficient cell line were cultured in DMEM with 25 mM HEPES, 80 U/ml penicillin/streptomycin, 0.25 µg/ml fungizone and 10% (v/v) fetal calf serum (FCS). Cells were cultured at 37°C, 5% CO_2 in 95% humidified air in a 175cm² culture flask for two weeks on the same culture medium. Cells to be fused were co-cultured for 2 days before fusion in DMEM with 10% FCS in 75 cm² culture flasks. Equal amounts of cells of each fusion partner were seeded such that confluence was reached prior to the start of the fusion. Fusions were carried out by washing with DMEM, incubation with 4 ml of a solution of 42% ($^w/_v$) polyethylene glycol 1000 (PEG) and 8.75% ($^v/_v$) DMSO in DMEM followed by the addition of 4 ml of 25% (w/v) PEG in DMEM. Subsequently, 14 ml DMEM was added after 2 and 4 min.[7] After 2 more min the solution was aspirated and the cells were washed with DMEM and cultured for 4 additional days in DMEM with 80 U/ml penicillin/streptomycin, 0.25 µg/ml fungizone and 10% (v/v) FCS. The cells were harvested and the cell pellets were resuspended in 200 µl buffer (35mM potassium phosphate, pH 7.4 and 2.5 mM $MgCl_2$) and lysed by sonication.[4] Lysates were centrifuged at 20,000×g for 20 min at 4°C and the supernatants were saved for further analysis.

Genome sequencing

Singleton GS was performed for each proband using the TruSeq DNA PCR-free (350) library kit and HiSeqX sequencer (Macrogen). Sequencing reads were aligned to the human reference genome version GRCh37 using Burrows-Wheeler Alignment tool.[8] Average coverage was calculated using MosDepth[9]: 33X for Patient 1, 31X for Patient 2 and 43X for Patient 3.

Small variants (SNVs and indels) were identified using a previously published bioinformatics workflow[10]. Unbiased genome-wide analysis was performed on nuclear genome and variants prioritized, as previously reported[3,10] based on population frequency and predicted impact: i) variant frequency was less than 0.01 (1%) in population databases with no homozygotes; ii) the variants were not observed in the in-house database to reduce the false positives due to sequencing errors; and (iii) a number of predictors suggested impact.[3,10]

To rule out any additional contributions, the genomes were later re-analyzed and the original findings confirmed using an open-source bioinformatics pipeline based on Genome Analysis Toolkit (GATK)[11] for variant calling and VCFAnno[12] for variant annotation, as previously

described.[13] Furthermore, the nuclear genome was assessed for structural variants using LUMPY[14] and annotated using AnnotSV[15]. Insertions of ALU, LINE1 and SVA mobile elements were called using the Mobile Element Locator Tool.[16] Variants were selected if: i) they were less than 500kb in size, ii) gnomAD frequency was less than 0.01%, iii) AnnotSV predicted the variant as Class 4 (likely pathogenic) or Class 5 (pathogenic). Mitochondrial genome was also analyzed using the MToolBox.[17] Confirmation of the variants and genotypes identified through GS in the patients was performed using Sanger sequencing.

Evidence that heterozygous variants in *CIAO1* occur in trans

PCR was performed (Platinum Taq, Thermo Fisher Scientific) on cDNA of the two *CIAO1* deficient patients with forward primer 5'-AAGGACTCGCTGGTGCTG-3' (between 4 and 21 base pairs from ATG) and reverse primer 5'-GACGCCAGATACGCACAGTA-3' (between 667 and 648 base pairs from ATG). The resulting PCR products (664 base pairs long and containing all 3 variant sites) where ligated in the pGEM-T vector (pGEM®-T Vector System, A3610, Promega). Chemically competent JM109 *Escherichia coli* were transformed with the ligation products and plated on LB agar plates containing 50 µg/ml Ampicillin for the selection of transformed (plasmid containing) JM109 *E.coli*. The next day, multiple colonies were selected for direct sequencing on an Applied Biosystems model 3730 automated DNA sequencer using the dye-terminator method (Applied Biosystems, Nieuwerkerk a/d IJssel, The Netherlands).

Cloning and viral transfection of patients' fibroblasts with wild-type *CIAO1* and *MMS19*

Fibroblasts of the patients were infected with viral particles for stable overexpression of either CIAO1 (pLenti6.3_CIAO1), MMS19 (pLenti6.3_MMS19), or GFP (pLenti6.3_EGFP). Clones of CIAO1 and MMS19 in pLenti6.3 [pLenti6.3 and pLenti6.3EGFP obtained from Iliana Chatzispyrou[18]] where made by first creating Entry Clones with Acc65I/EcoRI respectively Acc65I/XhoI and then sub cloning them in pLenti6.3 with LR Clonase (Gateway Cloning, Thermo Fisher). Production of lentivirus was performed in HEK293T cells using FuGene (Promega) and packaging vectors pMD2.G, pMDLg/pRRE and pRSV-Rev (3rd generation lentiviral packaging (Addgene, Didier Trono Lab Plasmids).[19] Patients' fibroblasts at ~ 90% confluency, cultured in DMEM with 10% FCS where transduced with lentivirus, grown under blasticidine selection (10 µg/ml) for 2 weeks, and further expanded in blasticidine-free medium for four more passages. Subsequently, CIAO1, MMS19 and EGFP overexpressing fibroblasts

where split 1:5 from confluent cells and left for two weeks on culture medium DMEM with 10% FCS. Overexpression of CIAO1 and MMS19 was confirmed by Western blot analysis.

Stability of CIAO1 and MMS19 proteins

CIAO1 and *MMS19* cDNA were cloned in pcDNA3.1Zeo (Thermo Fisher) as a KpnI/XbaI and KpnI/EcoRI fragment, respectively, containing a C-terminal V5 tag (Genscript). Variants were introduced in the expression plasmid containing wild-type human *CIAO1* (pcDNA3.1 Zeo – CIAO1) and *MMS19* (pcDNA3.1 Zeo –MMS19) cDNA, using site-directed mutagenesis. Compatible primers were designed for use with the QuikChange™ Site-Directed Mutagenesis Kit (Agilent). PCR mediated site-directed mutagenesis was performed according to the manufacturer's recommended protocol. All resulting plasmids were sequenced to confirm introduction of nucleotide changes.

HEK293T cells were cultured in Dulbecco's modified eagle's medium with 4.5 g/L glucose, 25 mM HEPES and 584 mg/L L-glutamine (Lonza), supplemented with 10% fetal bovine serum, 100 U/ml penicillin, 100 mg/ml streptomycin and 250 µg/ml fungizone at 37°C in a humidified 5% CO_2 incubator. For transient transfections, cell cultures were setup in six-well plates 24h prior to transfection. HEK293T cells were transfected with pcDNA3.1Zeo-CIAO1 (wild-type or a variant) or pcDNA3.1Zeo-MMS19 (wild-type or a variant) using X-treme GENE HP DNA Transfection reagent (Roche). One day after transfection, cells were treated with 37.5 µg/ml cycloheximide and incubated for another 24h (T=24h). Cells were harvested prior to the incubation with cycloheximide and after treatment with cycloheximide and washed with PBS. After centrifugation at 1000×g for 5 min at 4°C, cell pellets were immediately frozen in liquid nitrogen and stored at −80°C until use. All transfections were performed in quadruplicate. Parental vector (pcDNA3.1Zeo) without insert was transfected as a negative control.

Pellets were resuspended in 200 µl buffer (35 mM potassium phosphate, pH 7.4 and 2.5 mM $MgCl_2$) and lysed by shearing through a needle (25G). Lysates were centrifuged at 20.000×g for 20 min at 4°C. Cell supernatant containing 2.5 µg protein was spotted onto nitrocellulose membrane with Bio-Dot Apparatus (Bio-Rad). The membranes were blocked using Odyssey blocking buffer (LI-COR). Subsequently, the blots were incubated for one hour with a V5 antibody and a rabbit anti α-tubulin antibody (Supplementary table S2) in blocking buffer (50% Odyssey blocking buffer, 50% PBS and 0.1% Tween20). Membranes were washed three times, once with blocking buffer, twice with PBS (0.1% Tween20) and then incubated for one hour with a 1:10.000 dilution of IRDye GaM 800 (LI-COR) and IRDye DaR 680 (LI-COR)

secondary antibodies, in the same blocking buffer as used for primary antibodies. Membranes were washed three times, twice with PBS (0.1% Tween20), once with PBS and then the blots were scanned and dot intensities analyzed using the LI-COR Odyssey infrared imaging system. The dot intensities of CIAO1 and MMS19 proteins were normalized for the corresponding signal of α-tubulin.

In silico structure analysis

Amino acid exchanges and deletions resulting from the variants were manual introduced in the *in silico* crystal structure of CIAO1 (PDB-ID:3fm0; Xu & Min, 2011) and the AlphaFold[20,21]-predicted model of MMS19 using WinCoot.[22] Figure S5 and S6 were generated with PyMol.[23]

Native gel of transiently expressed wild-type and mutant CIAO1 and MMS19

HEK293T cells were cultured in Dulbecco's modified eagle's medium with 4.5 g/L glucose, 25 mM HEPES and 584 mg/L L-glutamine (Lonza, Basel, Switzerland), supplemented with 10% fetal bovine serum, 100 U/ml penicillin, 100 µg/ml streptomycin and 250 µg/ml fungizone at 37°C in a humidified 5% CO_2 incubator. For transient transfections, cell cultures were setup in six-well plates 24 h prior to transfection. HEK293T cells were transfected with pcDNA3.1 Zeo-CIAO1 (wild-type or variants) or pcDNA3.1 Zeo-MMS19 (wild-type or variant) using X-treme GENE HP DNA Transfection reagent (Roche, Basel, Switzerland). Two days after transfection, cells were harvested and washed with PBS. After centrifugation at 100×g for 5 min at 4°C, cell pellets were resuspended in 200 µl isolation buffer (35 mM potassium phosphate, pH 7.4 and 2.5 mM MgCl2) and lysed by shearing through a needle (25G). Lysates were centrifuged at ≥ 11,000 rpm for 20 min at 4°C, and then supernatant protein concentrations were directly quantified.

Native gel electrophoresis was performed using 4–16% NativePAGE™ Novex® Bis-Tris Gels (Thermo Fisher). Supernatant samples were prepared in sample buffer (50 mM Bis Tris, 6N HCL, 50 mM NaCl, 10% glycerol and 0.001% Ponceau S, pH 7.2), and 3 µg protein loaded.

Electrophoresis was performed at 150 V for 90 min at room temperature. Gels were transferred onto PVDF membrane for 1h at 25V. Membranes were blocked using Odyssey blocking buffer (LI-COR). Subsequently, blots were incubated for one hour with a 1:5000 dilution of a V5 Tag Antibody antibody (R960-25, Thermo Fisher), a 1:1000 dilution of CIAO1 Rabbit mAb (D1B4G, Cell Signaling) or a 1:1000 dilution MMS19 Rabbit mAb (D5J8J, Cell Signaling) in blocking buffer (50% Odyssey blocking buffer, 50% PBS and 0.1% Tween). Membranes were washed three times (two times with 50% Odyssey blocking buffer, 50% PBS

and 0.1% Tween and one time with PBS and 0.1% Tween) and then incubated for one hour with a 1:10,000 dilution of IRDye800 conjugated goat anti-mouse secondary antibody (LI-COR) or 1:10,000 dilution IRDye800 conjugated goat anti-rabbit secondary antibody (LI-COR) (Supplementary Table S2. Antibodies used for immunoblotting) in the same blocking buffer as used for primary antibodies, with 0.01% SDS. Blots were washed three times (two times with PBS and 0.1% Tween and one time with PBS). Blots were scanned and band intensities analysed using the LI-COR Odyssey infrared imaging system.

Co-immunoprecipitation

HEK293 cells were cultured in 10-cm petri dishes and transfected with pcDNA3.1 Zeo-MMS19 (wild-type or variant) or the empty vector using X-treme GENE HP DNA Transfection reagent (Roche, Basel, Switzerland). After 2 days cells were collected in PBS and washed 2 more times with PBS. Pellets where resuspended in 100 µl PBS with 1 mM dithiobis[succinimidylpropionate] (DSP) for crosslinking of protein complexes and incubated for 2 hours on ice, according to the manufacture protocol (Pierce DSP, Thermo Scientific, Pierce Biotechnology, Rockford, USA). Suspensions were diluted 10-fold with RIPA/tris buffer. Immunoprecipitations were carried out by overnight incubation with 10 µg of agarose conjugated V5 antibody (Bethyl Laboratories, Montgomery, USA). The agarose beads were washed 3 times with TBS/0.1% ($^{w}/_{v}$) Tween20 and eluted with 20 µl 2-fold concentrated SDS-Page sample loading buffer and used for Western blotting of CIAO1 and MMS19 (Supplementary table S2).

Cell proliferation

Cells were plated in 96-wells plates and allowed to adhere overnight, after which the medium was replaced by medium containing different concentrations of FCS (0.2%, 1% or 10%) After 1 and 5 days, the quantification of viable cells was assessed using an MTS assay according to the manufacturer's protocol (Promega G5421, Madison, WI, USA).

DNA Repair

One day prior to γ-radiation, cells were seeded on glass coverslips in 6 well plates with 2 ml DMEM medium. The cells were exposed to a 2 Gy dose of gamma radiation from a Cs-137 source. After recovery in a 37°C CO_2 incubator for 30, 120 or 300 min, cells were washed two times with PBS and fixed with 2% paraformaldehyde at room temperature. After fixation, cells

were washed 3 times with PBS. The DNA double-strand breaks were visualized by immunodetection of histone H2AX phosphorylation, resulting in γ-H2AX, which expands through the chromatin on both sides of a double-strand break, essentially as described before.[24]

Immunofluorescence

Cells were plated on glass coverslips with 2 ml DMEM medium and incubated overnight at 37°C in a CO_2 incubator. The cells were washed tree times with PBS and fixed with 2% paraformaldehyde at room temperature for 20 min. The cells were washed twice with PBS, permeabilized with 0.1% Triton X-100 in PBS for 10 min, and blocked with PBS containing 5% Normal Goat Serum (NGS) for 1 hour at room temperature to prevent nonspecific binding of antibodies. Cells were incubated for 1 or 2 hours at room temperature with the indicated primary antibody diluted in the 5% NGS blocking buffer. The primary antibodies against Protein Disulfide Isomerase (Endoplasmic Reticulum), RCAS1 (Golgi), and Phalloidin (Cytoskeleton) were obtained from Cell Signaling Technology. The primary antibodies against CD63 (MX-49.129.5) [multivesicular bodies] and TOM20 (FL-145) [mitochondria] were obtained from Santa Cruz Biotechnology, INC. After the incubation, the coverslips were washed 3 times with 0.1% triton in PBS and once with PBS. Subsequently, the cells were incubated with Alexa 488-conjugated Goat anti-mouse IgG or Goat anti-rabbit secondary antibodies at room temperature for 1 hour. The cells were washed tree times with PBS and a 1:20 dilution of Phalloidin Alexa 555, in PBS, was added for 30 min. The cells were washed tree times with PBS and mounted with Prolong Gold Antifade mountant with DAPI. Images were acquired with a Leica DM6 widefield microscope.

Transmission Electron Microscopy

Electron Microscopy was performed on fibroblasts cultured in a T75 culture flask for two weeks on the same culture medium. One day prior to harvesting of the cells, the culture medium was refreshed. The epon-samples were fixed in McDowell containing paraformaldehyde and glutaraldehyde (Polysciences, Inc., Warrington, PA, USA) and post-fixed with 1% osmium tetroxide (OsO4, Electron microscopy sciences, Hatfield, PA, USA; in cacodylate buffer). Subsequently, the samples were dehydrated in an alcohol series and embedded into Epon (LX-112 resin Ladd research, Williston, VT, USA). The samples for immuno-EM according to Tokuyasu were fixed overnight in 2% paraformaldehyde and 0.5% glutaraldehyde in 0.1 M PHEM and stored in 0.1M PHEM and 0.5% PFA. Before cryo-fixation, the cells were washed and placed in 2% gelatin-PBS for 30 min, scraped then placed in 12% gelatin-PBS and

incubated overnight in 2M sucrose and snap-frozen in liquid nitrogen. Ultrathin (70 nm) epon sections of the samples were cut and collected on Formvar-coated grids, counterstained with uranyl acetate and lead citrate. Immuno-EM sections of 60 nm were cut at -120 ºC and immuno-gold labelled with CD63 (Santa Cruz Biotech technology, inc.) or LAMP (CD107a 1D4B Pharmingen) and protein A conjugated with 10 nm gold particles (Utrecht University) and stained with Uranyl Acetate. Sections were examined with a FEI Tecnai-12 G2 Spirit Biotwin electron microscope (Fei, Eindhoven, The Netherlands), images were taken with a Veleta camera using Radius software (EMSIS, Münster, Germany).

Proteomics

Samples were essentially prepared as described previously[25] with some modifications. In short cell pellets were lysed by sonification in 500 µl of 100 mM ammoniumhydrogencarbonate (ABC) and 1% (w/w) Sodium dodecyl sulfate (SDS). Protein concentrations of cellular lysates were determined using the BCA-assay (Thermo) according to the manufacturer's protocol. Volumes equivalent to 10 ug (based on the BCA-assay for the 37° C sample) were reduced and alkylated with 10 mM tris-carboxy-ethyl-phosphine and 40 mM chloroacetamide for 15 min at 60° C. Samples were cooled to room temperature and cleaned by single pot solid phase sample preparation essentially as described previously[25] and subsequently digested with 1:20 (enzyme to substrate by weight) of proteomics grade trypsin (Promega) overnight at 37°C. Peptide samples were subsequently cleaned by solid phase extraction using OASIS HLB extraction plates (Waters) according to the manufacturer's protocol and resulting samples were dried in a vacuum centrifuge. Dried peptide samples were reconstituted in 0.1% formic acid.

Dry peptide samples were reconstituted with 1% formic acid in water (ULCMS grade, Biosolve) and 200 ng equivalent was injected onto a C18 column (75 µm, 250 mm, 1.6 µm particle size, Aurora, Ionopticks, Fitzroy, Australia) kept at 50°C by an on source column oven (Sonation, Biberach, Germany). Using a NanoRSLC Ultimate 3000 UHPLC system (Thermo Scientific, Germeringen, Germany), peptide samples were separated by a gradient (solvent a: 0.1% formic acid in water, solvent b: 0.1% formic acid in acetonitrile). Peptides were loaded at 400 nl/min for 2 min in 3% solvent B and separated by a multi-step gradient, to 6% solvent B at 5 min, 21% solvent B at 21 min, 31% solvent B at 33 min, 42.5% solvent B at 36 min and 99% solvent B at 37 min held for 7 min before returning to initial conditions (Solvent A: 0.1% formic acid in water, Solvent B: 0.1% formic acid in acetonitrile) until 60 min (total run time). Eluting peptides were electrosprayed by a captive-spray source, into a timsTOF-pro (trapped ion mobility spectroscopy, quadrupole time of flight mass spectrometer, Bruker, Bremen,

Germany). using the following settings: precursor scan ranged from 100 to 1700 m/z and a tims range of 0.6–1.6 V.s/cm2 in PASEF mode. A total of 10 PASEF MS/MS scans were collected with a total cycle time of 1.16 s.

Resulting mass spectra were analysed by Maxquant (1.6.10.43) searching against the proteome database of Homo Sapiens (Uniprot, 10/2019). Search parameters were: enzyme set as trypsin allowing for 2 missed cleavages, carbamidomethylation at cysteine as a fixed modification and oxidation at methionine and n-terminal acetylation as variable modifications. Protein false discovery rate was kept at 1% using a target-decoy database. Identified protein-groups were further processed using Perseus (2.0.3.0) to do differential analysis (Student's-T) and generate figures. DAVID (Sherman 2021) was used to look for functional enrichments amongst regulated proteins.[26]

Metabolomics

Metabolomics analysis was performed as described previously.[27,28] The HPLC system consisted of an Acquity binary HPLC pump, a vacuum degasser, a column temperature controller, and an auto sampler (Waters, Milford, MA, USA). The column temperature was maintained at 30°C. 5 µL of the metabolite extract was injected onto a SeQuant 100 x 2.1 mm ZIC-cHILIC column, 3 µm particle diameter (Merck, Darmstadt, Germany). An Impact II QTOF and TimsTOF pro (Bruker Daltoniks) mass spectrometer was used in the negative and positive electrospray ionization mode. In both the negative and positive ionization mode, mass spectra of the metabolites were obtained by continuous scanning from m/z 50 to m/z 1200 with a resolution of 50000 FHMW. The timsTOF pro was operated in full scan and PASEF mode. At least one internal standard per major class was used to normalize the intensities of the metabolites. The identified metabolites are classified according to the Human Metabolome Database (HMDB; www.hmdb.ca).

Data were analyzed using Bruker Metaboscape 2022 (8.02 (build 11494)) and TASQ 2021b (2021.1.2.452) software. All reported metabolite intensities were normalized to dry tissue weight, as well as to internal standards with comparable retention times and response in the MS. Metabolite identification was based on a combination of accurate mass, (relative) retention times and fragmentation spectra, compared with the analysis of a library of standards. For metabolomic data, analyses were performed with R (https://www.r-project.org) version 3.5.1 and Bioconductor version 3.7.[29] Partial least-squares discriminant analysis (PLS-DA) and metabolite variable importance to projections (VIP) scores were calculated using the R package MixOmics version 6.6.2.[30] Significance was assessed using an empirical Bayes moderated t

test on log2 transformed data within limma's linear model framework, taking participants and their regimen into account.[31,32] Unless implemented through an aforementioned R package or base R graphics, visualization of data was performed using ggplot2.[33]

Flux Metabolomics

Fibroblasts were plated in 60 mm dishes, at a confluency of 50% in a total volume of 4 ml DMEM supplemented with 10% FCS, 25 mM HEPES and 80 U/ml penicillin/streptomycin/fungizone and were allowed to adhere overnight. Subsequently, the medium was replaced by DMEM medium, deprived of glucose, glutamine, sodium pyruvate and phenol red (Gibco, A14430-0, Landsmeer, the Netherlands) and supplemented with 25 mM HEPES, 80U/ml penicillin/streptomycin/fungizone, 50 µM carnitine, 5 mM D-glucose -U-$^{13}C_6$ (Buchem, CLM-1396) or 1 mM L-glutamine -$^{13}C_5$, $^{15}N_2$ (Buchem, CNLM-1275) or 50 µM Oleic Acid-$^{13}C_{18}$ (Cambridge Isotope Laboratories, CLM-460-PK). The final concentration of added labeled or unlabeled glucose, glutamine and oleic acid to each well was 5 mM, 1 mM and 50 µM, respectively. The labeling with L-Methionine-$^{13}C_5$ was performed in DMEM medium deprived of L-methionine and L-cystine and supplemented with 4 mM glutamine, 1 mM pyruvate and 10% FCS.

After 18 hours of incubation, the medium was removed and the dishes were washed on ice three times with ice-cold 0.9% (w/v) NaCl. Subsequently, 250 µL methanol (-20°C) was added to each dish followed by 250 µl ice-cold Milli-Q water. The cell suspension was transferred to a 2 ml Eppendorf cup and the dish was washed with 500 µl 50% methanol (-20°C) and added to the cell suspension in the 2 ml Eppendorf cup. Chloroform (1 ml) was added to each sample and the solution was sonicated (40 Joule/Watt·sec, 7.5-Watt output). After centrifugation [20,000 g (4°C)], the "polar" top layer was transferred to a new 1.5 mL tube and dried to dryness in a vacuum concentrator (60°C, 1.5 h). The final pellet was dissolved in 50 µl 60% methanol, centrifuged at 20,000 g (4°C) and 40 µl of the supernatant was saved for further analysis. For the analysis, we used the metabolomics method as is described in the above section.

Lipidomics

Fibroblasts of the 3 patients and 7 controls were cultured on DMEM medium for two weeks, as described above. Lipidomics analysis was performed, essentially as described before.[34,35] Briefly: In a 2 mL tube, the following amounts of internal standards dissolved in 1:1 (v/v) methanol:chloroform were added to each aliquot of 20 µL fibroblasts sample:

Bis(monoacylglycero)phosphate BMP(14:0)2 (0.2 nmol), Ceramide-1-phosphate C1P (d18:1/12:0) (0.127 nmol), D7-Cholesteryl Ester CE(16:0) (2 nmol), Ceramide Cer(d18:1/12:0) (0.118 nmol), Ceramide Cer(d18:1/25:0) (0.130 nmol), Cardiolipin CL(14:0)4 (0.1 nmol), Diacylglycerol DAG(14:0)2 (0.5 nmol), Glucose Ceramide GlcCer(d18:1/12:0) (0.126 nmol), Lactose Ceramide LacCer(d18:1/12:0) (0.129 nmol), Lysophosphatidicacid LPA(14:0) (0.1 nmol), Lysophosphatidylcholine LPC(14:0) (0.5 nmol), Lysophosphatidylethanolamine LPE(14:0) (0.1 nmol), Lysophosphatidylglycerol LPG(14:0) (0.02 nmol), Phosphatidic acid PA(14:0)2 (0.5 nmol), Phosphatidylcholine PC(14:0)2 (2 nmol), Phosphatidylethanolamine PE(14:0)2 (0.5 nmol), Phosphatidylglycerol PG(14:0)2 (0.1 nmol), Phosphatidylinositol PI(8:0)2 (0.5 nmol), Phosphatidylserine PS(14:0)2 (5 nmol), Sphinganine 1-phosphate S1P(d17:0) (0.124 nmol), Sphinganine-1-phosphate S1P(d17:1) (0.125 nmol), Ceramide phosphocholines SM(d18:1/12:0) (2.129 nmol), Sphingosine SPH(d17:0) (0.125 nmol), Sphingosine SPH(d17:1) (0.125 nmol), Triacylglycerol TAG(14:0)2 (0.5 nmol) and 1.5 mL 1:1 (v/v) methanol:chloroform was added before thorough mixing. Each sample was then centrifuged for 10 min at 14.000 rpm. Supernatant was transferred to a glass vial and evaporated under a stream of nitrogen at 60°C. The residue was dissolved in 150 µL of 1:1 (v/v) methanol:chloroform.

Lipids were analyzed using a Thermo Scientific Ultimate 3000 binary HPLC coupled to a Q Exactive Plus Orbitrap mass spectrometer. For normal phase separation, 2 µL of each sample was injected onto a Phenomenex® LUNA silica, 250 * 2 mm, 5µm 100Å. Column temperature was held at 25°C. Mobile phase consisted of (A) 85:15 (v/v) methanol:water containing 0.0125% formic acid and 3.35 mmol/L ammonia and (B) 97:3 (v/v) chloroform:methanol containing 0.0125% formic acid. Using a flow rate of 0.3 mL/min, the LC gradient consisted of: 10% A for 0-1 min, reach 20% A at 4 min, reach 85% A at 12 min, reach 100% A at 12.1 min, 100% A for 12.1-14 min, reach 10% A at 14.1 min, 10% A for 14.1-15 min. For reversed phase separation, 5 µL of each sample was injected onto a Waters HSS T3 column (150 x 2.1 mm, 1.8 µm particle size). Column temperature was held at 60°C. Mobile phase consisted of (A) 4:6 (v/v) methanol:water and B 1:9 (v/v) methanol:isopropanol, both containing 0.1% formic acid and 10 mmol/L ammonia. Using a flow rate of 0.4 mL/min, the LC gradient consisted of: 100% A at 0 min, reach 80% A at 1 min, reach 0% A at 16 min, 0% A for 16-20 min, reach 100% A at 20.1 min, 100% A for 20.1-21 min. MS data were acquired using negative and positive ionization using continuous scanning over the range of m/z 200 to m/z 2000. Data were analyzed using an in-house developed lipidomics pipeline written in the R programming language (http://www.r-project.org). All reported lipids were normalized to

corresponding internal standards according to lipid class and normalized to the total amount of protein in each sample. Lipid identification was based on a combination of accurate mass, (relative) retention times and the injection of relevant standards.

Lipids were identified using an in-house database of lipids from various lipid classes, with matching ion mass and expected retention times. To identify discriminatory lipids and their importance for the difference between patients and controls and/or two patient groups, several statistical tests were used. Orthogonal partial least squares regression with Discriminant Analysis (OPLS-DA) was used to calculate Variable Importance in Projection (VIP) scores, which were used to estimate the importance of each lipid in the differentiation between the groups. A lipid with a VIP score equal or greater than 1 was considered important in the given PLS-model. A t-test was performed to assess significant differences in lipid levels between the combinations of two groups. P-values lower than 0.05 were considered significant. The Bonferroni-Holm-correction was used to adjust p-values for multiple testing. The uncorrected p-values, together with the fold change, are plotted in the volcano plots. For the lipidomics data, effects in individual lipids were not investigated but the focus was on trends in fatty acid chain length or degree of saturation. All analyses were performed in R.

Network analysis

We used the human Genome-Scale Metabolic Model (GSMM) HMR2[36] to determine the biochemical interconversions between the metabolites that were measured on our metabolomics platform. All computations described in this section were performed in MATLAB R2019b. To facilitate the mapping of metabolomics data to the GSMM, we enriched the model with compound synonyms and external identifiers from the ChEBI database[37], where ChEBI identifiers and synonyms of conjugate acids and bases were also included. Of the 133 metabolites that were quantified, 127 mapped properly to the GSMM. All reactions and compounds in HMR2 were checked for mass and redox balance and were adjusted when necessary.

Biochemical interactions between metabolites were determined by converting the GSMM into a weighted directed graph where nodes represent metabolites and edges represent reactions. Subsequently all reaction paths between the measured metabolites that involved one or two reaction steps were determined using a generic path finding algorithm that was developed *in house*. To ensure that the reaction paths represented relevant biochemical conversions, each path was checked for stoichiometric and thermodynamic consistency. In addition, only substrate-product mappings were considered that involved the transfer of

carbon-based moieties. As a consequence, half-reactions involving the transfer of electrons, amino or phosphate groups were decoupled from the main reaction in the path finding procedure. For example, in the reaction NADH + pyruvate <=> NAD+ + lactate, only NADH and NAD+ are linked in the graph and pyruvate and lactate are linked. Likewise, in the reaction glutamate + pyruvate <=> AKG + alanine, only glutamate and AKG are linked, and pyruvate and alanine are linked. In this way the creation of crowded, highly connected networks in which most metabolites are connected to a few hub metabolites such as H+, H2O, ATP and NADH was prevented.

The weight of the edges in the GSMM-based network was set to 1 for all reactions, except for transporter reactions that transferred compounds over the cellular membranes and (half) reactions that involved uniquely produced metabolites; both these types of reactions were assigned a weight of zero. A consequence of the second exception is that linear reaction chains in which the intermediates were not produced by other reactions were counted as a single reaction step during the path search. Since our platform covered mostly metabolites from central metabolism, we also added to the list metabolites from the glycolysis, pentose phosphate pathway and Krebs cycle that could not be quantified, in order to prevent gaps in the traditional pathways.

To facilitate the inspection of the network analysis results, an interactive HTML/JavaScript document was written that imported and visualized the GSMM-based metabolite network and the reaction paths corresponding to the network links. Reaction information was enriched by importing tissue-specific gene expression from the Human Protein Atlas (HPA)[38] and Genotype-Tissue Expression (GTEx)[39] project. Finally, in order to facilitate the interpretation of the results and see what alterations in the metabolome could potentially be due to the reduced activity of enzymes containing iron-sulfur clusters, we imported the list of human proteins reported by Lill and Freibert [40] and highlighted the corresponding reaction paths in the network.

Ciao1 and *Mms19* mutant zebrafish

Zebrafish husbandry and maintenance

Zebrafish experiments and husbandry were performed under standard protocols[41] in accordance with the standards of the University of Ottawa Animal Care Committee (approval number: CHEOe-3246-R2). The *mms19* mutant was generated and analyzed in the *casper* strain background[42] while the *ciao1* mutant was generated and analyzed in the AB background.

Zebrafish embryos were maintained at 28.5°C during development and as adults. Embryos were grown in 1x E3 medium (5 mM NaCl, 0.17 mM KCl, 0.33 mM CaCl2, 0.33 mM $MgSO_4$).

Generation of the ciao1 and mms-19 mutant zebrafish using CRISPR/Cas9

ciao1 mutants: CRISPR/Cas9 variants were generated in WT zebrafish as described elsewhere.[43] The *ciao1*-targeting single-guide RNA (sgRNA) template plasmid was generated by annealing oligonucleotides and ligation of the double-stranded DNA in the plasmid DR274 (Addgene, code 42250; Watertown, MA). The CRISPR target sequence (5′-GGCTCCATCTGGAAGCTTAC-3′) and oligonucleotide design were performed using ZiFit software.[44] The sgRNAs were transcribed from linearized template plasmids (Ambion MEGAscript T7/SP6) and purified (Promega, Madison, WI; Wizard SV Gel and PCR Clean-Up kit). The Cas9 protein was obtained from New England Biolabs (Beverly, MA). Fertilized one-cell-stage zebrafish eggs were injected with a mix containing ~300 ng/µl Cas9 protein and 15 ng/µl sgRNA. Embryos were raised and a portion of them (five pools of five embryos) were genotyped by heteroduplex melting assay (HMA) as described in.[45] Briefly, primers flanking the CRISPR target site (forward sequence: "Ciao1_FW," 5′-AGTTGATCTGACATGTTTGTTATGTG-3′, reverse sequence: "Ciao1_RV," 5′-GACACTGTATTCTCCATATTTCTGAGG-3′) were used to amplify a segment of 177 bp. The PCR product was denatured and annealed. It was expected to contain a mixture of insertion/deletion variants and WT alleles, which can form heteroduplex and homoduplex DNA. The remaining embryos were raised to adulthood (F_0 generation) and backcrossed with WT fish. DNA was extracted from F_1 embryos to check for F_0 founders carrying specific variants by HMA-PAGE. F_1 fish were raised to adulthood and heterozygous mutants were identified by fin clipping, followed by DNA extraction and HMA-PAGE. Potential mutants were sequenced allowing the identification of specific variants. Populations of heterozygous F_1 fish carrying the same variant were identified; the most common was a 26-bp deletion (Fig. S23 and Fig. S24). F_1 heterozygous fish were backcrossed to WT fish to further eliminate potential off-target effects generated by the Cas9 nuclease. For the experiments described in this paper, we used crossings of F_2 heterozygous fish obtaining F_3 offspring containing a homozygous 26-bp deletion in exon 3 of *ciao1*.

mms19 mutants: The *mms19* gene was targeted with a single guide RNA (sgRNA) identified in exon 5 (ATGGACACCGCTGAACCGGG) and Alt-R® S.p. Cas9 Nuclease V3 (Integrated DNA Technologies, 1081058). The oligo containing T7 promoter, sgRNA spacer and a scaffold overlap region was synthesized (Table S3). sgRNAs were generated by performing an overlap-extension PCR of the sense sgRNA oligos each combined with *Rev_sgRNA_scaffold* oligo. sgRNA template synthesis reactions were set up using *Taq* DNA polymerase (ABM, G009) by combining 10 µl of 10× buffer, 6 µl of 25 mM $MgSO_4$, 2 µl of 10 mM dNTP, 5 µl of each oligo at 25 µM, 71 µl water and 1.5 µl of Taq. The PCRs were run with a short program: 94°C for 5 min; 5 cycles: 94°C for 30 s, 55°C for 30 s, 72°C for 30 s. The resulting PCR products were purified using QIAGEN Gel Extraction kit (QIAGEN, 28704) and used for *in vitro* transcription using MEGAshortscript T7 kit (Thermo Fisher Scientific, AM1354), purified by ethanol precipitation and resuspended at 1 µg/µL. Cas9 protein at 500 ng/µL was mixed with the sgRNA at 200 ng/µL, KCl at 300 mM, incubated at 37°C for 5 min, injected into zebrafish eggs and grown up.

Genotyping ciao1 and mms19 zebrafish

The methods for genotyping zebrafish have been described previously.[43]

ciao1: A Taq-based PCR reaction using the primers *ciao1_T2_FW* (AGTTGATCTGACATGTTTGTTATGTG, *ciao1_T2_REV* (GACACTGTATTCTCCATATTTCTG) followed HindIII (NEB, R3104S) digestion of PCR product aliquots (7 µL per 20 µL digestion reaction) was used to precisely genotype wild-type and/or 26-bp deletion mutant alleles using the DNA extracted from larval fins at 3–5 days post fertilization (dpf) (Fig. S23). Samples were then analyzed on a 2% agarose gel, which allowed for the distinction of the 151 bp mutant band, heteroduplex bands (177/151 nt) and digested wild-type bands (98 and 79 bp) (Fig. S24).

mms19: For the *mms19* mutants (Fig. S25), we initially employed heteroduplex mobility assay (HMA) at 8% of polyacrylamide to identify heterozygotes for a pathogenic variant as described previously[46] after the touch-down PCR (tdPCR) method with *mms19_EX45_del_for* and *mms19_EX45_del_rev* primers (Table S3) with a Taq polymerase: 94°C for 3 min; 10 cycles: 94°C for 30 s, *61*°C (with 1°C decrease every cycle), 72°C for 30 s, 25 cycles: 94°C for 30 s, *51*°C, 72°C for 30 s. Briefly, the PCR products were run on polyacrylamide gels and variant presence was visualized based on the presence of heteroduplex bands. To identify the precise

nature of the variant, the PCR products for HMA described above were submitted for standard Sanger sequencing and the chromatogram traces were analysed by PolyPeakParser (http://yosttools.genetics.utah.edu/PolyPeakParser/).[47] These alleles were later confirmed by sequencing of PCR products from F_2 homozygous mutant embryos. Concurrently, the *mms19* genotype had been confirmed by BslI (NEB, R0555S) digestion of PCR products according to the manufacturer's instructions (Fig. S25). Since both mutant alleles introduce a BslI site, digestion of homozygous mutant samples is almost complete, whereas heterozygotes produce partial digestion, and wild-type samples fail to be digested (Fig. S25). This method was also used for larval genotyping after fin clipping at 3 dpf.[48]

RNA extraction and expression analysis

ciao1: Total RNA was extracted from three pools of five WT, *ciao1*$^{+/-}$ or *ciao1*$^{-/-}$ larvae using the reagent QIAzol (QIAGEN, Valencia, CA) (each pool considered one biological replicate). First-strand-complementary DNA was synthesized from 1 µg total RNA using the iScript kit (Bio-Rad). Quantitative real-time PCR was conducted using the following primer pairs: *gapdh* (5′-TGTTCCAGTACGACTCCACC-3′ and 5′-ACCTGCATCACCCCACTTAA-3′), *mms19* (5′-GTTTGCGGAGTTTCTGTTAC-3′ and 5′-GGAAAACCTCTCTCCGTATT-3′), *dpyd* (5′-TATCAATATTGGTGGGCTTC-3′ and 5′-GGATTTCTGAAGTGCTCAAG-3′), and *ciao1* (5′- TAGGCTCCATCTGGAAGCTTAC-3′ and 5′- AAACGTAAGCTTCCAGATGGAG-3′), with iQ SYBR Green Supermix (Bio-Rad). Relative mRNA levels were normalized to glyceraldehyde-3-phosphate dehydrogenase mRNA levels using the ΔΔCT method. The PCR amplification was performed in three technical replicates. One-way analysis of variance (ANOVA) was used to evaluate the statistical significance of the differential mRNA levels between the six biological replicates of wild-type, *ciao1*$^{+/-}$ and *ciao1*$^{-/-}$ siblings.

mms19: Each total RNA sample was extracted from 30-50 zebrafish embryos homogenizing them in 500 µL Trizol reagent (Thermo Fisher Scientific, 15596026) using 1 mL syringe and 21G needle and RNA was purified from lysates according to the Phasemaker Tubes protocol manual (Thermo Fisher Scientific, A33248). For cDNA synthesis, a 4-µg aliquot of total RNA was DNAse-treated using TurboDNA-free kit (Thermo Fisher Scientific, AM1907) according to the kit instructions. cDNA was produced by mixing 10 µL of DNAse-treated RNA with 4 µL of 2.5 mM dNTP and 2 µL 100 µM oligo-dT(15-18) (Integrated DNA Technologies), heating at 70 °C for 10 min and cooling on ice. We then added 2 µL of M-MuLV buffer (NEB,

M0253S), 0.25 μL of Protector RNAse Inhibitor (Roche, 03335399001), 0.25 μL of M-MuLV reverse transcriptase (NEB, M0253S) and 1.6 μL of water. The synthesis reaction was incubated at 42 ºC for 1 hour and 10 min at 90 ºC. cDNA was generated from pooled progeny of *mms19*$^{+/\Delta5}$ and *mms19*$^{+/ins14}$ fish as described above. Variant reporter cDNA fragments containing a 5'UTR and the first 271 codons were amplified from these cDNA samples using *PacI-T3_mms19_mutrep_for* and *AscI-mms19_mutrep_2_rev* primers (Table S3) by Q5 High-Fidelity 2X Master Mix (NEB, M0492S) at 67 ºC with a 30-second extension. The resulting PCR products were digested with PacI (NEB, R0547S) and AscI (NEB, R0558S), purified and cloned into the pCS2+MCS-P2A-sfGFP vector (Addgene, 74668) as described previously.[46] The resulting bacterial clones were genotyped by a colony PCR procedure (1 colony resuspended in 100 μL water followed by boiling 10 μL for 5 min at 95ºC) with a Taq-based PCR using *mms19_ISH_for* and *AscI-mms19_mutrep_2_rev* primers run according to its standard program at the 57 ºC annealing temperature (Table S3). The PCR products (7 μL per digestion) were digested with the BslI enzyme resulting in digestion patterns for wild-type (0.61 and 0.276 kb) or mutant (0.41, 0.276 and 0.195 kb) clones. Three clones of each genotype were Sanger-sequenced using SP6 and insert-specific primers above. The resulting pCS2+mms19-wt-P2A-sfGFP, pCS2+mms19-ins14-P2A-sfGFP and pCS2+mms19-delta5-P2A-sfGFP vectors were prepared as mini-preps and injected into 1-cell stage embryos at 20 ng/μL.

cDNA prepared from wild-type embryos (16, 24 and 48 hpf) was used for amplification of the probe template using *mms19_ISH-3_for* and *T7_mms19_ISH-3_for* primers (Table S3) by Q5 polymerase at 66 ºC with a 30-second extension. The probe was synthesized from the template PCR product by DIG RNA Labeling Kit (SP6/T7) (Roche, 11175025910) according to the kit instructions. Whole-mount *in situ* hybridization (WMISH) was carried out according to the protocol by Lauter *et al.*[49] except that the detection step was performed using Anti-Digoxigenin-AP, Fab fragments (Roche, 11093274910) at 1:2500 dilution in the blocking buffer and the staining step was done with BCIP (Roche, 11383221001) and NBT (Roche, 11383213001) reagents diluted in the staining buffer. The stained embryos were then fixed in 4% PFA for 30 min, washed in PBST and embedded into 80% glycerol for imaging.

Phenotyping ciao1 and mms19 zebrafish.

We followed survival of wild-type and mutant larvae from 6 to 36 dpf and recorded the times when mortalities were detected in respective groups. Live embryos at the end of observations were also recorded. These records were then used for survival analysis using R statistical

computing language with ‘survival’, ‘survminer’, ‘dplyr’ and ‘ggplot2’. The statistical significance was determined by the survdiff function implementing the log-rank or Mantel-Haenszel test with χ^2 value used to determine the P-value of significance[50] (https://CRAN.R-project.org/package=survival).

Larvae and juveniles were anesthesized with 0.02% Tricaine and photographed using a stereomicroscope (Nikon, Garden City; SMZ1500 or Zeiss Axio Zoom V16 microscope). Standard lengths (distance from the anterior tip of head to the base of the caudal fin) and brain size were measured manually using imageJ software for *ciao1* or using Fiji plugins.[51] The figures were prepared using GIMP and Inkscape from microscopy images and graphs.

Western blotting

Western blotting was performed according to a standard protocol [52] with the following details. The zebrafish larvae at stages between 6-7 dpf (groups of 10) and 26 dpf (groups of 5) of either wild-type or mutant genotypes were homogenized using 21 G syringes in 1x RIPA buffer (Millipore Sigma, 20-188) containing 0.1% SDS, 1x cOmplete, Mini Protease Inhibitor Cocktail (Roche, 4693124001) as well as 1mM PMSF, 10 mM NaF, 2 mM Sodium Orthovanadate and 20 mM Sodium beta-glycerophosphate. Protein lysates were measured using Pierce BCA Protein Assay kit (Thermo Scientific, 23227) and 1/3 volume of 4x Laemmli sample buffer (Bio-Rad, 1610747) with β-mercaptoethanol was added, and samples were boiled at 95ºC for 5 min. The gels were prepared using TGX Stain-Free FastCast Acrylamide 10% kit (Bio-Rad, 1610183), and ~20 µg of protein was loaded into each well. The gels were transferred to the Odyssey Nitrocellulose membranes (LI-COR, 926-31092). The blots were blocked with 5% milk in TBST and probed using an anti-DPYD (1:1000, MyBioSource, MBS9607163), anti-POLD1(1:1000, MyBioSource, MBS9605453) and beta-actin (1:1000, Cell Signaling Technology, 5125) antibodies at 4ºC overnight with gentle agitation. The primary antibodies were detected with anti-Rabbit-HRP secondary antibodies (1:1000, ThermoFisher Scientific, 65-6120) at room temperature for 1 hour. The membranes were developed using SuperSignal West Atto (Thermo Scientific, A38554) and detected on UVP ChemStudio (Analytik Jena, 84997093001). Western blotting and gel data were analyzed using ‘Gels’ plugin of Fiji software and its standard drawing/measurement tools.

SUPPLEMENTARY RESULTS

Patients' clinical phenotype

Patient 1

This female patient is of Northern European origin and family history is unremarkable except for the mother's seizures during childhood and her one miscarriage. The patient was born at term (GA 38 weeks) to non-consanguineous parents via vaginal delivery, after labor was induced due to intrauterine growth retardation (IUGR) and a small placenta. The infant had Apgar scores of 9 at both 1 min and 5 min; she had short stature (43cm, <P5), low birth weight (1920 grams, <P3), congenital microcephaly (OFC 29cm, <P3), and mild hypertonia with adducted thumbs. On day 1 she suffered from neonatal seizures, with the EEG showing independent multifocal temporal sharp rhythmic theta waves (left more than right). Treatment with Phenobarbital resolved the seizures and the EEG improved, but still showed residual epileptiform discharges. She continued to have mild hypoxia requiring oxygen supplementation. MRI imaging at age 5 days revealed regions in both hemispheres with a simplified gyral pattern but normal MR spectroscopy in grey and white matter (Figure S1). Hearing and vision were normal. Imaging of kidney, hip and skeleton was unremarkable. Urine analyses revealed markedly elevated excretion of uracil and thymine levels in two separate samples. Subsequently, analysis of the DPD activity in fibroblasts confirmed the presence of a DPD deficiency. Beta-alanine therapy was advised, but not initiated according to parents' wishes. Tests for organic acidemias, urea cycle defects, aminoacidemia, mitochondrial disease and peroxisomal diseases were unrevealing. Neurological exam improved, but microcephaly and poor growth persisted; she passed away at the age of 18 months of respiratory failure from bronchiolitis complicated by apparent bacterial and fungal superinfection, not responsive to bronchial lavage, antibiotics and oscillator ventilation.

Patient 2

This male was born to healthy non-consanguineous parents of southern European descent as their first child. He was born after an uncomplicated full-term pregnancy and vaginal delivery with normal growth parameters. Family history was unremarkable except for epilepsy in the paternal grandfather. On day 2 of life the patient experienced central apneas and conjugated

hyperbilirubinemia (max on day 5: 5.1 mg/dl); both features remained unexplained with spontaneous resolution.

The patient showed early onset mild delays in all developmental domains. Walking without support was attained at age 18 months, but his gait remained clumsy with frequent falls. Expressive language development was impaired; at age 2.5 years, he only conveyed monosyllables. Receptive language skills appeared better but were not formally tested. Hearing and vision were normal. At age 2.5 years, a hyperkinetic movement disorder, attention deficit, and autistic traits were noted; he was affectionate in contact with mother. Also, there was failure to thrive with weight and height below the third centile; head circumference was just above the third centile. Around this time, he was diagnosed with a severe *Mycobacterium avis* pneumonia in the right upper lobe. Therapy consisted of prothionamide, eremfat, etambutol and clarithromycin. The start of treatment triggered chronic diarrhea, which persisted after termination of therapy. Biopsy at the age of 8 years confirmed an unspecific colitis. Additionally, the patient suffered from anemia, which was confirmed prior to the pneumonia and persisted despite iron supplementation. At age 6 years, cerebral MRI was normal. Dopamine (D2) receptor imaging (IBMZ-SPECT) identified only slightly reduced uptake in the left striatum compared to the right. Metabolic screen of beta-alanine and GABA was normal in cerebrospinal fluid.

At age 13 years, the patient was capable of some contact with peers, nevertheless he still exhibited behavioral problems. Word production was minimal. He rarely spoke sentences, while his receptive skills were remarkably good. He counted up to ten, read single letters and talked with 15 programmed icons incorporated in his talker device. Head circumference had normalized at the 10th centile, but weight and length remained below the third centile.

The patient suffered from recurrent pneumonias needing antibiotic treatment between ages 16 to 19 years. At this time, no evidence of immunodeficiency was found based on a normal count of granulocytes, monocytes and lymphocytes with a normal distribution of lymphocyte subpopulations. At age 18 years, he became weaker, could no longer climb stairs and was mostly dependent on a wheelchair. The patient became increasingly dyspneic. Ultimately, he experienced a septic cardiomyopathy, intracranial hemorrhages, a carnificating pneumonia, recurrent pulmonary hemorrhages/hematothoraces, and a coagulation disorder. After being weaned off extracorporeal membrane oxygenation for 3 months, the patient passed away at age 19 years.

Patient 3

This female patient was the first child of healthy first-cousin parents from Tunisian descent. Family history was unremarkable, with 2 younger healthy siblings. She was born via vaginal delivery after a full-term uncomplicated pregnancy. At birth, length, weight and head circumference were within normal limits (50.5 cm, 3840g, and 36cm, respectively). Congenital torticollis, broad nose, large ears, and hypertonia of hips adductors were noted on physical examination. The neonate was breast-fed during the first month, but due to failure to thrive and gastro-esophageal reflux, this was replaced by bottle feeding. At 3 months, the infant was not smiling, and eye contact was hard to obtain. Ophthalmologic examination was normal.

At 5 months, pediatric neurologic evaluation showed normal height and weight but microcephaly (-2SD), no head control or smile, axial hypotonia and peripheral hypertonia with hands shut. Right hip dysplasia was treated by a Pavlik harness. Brain MRI showed brain stem atrophy, dilatation of lateral ventricles, delayed myelination of the white matter. EEG showed a good sleep wake differentiation. Vision and hearing were normal. Metabolic testing showed abnormal uracil and thymine levels in urine, and subsequent enzymatic analysis confirmed DPD deficiency but no conclusive *DPYD* genotype was observed.

Aspiration pneumonia due to uncoordinated swallowing occurred; gastrostomy was performed at age 2.5 years. Despite antibiotic prophylaxis these pulmonary infections persisted requiring hospital admissions, oxygen supplementation and IV antibiotics. Physio-, ergo- and speech therapy were provided at a medical day care for children with disabilities. She developed progressive tetraparesis and scoliosis. She underwent two surgical procedures for bilateral hip dysplasia and was prescribed a brace. At the age of 3.5 years, bilateral breast buds and pubic hair were noted due to central precocious puberty, successfully treated with a GnRH analogue (triptoreline embonate). Brain MRI at age 4 years showed enlarged lateral and third ventricles, decreased volume of cerebral white matter and incomplete myelination, and pontocerebellar atrophy (Figure 1).

At the age of 6 years, she developed myoclonic epilepsy treated with valproate and clobazam resulting in partial seizure control. At age 12 years, bilateral cataracts were diagnosed; there was no eye contact, severe spasticity, short stature and microcephaly (both at -3,5 SD) and a normal weight. Pubertal stage was S3 P3. At 13 years, the child suffered respiratory insufficiency during a severe pneumonia, and passed away.

Genome sequencing

As it has been previously demonstrated that DPD deficiency may be due to a more complex mechanism of gene inactivation[3], we performed singleton genome sequencing (GS) on all three patients. Thorough, *DPYD*-centric analysis for more complex mechanisms of gene inactivation, including structural variants[3] mobile element insertions[53] repeat expansions[13] variants in non-coding regions[54] as well as mosaic variants[55] confirmed that there are no variants of significance in *DPYD*, which prompted genome-wide analysis of the nuclear and mitochondrial genomes in search of variants in genes other than *DPYD*. We first focused on single nucleotide variants (SNVs) and protein-coding regions as described previously[10,13] and uncovered variants of interest in genes important for Fe-S co-factor biogenesis; *CIAO1* in two probands and *MMS19* in one proband.

In total 86 genes were identified for which at least two of the three patients carried one variant. The *CIAO1* gene was the only gene which could readily explain the biochemical phenotype of the patients. With respect to potential other variants which could explain (part) of the clinical phenotype, patient 2 was heterozygous for an 84bp deep intronic deletion in *GABRB3*, a gene implicated in autosomal dominant early infantile epileptic encephalopathy. The deletion has not previously been observed in gnomAD, Database of Genomic Variants (DGV), or in-house. However, the deletion is deep intronic (deletion breakpoints are 16kbp from exon 2 and 21kbp from exon 3). Moreover, to date, the reported pathogenic variants in *GABRB3* are missense variants or deletions encompassing one or several exons. Following these observations, this small, deep intronic deletion is probably not impacting the protein and therefore is unlikely implicated in the proband phenotype.

Evidence that heterozygous variants in *CIAO1* occur in trans

Genome sequencing identified two variants NM_004804.2:c.193C>T p.(Arg65Trp) and NM_004804.2:c.577C>T p.(His193Tyr) in *CIAO1* in patient 1 and two variants NM_004804.2:c.193C>T p.(Arg65Trp) and NM_004804.2:c.552G>C p.(Trp184Cys) in *CIAO1* in patient 2, the presence of which was confirmed with Sanger sequencing. However, no parental gDNA was available to determine the mode of inheritance of these variants. Sequence analysis of 6 clones of *Escherichia coli* containing individual cDNA isolated from fibroblasts of patient 1 showed that 2 clones contained the NM_004804.2:c.193C>T p.(Arg65Trp) variant and 4 clones contained the NM_004804.2:c.577C>T p.(His193Tyr) variant in *CIAO1*. For patient 2, analysis of 6 clones of *Escherichia coli* showed that 4 clones contained the NM_004804.2:c.193C>T p.(Arg65Trp) variant and two clones contained the

NM_004804.2:c.552G>C p.(Trp184Cys) variant in *CIAO1*. Thus, all colonies contained only one of the 3 reported *CIAO1* variants per clone.

Analysis of the mutation sites in the CIAO1 crystal structure

CIAO1 shows the seven-bladed β-propeller domain architecture characteristic for WD40 domain proteins. Each of the seven ~43 amino acid long WD40 repeats forms four antiparallel β-strands, of which the first forms a propeller blade together with the other 3 strands of the preceding repeat. The name of the repeat is derived from the conserved signature WD dipeptide at its C-terminal end. [56] Additional well conserved motifs within each repeat are a glycine and a histidine at approximate positions 9 and 10, respectively, and an aspartate found ca. 6 residues upstream of the WD motif. All have a function in stabilizing the propeller fold by forming hydrogen-bonding networks or crucial hydrophobic interactions.[57]

CIAO1 is a major component of the cytosolic iron-sulfur cluster assembly (CIA) machinery that delivers Fe-S clusters to cytosolic and nuclear Fe-S proteins. These clusters are initially assembled on the scaffold protein ISCU1 by the multimeric ISC complex responsible for *de novo* biogenesis of Fe-S clusters.[58] From there, the clusters are transferred to the CIA complex by the HSC20 co-chaperone, which directly binds to its CIAO1 component. HSC20 is known to interact with proteins that contain lysine-tyrosine-arginine (LYR) motifs[59,60], and the sequence of CIAO1 does indeed contain one LYR (residues 176-178, herein called LYR2) as well as further three homologous motifs ($I^{87}W^{88}K^{89}$, $I^{220}W^{221}R^{222}$, $S^{240}W^{241}K^{242}$= LYR1, LYR3 and LYR4, respectively). Kim and coworkers showed that mutation of the canonical LYR motif is sufficient to abrogate binding of HSC20 to CIAO1, whereas the LYR3 motif is not involved in the interaction.[60]

It was suggested that the cytosolic iron-sulfur assembly component 3 (CIAO3, a.k.a. Iron-Only hydrogenase-like Protein, IOP1) may function in transferring iron-sulfur clusters from the initial assembly complex to CIAO1[61-63], either instead or in addition to HSC20. CIAO3 also binds directly to CIAO1, though the precise binding site has not yet been pinpointed.

We analyzed the point mutation sites in the crystal structure of CIAO1[64] to predict their putative effects on structure and function of the protein. R65 is located near the central pore of the β-propeller (Figure S5A). Its guanidinium group is solvent-exposed (Figure S5B) and creates, together with K66 and K109, a distinctly positively charged surface surrounding a small pocket at the center of the narrow-end face of the propeller (Figure S5C). WD40 domain proteins are scaffold proteins mediating protein-protein interactions, and it is therefore feasible to assume that the positively charged cavity and surface represent a binding site for interaction

with components or substrates of the CIA multi-subunit complex. The notion of a functional rather than structural role of R65 is supported by the fact that this residue is well conserved in CIAO1 and homologous proteins across species, but is not conserved in other members of the WD40 domain family. R65 is surrounded by K66, K109, and N307 on one side, and by the large aromatic side chains of F81, W20, and Y256 on the other (Figure S5D). Mutation of R65 to tryptophan would cause steric clashes with K66, W20 and F81. Structural rearrangements required to accommodate the bulkier tryptophan side chain are due to the surface location of the site likely of minor extent, as indicated by the only moderately lower stability of the p.Arg65Trp variant (Figure S4). Nevertheless, the mutation results in a significant decrease in positive electrostatic surface potential in and around the central cavity, which may be sufficient to abrogate interaction with proteins binding to this surface. A recently published crystal structure revealed that CIAO2B is the component of the CIA multi-subunit complex engaging with precisely this CIAO1 surface. The primarily electrostatic interaction involves insertion of a negatively charged glutamate (E133) into the CIAO1 central cavity and formation of salt bridges with R65, K66 and K109.[65]

In CIAO1, but not WD40 domain proteins, in general, a hydrophobic residue is found at or near the N-terminus of the first of the β-strands formed by each repeat. W184 represents this hydrophobic residue in repeat 5. Like R65, W184 is partially solvent exposed, and thus may be contacted by interacting proteins (Figure S5B). A likely candidate is HSC20, as W184 is found in intimate neighborhood to the LYR2 motif that was shown to be recognized by the co-chaperone (Figures S5A, B and E). It may contribute to the stabilization of the HSC20-binding site architecture by engaging in a water-mediated hydrogen bond with the side chain of E179 that directly follows the LYR motif in sequence, and, more importantly, by forming a hydrophobic cluster with the surrounding side chains of Y177 (from LYR2), L119, and W158. Mutation of W184 to cysteine creates an energetically unfavorable cavity in the otherwise densely packed hydrophobic cluster, which explains the observed structure-destabilizing effect of this variant (Figure S4). This in turn may negatively impact CIAO1's ability to interact with HSC20 and other proteins.

H193 is also located in the 5th repeat, in the longer loop that connects one propeller blade to another (Figure S5A). It represents the histidine that is highly conserved in all existing WD40 repeats and thus likely plays a crucial structural role. In the CIAO1 crystal structure, H193 is hydrogen-bonded to D215 and S211 via its side chain, and to a number of water molecules via its main chain, which in turn are linked to D172, L90 and G192 (Figure S5F). Residues of the canonical LYR2 (L176, Y177) and the homologous LYR3 motif are in close neighborhood but

not directly interacting with H193. Nevertheless, the hydrogen-bonding interactions of H193 link the two consecutive blades carrying these motifs stably together. Mutation of H193 to tyrosine would abolish the two-side chain-mediated hydrogen bonds and cause steric clashes with R219 and W221 preceding and belonging to LYR3, respectively, and with the β-strand carrying S211. More pronounced structural changes can therefore be expected in this protein region that partly overlaps with the CIAO2B binding site[65], explaining the observed reduced stability of the H193Y CIAO1 variant (Figure S4).

Analysis of the deletion site in the MMS19 structural model

To account for the amino acid sequence deviations to mouse MMS19, whose crystal structure was recently determined in complex with CIAO1 and CIAO2B[65], the *in silico* analysis of the effects of the E213 deletion on human MMS19 was performed using the structural model predicted by AlphaFold.[20,21] The client adaptor protein MMS19 consists of 23 tandem helix-turn-helix motifs (i.e. HEAT-like repeats) that are stacked on top of each other to form a so-called alpha solenoid or alpha horseshoe fold (Figure S6) with super-helical structure. E213 is solvent-accessibly located in the first turn of one of the repeating α-helices in the N-terminal part of MMS19. The effects of the deletion are most likely restricted to helix shortening by one residue, and a modest repositioning of the preceding 2-3 residues (Figure S6). Any intramolecular interactions of E213 can theoretically be replaced by equivalent interactions of E212 that moves to fill the resulting gap, whereas the space occupied by the fully solvent-exposed E212 in the wild-type protein is partially filled by V211 in the mutant variant. The resulting local decrease in protein surface polarity should have no direct effects on fold and stability of MMS19, which is in agreement with the experimental data.

In the crystal structure of the CIA targeting complex consisting of mouse MMS19 and fruit fly CIAO1 and CIAO2B (PDB-Id 6TC0[65]), the latter is located centrally, bridging the other two proteins (Figure S6). CIAO2B binds distant from E213 to the last HEAT repeat at the C-terminal end of MMS19, which is in agreement with our finding that the interaction with the other two constituents of the core CIA complex is not hampered by its deletion. The mutant variant's inability to mediate Fe-S cluster incorporation must thus have other reasons. Interestingly, E213 is part of a highly conserved surface area of MMS19 that was identified as potential client protein binding site by Kassube & Thomä.[65] Furthermore, the E213 site is also buried upon dimerization of the core CIA targeting complex (Figure S6). This dimeric form, observed in crystals and coexisting with monomeric forms in solution, has been implicated in

Fe-S cluster biogenesis though its precise role remains to be elucidated.[65] For both, interaction with client proteins and homo-dimerization, the change in interaction surface shape and polarity resulting from the deletion of E213 may have detrimental effects, which could explain the non-functionality of the MMS19-deletion mutant in Fe-S cluster incorporation.

Metabolomics and network analysis

Network analysis was performed to visualize the quantified metabolites and their biochemical relation in a holistic fashion. Specifically, the metabolite names were mapped onto a curated version of the HMR 2 genome-scale metabolic model[36] and calculated which metabolite pairs were ≤ 2 reaction steps apart in the model. This resulted in a network of connected metabolites that we integrated with biochemical reaction and pathway knowledge into an interactive HTML/JavaScript document, which can be downloaded from: https://labgmd.github.io/HRN_browser/CIAO1_MMS19

The results provide insight into where metabolites with altered levels in the patients are located in the network with respect to biochemical conversions that are catalyzed by enzymes containing an iron-sulfur cluster (Figure S11). As expected, this network visualization explains the buildup of uracil due to the presence of dihydropyrimidine dehydrogenase in the degradation pathway of uracil to beta-alanine. The increased concentration of 1-methylnicotinamide could be explained by a reduced activity of the iron-sulfur containing enzyme aldehyde oxidase 1 (AOX1) catalyzing the conversion of 1-methylnicotinamide to N1-methyl-2-pyridone-5-carboxamide. Since our metabolomics platform did not contain any of the intermediates of thymine degradation through dihydropyrimidine dehydrogenase, the corresponding pathway is not shown in the network.

***In-situ* flux analysis of CIAO1 and MMS19 deficiency**

Functional analyses of the citric-acid cycle and methionine-cysteine pathway, using stable-isotope-labeled metabolites for *in-situ* analysis of CIAO1 and MMS19 deficiency showed a decreased flux of isotope-labeled glutamine into the citric acid cycle in *CIAO1*-deficient fibroblasts, which was compensated by an increased flux from stable isotope-labeled oleate (Figures S12 and S13). In addition, a profoundly altered flux of methionine (Figure S14) towards cysteine was observed in fibroblasts of CIAO1 and MMS19 patients.

Lipidomics

A semi-targeted analysis of 2047 lipid species showed that the lipidome of CIAO1- and MMS19-deficient fibroblasts was significantly different from that observed in control fibroblasts (Figure 4). A strong increase in specific phosphatidylcholines and ceramide species was observed in both CIAO1 and MMS19-deficient fibroblasts (Figure 4). The highest fold-changes were found for phosphatidylcholine species with 40-50 carbon atoms in the combined fatty acid side chains containing a total of 4-11 of double bonds. These accumulating phosphatidylcholine species thus contain very long-chain (each 20-25 in length), polyunsaturated (each 2-6 double bonds) fatty acids. Ceramides showed a similar profile where species with 40-45 carbon atoms in the sphingoid backbone plus N-acylated fatty acid side chain with 3 or more double bonds (Fig. 4). As ceramides in fibroblasts primarily have a C18 sphingoid base this means that the N-acylated fatty acids in the accumulating species also are very long-chain fatty acids (C22-C27) and contain 2-4 double bonds. A similar phenomenon was also observed for other lipid species such as triacylglycerols (Figure S15).

Cellular Phenotype

The CIA complex is involved in the maturation of iron-sulfur cluster containing enzymes involved in the maintenance of genomic integrity[66-68]. A deficiency of XPD, a client protein of MMS19, has been linked to an increased radio-sensitivity [69]. Ionizing radiation-induced foci of DNA repair-related protein accumulation at DNA double-strand break sites and histone H2AX phosphorylation, resulting in γ-H2AX, is the first step in recruiting and localizing DNA repair proteins. Fibroblasts of patient 3, containing a mutant MMS19 protein, showed a decreased rate of DNA double-strand break repair and an increased number of γ-H2AX foci, 5 hours after irradiation (Figure S17).

Electron microscopy showed abundant lysosomes for all three patients and control fibroblasts. In addition to the presence of normal multilamelar lysosomes, dense lysosomal structures were detected and both were immuno-gold labelled for lysosomal marker CD63. These lysosomes were neither multi-vesicular nor multi-lamellar, but rather filled with an electron-dense content (Figure S18). Neither immunofluorescence nor electron microscopy showed evident differences for the cellular compartments and organelles, including lysosomes, lipid droplets, Golgi stacks, endoplasmic reticulum, mitochondria and early endosomes, between patients and control fibroblasts (Figure S19-22).

Ciao1 and Mms19 are essential for survival in zebrafish

To model the consequences of CIAO1 deficiencies, a zebrafish line carrying a 26-bp deletion (Figure S23B) targeting exon 3 (Figure S23A) in the *ciao1* gene was generated and confirmed (Figure S24). For *mms19*, two zebrafish lines were generated targeting exon 5 (Figure S25A); one with 5-bp deletion (Δ5) and one with 14-bp insertion (ins14) (Figure S25B-D). All variants were predicted to result in frameshifts and protein truncations (Figures S23C and S25F). In fact, for the *mms19*, the assessment of the expression levels using the reporter vector, P2A-sfGFP cassette[46] (Figures S25E) revealed that while the wild-type constructs produced fluorescent cell labeling in 83 % of the injected embryos, the mutants [(Δ5) and (ins14)] were unable to produce any expression confirming the efficient frameshifts by both variants (Figures S25E).

Phenotypic analysis of the *mms19* mutants supported the essential role of the Mms19 in zebrafish (Figures S28A). Namely, when we genotyped progeny from *mms19* mutant heterozygote crosses at 3 dpf, we observed expected genotype frequencies; however, by 3 months none of the homozygote mutant fish could be identified suggesting that mutant fish cannot survive to adulthood without functional Mms19 (Figure S28A). Next, we performed larval genotyping by fin clipping at 3 dpf to isolate larvae of defined genotypes (Figure S28B) and analyzed the survival and phenotypes of wild-type and *mms19*$^{ins14/ins14}$ larvae for approximately one month.

Given that the CIA pathway is typically essential for normal cell function and embryonic development in the mouse[70] such a long persistence of *mms19*$^{ins14/ins14}$ juvenile zebrafish can be partially explained by the maternal contributions of the Mms19 mRNA that we identified by plotting the publicly available transcriptomic data[71] for *ciao1* and *mms19* (Figure S29) or by Mms19-independent Fe-S cluster insertion albeit likely at a lower efficiency.

Loss of *mms19* and *ciao1* leads to a reduction in the level of DPD proteins

To examine the effects of *mms19* loss, we genotyped larvae and collected protein samples from 4 wild-type and 5 mutant larvae pools at 6 and 26 dpf stages. We then performed Western blotting of these samples using antibodies against human DPD, POLD1 (both are known Fe-S proteins) and β-Actin proteins predicted to cross-react in zebrafish. Zebrafish have 4 *DPYD* gene homologs, 3 of which do not have known expression data, whereas the *pold1* gene is well-studied in zebrafish with a known mutant[72] and expression pattern biased toward proliferating

cells. All three of these antibodies worked (Figure S26A), but only in the case of DPD proteins were significant differences in levels observed at both developmental stages (Figure S26B), however these differences were more pronounced at 26 dpf consistent with greatly increased mutant mortality at this stage. At 26 dpf, there was also a decrease in one of the Pold1 bands potentially connected to the lack of Fe-S cluster insertion. These results indicate that Mms19 is functionally conserved in zebrafish.

Since $ciao1^{del26/del26}$ mutant zebrafish survive to about 15 days of development, we analyzed their DPD and Pold1 levels at 7 dpf (Figure S27) in the same manner as described above. Levels of DPD were nearly undetectable in $ciao1^{del26/del26}$ mutant samples (n=4) compared to wild-type samples (n=4) which showed robust bands, whereas Pold1 and β-Actin were expressed at comparable levels in samples of both genotypes (Figure S27A). Samples from two independent experiments were quantified and analyzed for statistical significance, which revealed that DPD downregulation in mutant samples was highly significant, whereas Pold1 expression was not significantly different (Figure S27B). This result suggests that the role of Ciao1 in Fe-S insertion is essential and occurs early in larval development.

Human Disorders and cofactor deficiency

Human disorders are known to be associated with a disrupted metabolism of the following cofactors: tetrahydrobiopterin (n=6), thiamine pyrophosphate (n=4), flavin mononucleotide and flavin adenine dinucleotide (n=5), nicotinamide adenine dinucleotide (n=6), coenzyme A (n=4), pyridoxal 5’ phosphate (n=6), adenosyl- and methyl-cobalamin (n=19), tetrahydrofolate (n=9), biotin (n=3), molybdenum cofactor (n=5), glutathione (n=8), menaquinone (n=3), ubiquinone (coenzyme Q, n=11), and lipoic acid and mitochondrial iron-sulfur cluster metabolism (n=16).[73,74]

SUPPLEMENTARY FIGURES

Figure S1

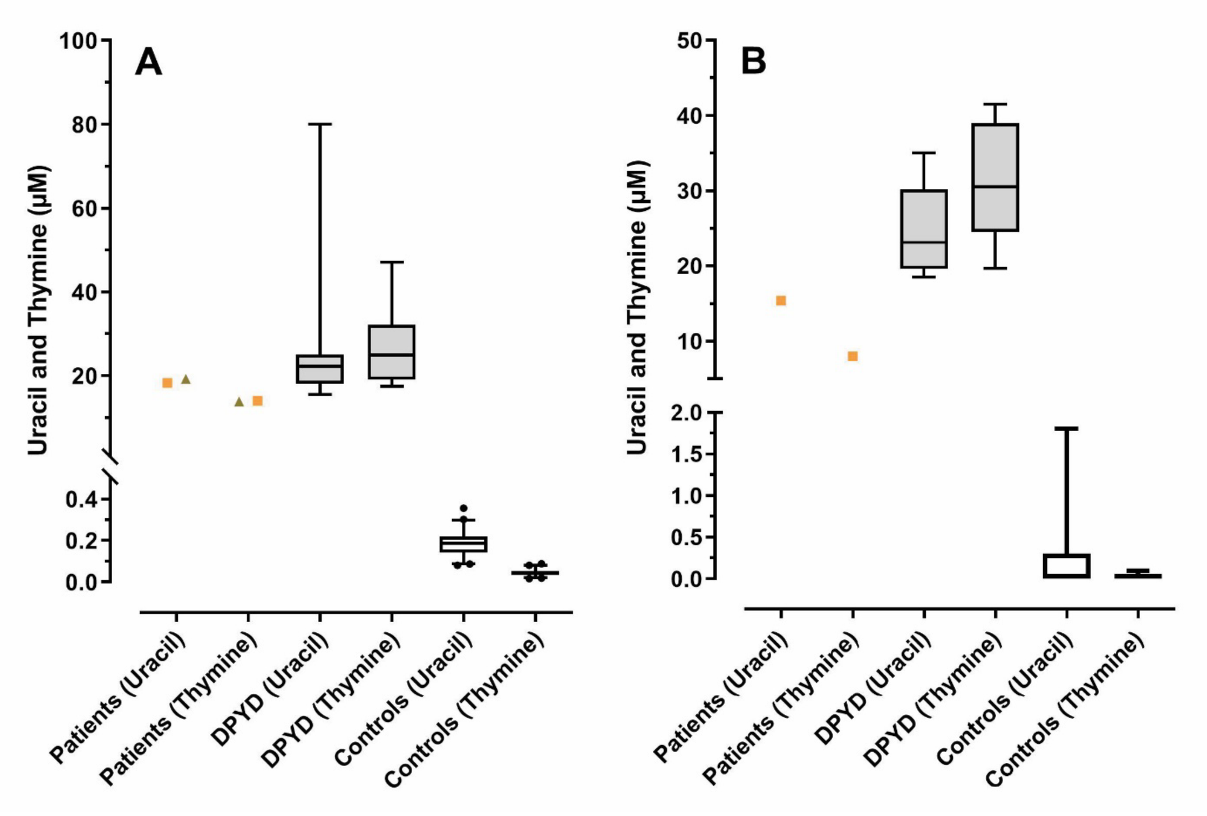

Figure S1. Concentrations of uracil and thymine in *CIAO1* and *MMS19* deficient patients. Panel A shows the plasma levels of uracil and thymine in patient 2 (■), patient 3 (▲), *DPYD* deficient patients (n=15) and controls (n=100). Panel B shows the levels of uracil and thymine in cerebrospinal fluid of patient 2 (■), *DPYD* deficient patients (n=7) and controls (n=32). The top, bottom and line through the middle of a box correspond to the 75th percentile, 25th percentile and 50th percentile, respectively. The whiskers on the bottom extend from the 2.5th percentile and top 97.5th percentile.

Figure S2

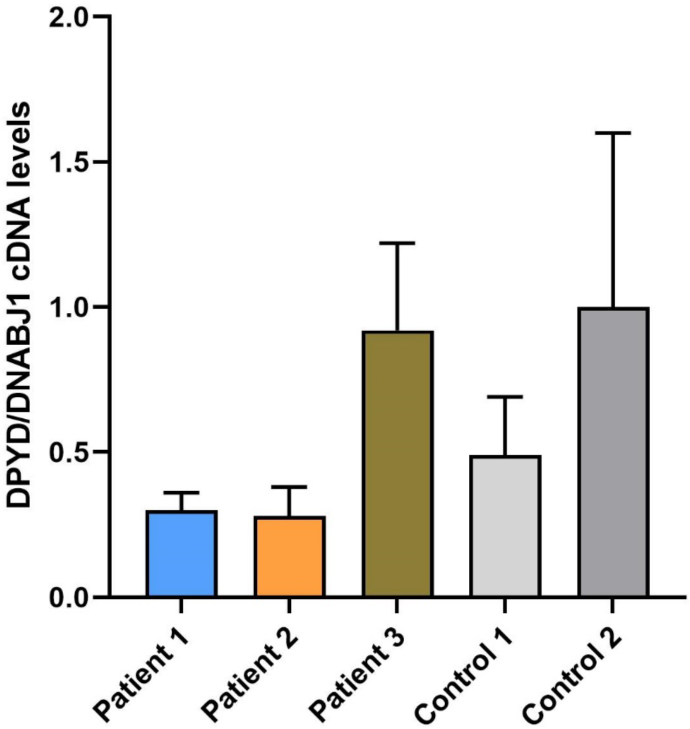

Figure S2. Quantitative PCR analysis of *DPYD* cDNA and the reference gene DnaJ heat shock protein family (Hsp40) member B1 (DNAJB1). The results are expressed as mean + SD (n=3).

Figure S3

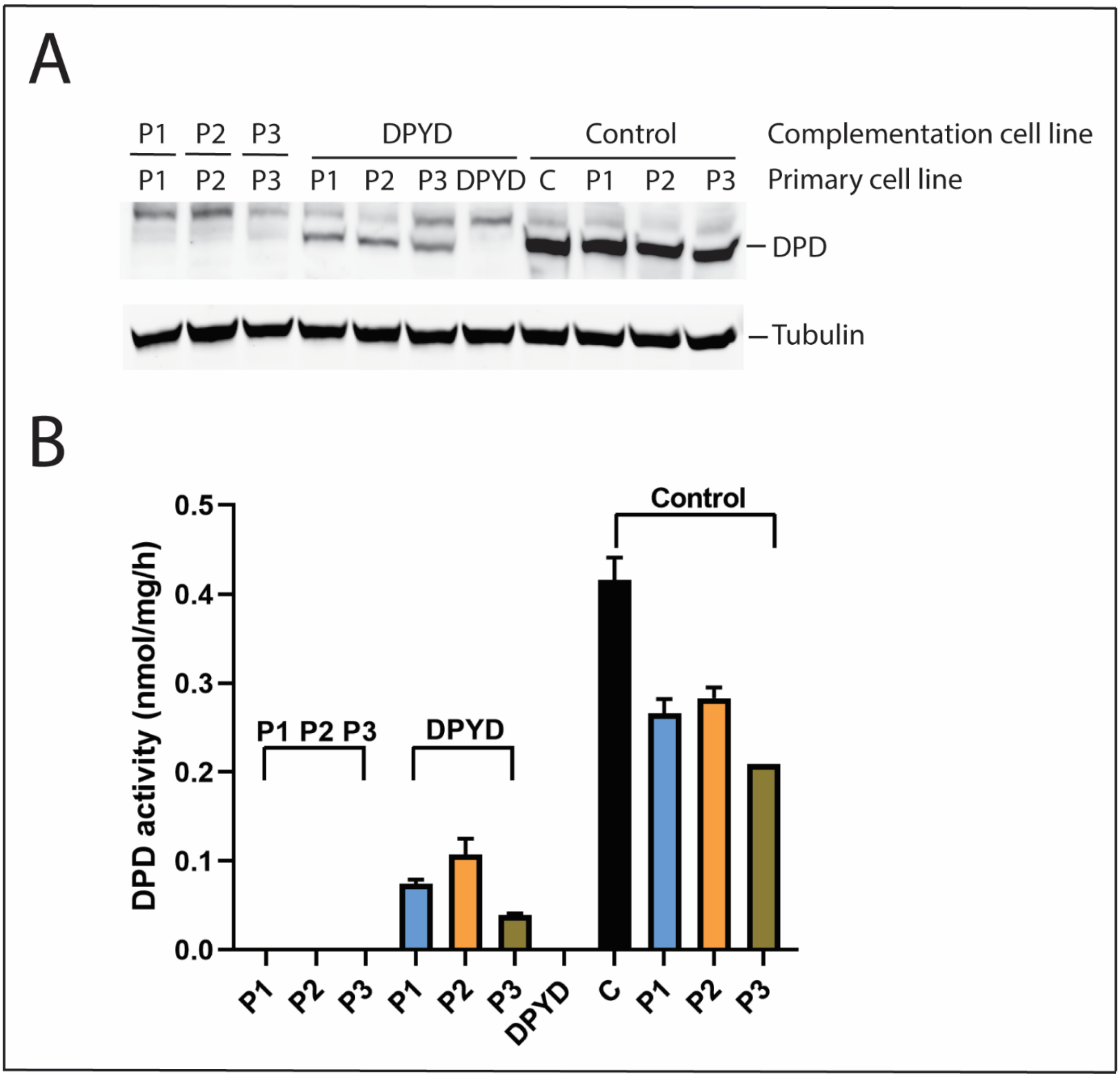

Figure S3 Complementation analysis of patients' fibroblasts. Fibroblasts from the patients were fused with fibroblasts from the indicated patient, a healthy volunteer (control) and a *DPYD*-deficient patient, due to homozygosity for the deleterious NM_000110.4:c.299_302del variant in *DPYD*. Panel A shows the DPD protein expression and Panel B shows the DPD activity after complementation of the fibroblasts.

Figure S4

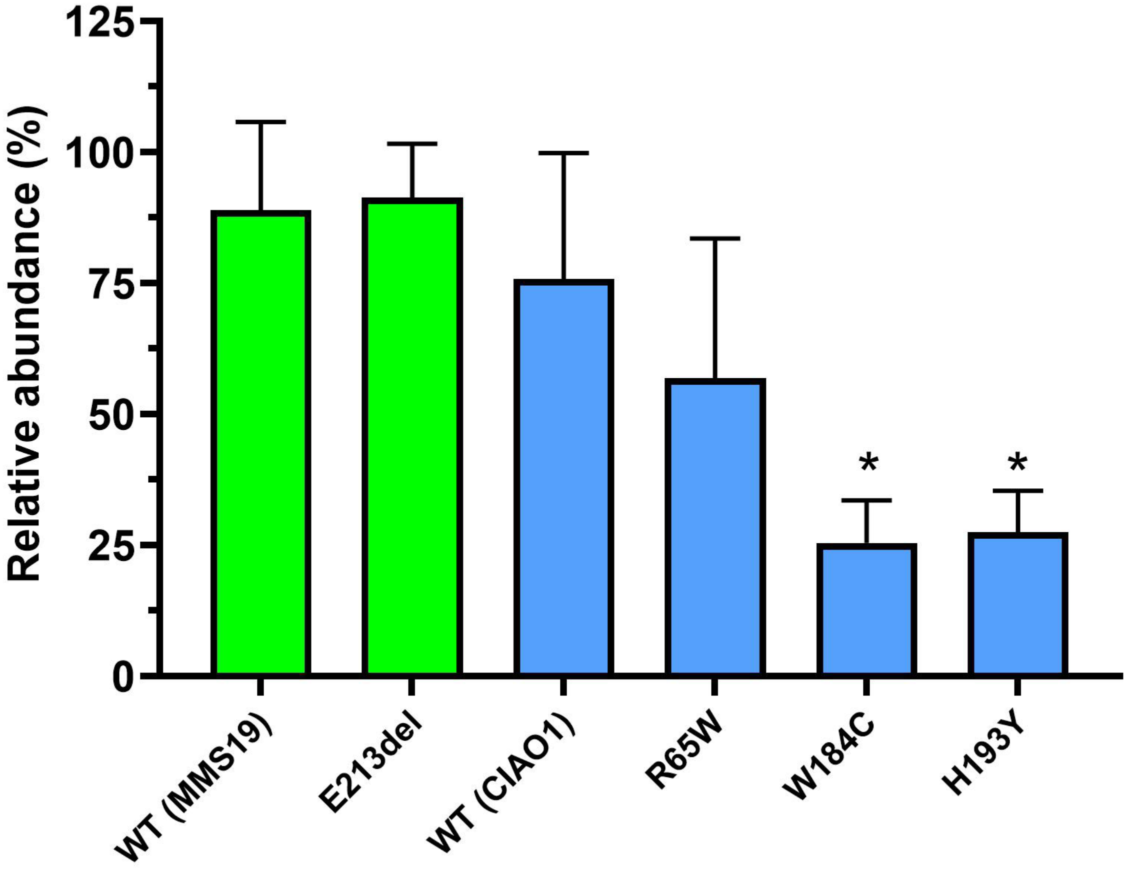

Figure S4. **Relative stability of MMS19 and CIAO1 mutant proteins.** The amount of recombinant wild-type and mutant MMS proteins (Green) and recombinant wild-type and mutant CIAO1 proteins (Blue) was determined after culturing transfected HEK293T cells in the absence and presence of cycloheximide. The results are expressed as mean ± SD (n=4). *indicates a significant difference (P=0.02) between the abundance of the mutant CIAO1 protein compared to the wild-type CIAO1 protein.

Figure S5

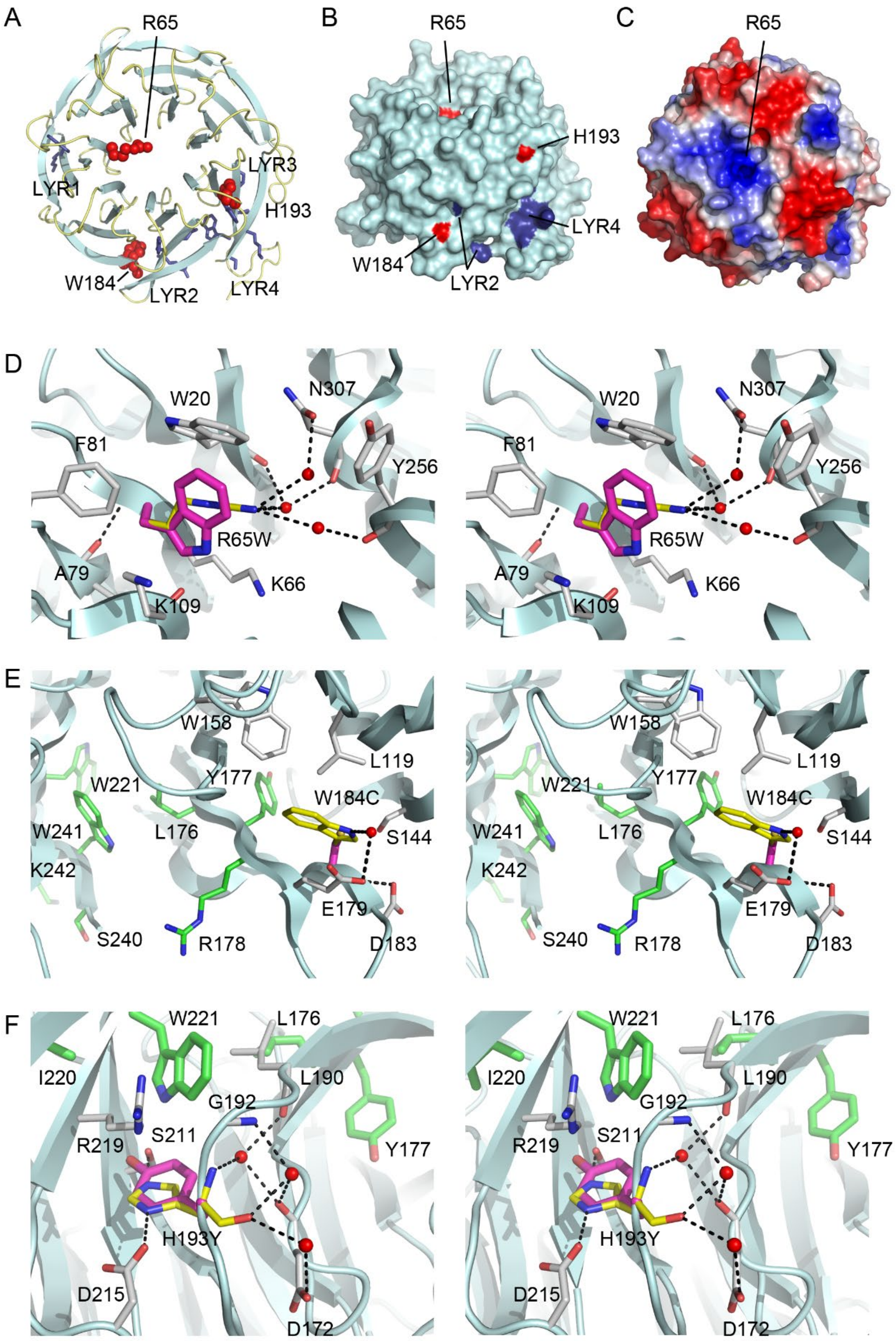
A
R65
LYR1
LYR3
H193
W184
LYR2
LYR4
B
R65
H193
LYR4
W184
LYR2
C
R65
D
W20
N307
F81
Y256
R65W
A79
K66
K109
W20
N307
F81
Y256
R65W
A79
K66
K109
E
W158
L119
W221
Y177
W184C
W241
L176
S144
K242
E179
S240
R178
D183
W158
L119
W221
Y177
W184C
W241
L176
S144
K242
E179
S240
R178
D183
F
W221
L176
I220
L190
G192
S211
R219
Y177
H193Y
D215
D172
W221
L176
I220
L190
G192
S211
R219
Y177
H193Y
D215
D172

Figure S5. The crystal structure of CIAO1 and localization of the mutated residues. (A) Schematic view of CIAO1 in cartoon representation with β-strands and loops coloured pale-blue and –yellow, respectively. The residues affected by the reported point mutations are shown as space fill models in red. Residues of the canonical (LYR2) and homologous LYR motifs (LYR1,3,4) are shown as sticks in blue. (B) Surface representation of CIAO1, with mutations sites coloured red and LYR motifs dark blue. The view differs from that shown in (A) and (C). (C) Surface representation of CIAO1 coloured according to the electrostatic surface potential (red: negative, blue: positive) as calculated by PyMOL. The location of R65 at the bottom surface of the propeller domain is indicated. Stereo views of the point mutation sites R65W, W184C and H193Y and their environment are shown in panels D, E and F, respectively. CIAO1 is shown in cartoon representation in pale-blue as in (A). Stick models of the side chains introduced by the point mutations are shown with carbon atoms in magenta as the most preferred rotamer causing the least clashes with surrounding residues. The natively occurring side chains at these sites are shown with carbon atoms in yellow. Residues of the LYR1-4 motifs are shown as sticks with carbon atoms in green, all other residues with carbon atoms in white. Nitrogen, oxygen and sulphur atoms are shown in blue, red, and yellow-orange, respectively. Hydrogen bonds are indicated by dotted black lines, water molecules as red spheres.

Figure S6

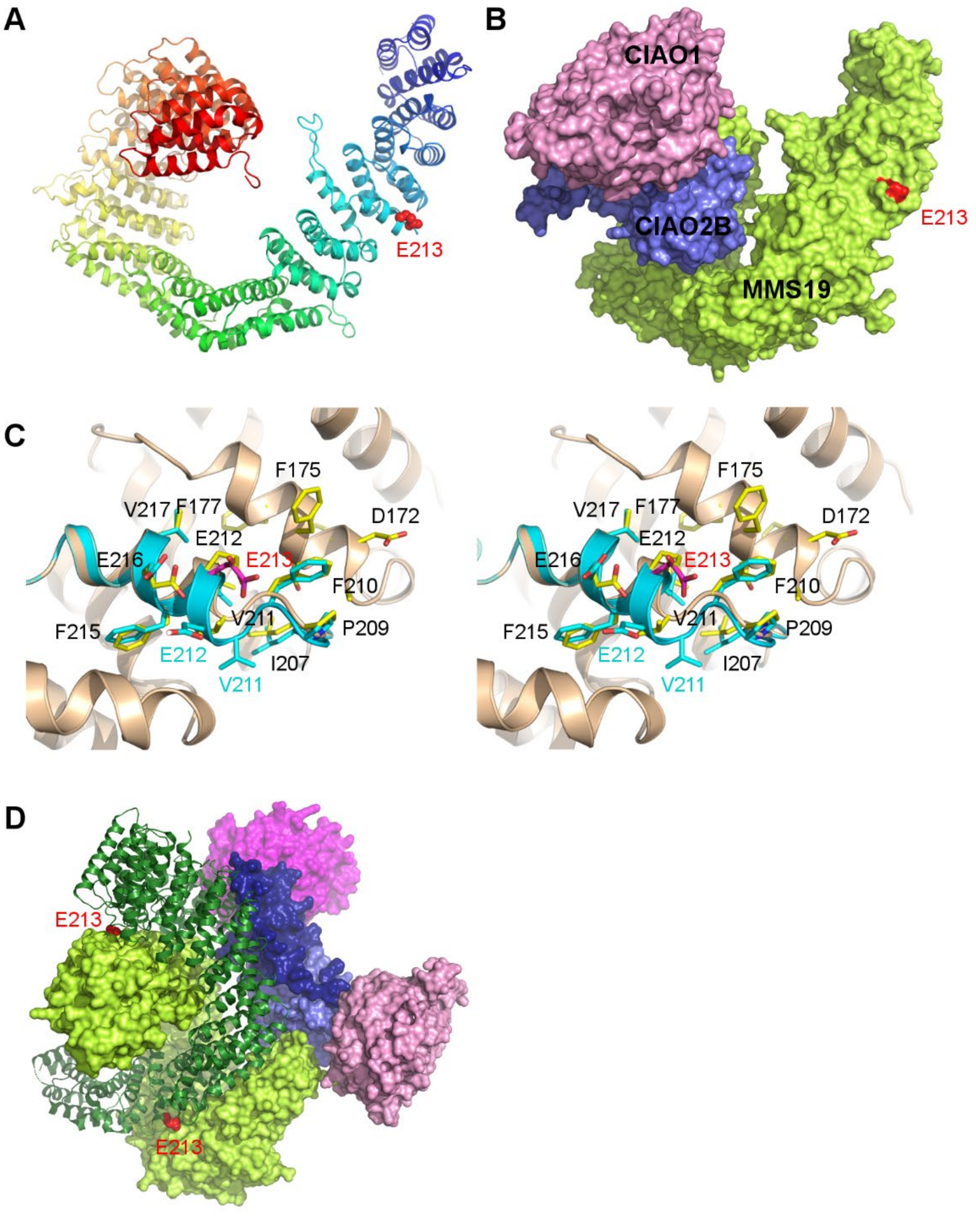

Figure S6. Localization of the E213 deletion site in MMS19. (**A**) Cartoon representation of the human MMS19 structure as predicted by AlphaFold.[20,21] The molecule is coloured as rainbow starting with blue at the N-terminus to red at the C-terminus. The deletion site is highlighted by the ball-and-stick representation of the E213 side chain. (B) Surface representation of the CIA targeting complex, consisting of MMS19 (yellowgreen), CIAO1 (pink), and CIAO2B (blue). The complex was generated from the PDB-entry 6TC0[65], in which the crystal structure of mouse MMS19 was replaced by the AlphaFold-model of the

corresponding human protein. The location of E213 is marked in red. (**C**) Stereo view of the E213 deletion site. Manual deletion of E213 from the AlphaFold-derived model, followed by structure regularization in WinCoot, resulted in the structure shown as cartoon in beige. This structure differs from the superimposed original model only in the parts shown in cyan. The deletion site is highlighted by the side chain of E213 depicted with carbon atoms in magenta. The side chains of neighbouring residues are shown as sticks with carbon atoms in yellow (deletion mutant) and cyan (wild-type), respectively. The latter are labelled only if their location differs significantly from that of the corresponding residue in the deletion mutant model. (D) The dimeric form of the CIA core complex consisting of mouse MMS19 and fruit fly CIAO1 and CIAO2B as observed by Kassube & Thomä (6TC0)[65]. One half of the complex is represented and coloured as in (B). In the second copy of the CIA, mouse MMS19 is shown as cartoon in dark green, and CIAO1 and CIAO2 in surface representation in magenta and dark blue, respectively. The location of E213 (highlighted in red) coincides with a MMS19 surface buried upon dimerization.

Figure S7

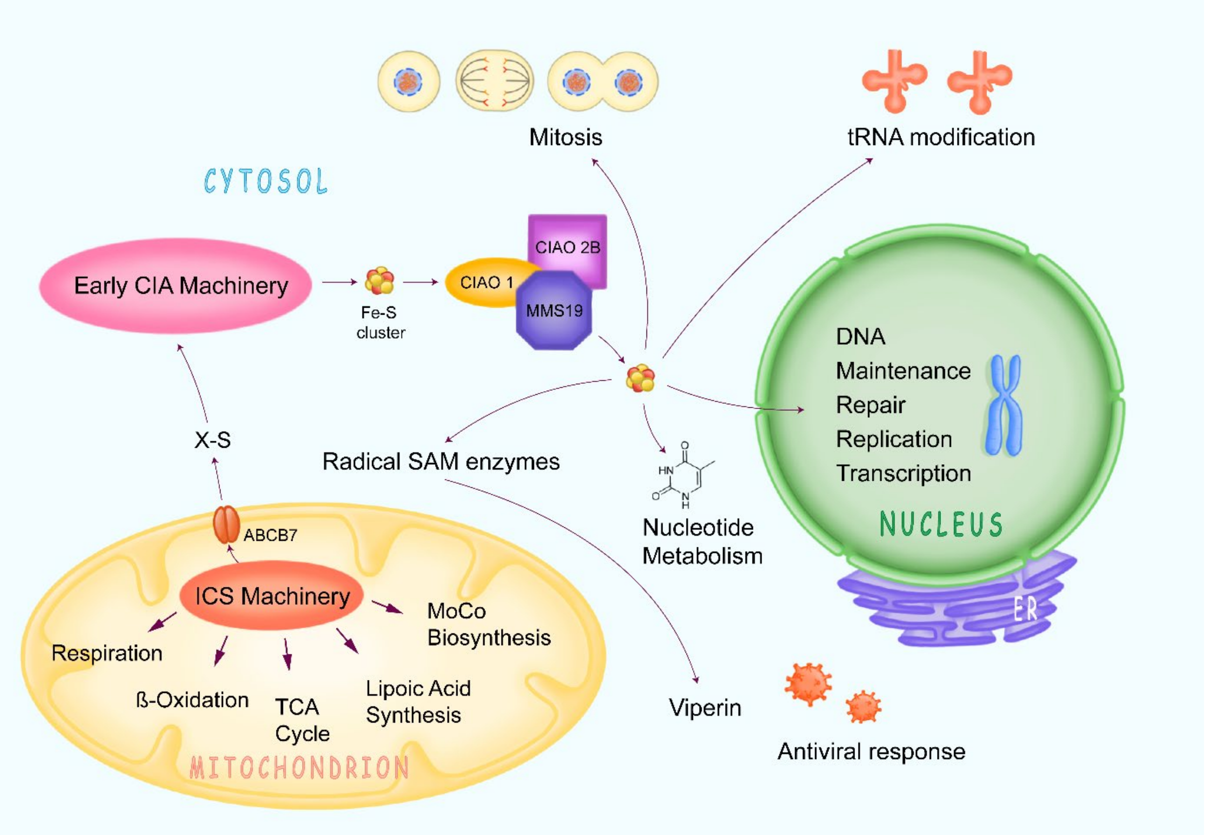

Figure S7. Biogenesis of iron-sulfur proteins. Mitochondrial iron-sulfur proteins are generated by the ISC assembly machinery. The iron-sulfur cluster dependent processes in mitochondria include mitochondrial respiration, the ETFDH-dependent ß-oxidation of fatty acids, the tricarboxylic acid (TCA) cycle, lipoic acid and molybdenum cofactor (MoCo) synthesis. A sulfur-containing factor X-S is exported via the mitochondrial ABC transporter ABCB7 to the cytosol and used by the CIA machinery to generate both cytosolic and nuclear iron-sulfur proteins. These proteins are involved in numerous anabolic and catabolic reactions, protein translation, mitosis, tRNA base modification, the complex process of genome maintenance and antiviral defense. ER, Endoplasmic Reticulum.

Figure S8

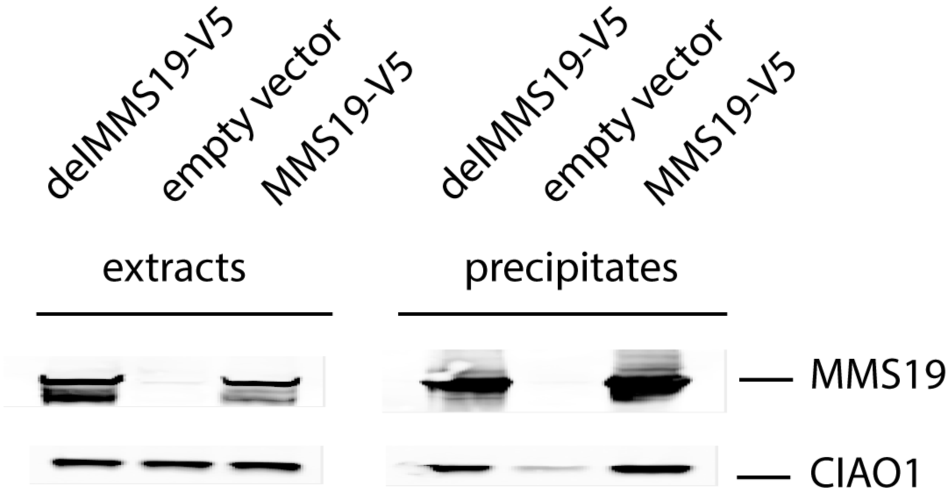

Figure S8. Co-immunoprecipitation of MMS19 and CIAO1. The left-hand panel shows the recombinant expression of wild-type MMS19 (MMS19-V5) and mutant MMS19 (delMMS19-V5) as well as the endogenous levels of CIAO1 in HEK293 cells. The right-hand panel shows the co-immune precipitation of wild-type (MMS19-V5) and mutant MMS19 (delMMS19-V5) proteins with bound CIAO1 protein, in HEK293 cells, after immunoprecipitation using V5-agarose.

Figure S9

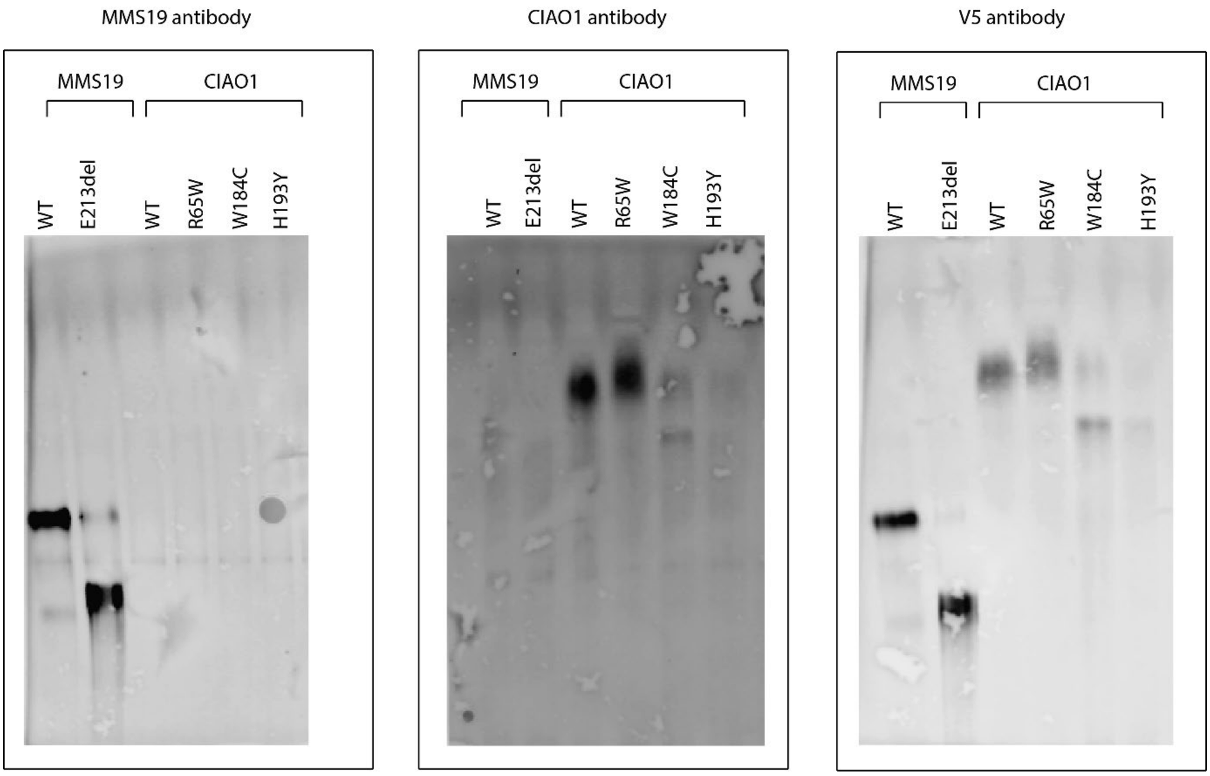

Figure S9. Native gel of transiently expressed wild-type and mutant MMS19 and CIAO1. *MMS19* (wild-type and mutant) and *CIAO1* (wild-type and mutant) cDNA were transiently expressed in HEK293T cells followed by native gel electrophoresis and immunoblotting with a monoclonal antibody against MMS19, CIAO1 and a V5 tag. Recombinant expression of wild-type MMS19 resulted in a single distinct protein band whereas the expression of mutant MMS19 was associated with a lower molecular weight protein complex. The recombinant expression of wild-type CIAO1 and mutant R65W CIAO1 resulted in the formation of a large protein complex with a comparable molecular weight. In contrast, only a low amount of a normal sized and smaller sized protein complex was observed for the recombinant CIAO1 carrying the W184C or H193Y variants.

Figure S10

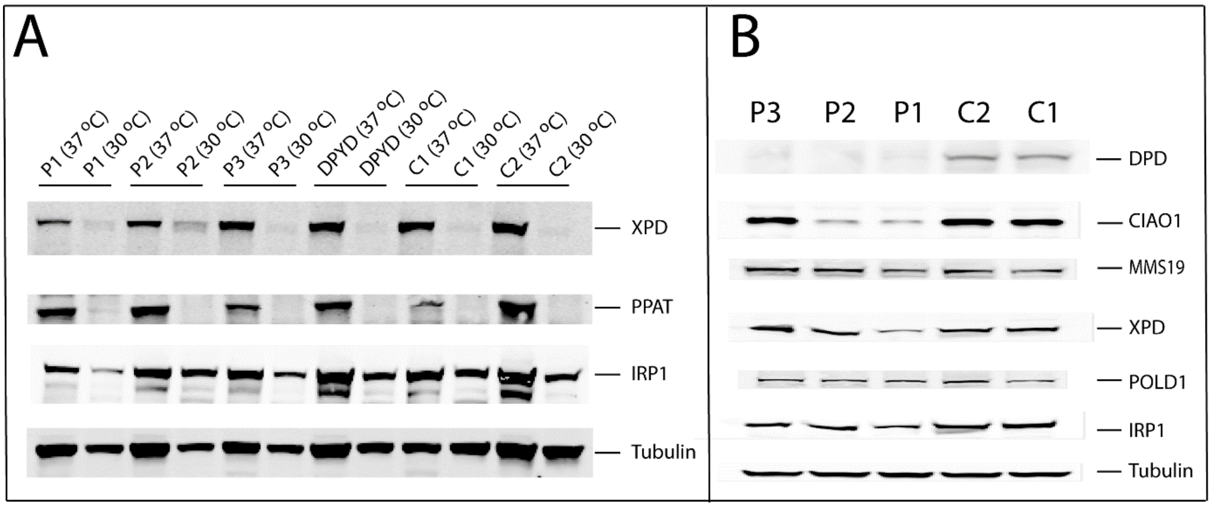

Figure S10. Client proteins of CIAO1 and MMS19. Panel A shows the protein expression of the client proteins XPD and PPAT in confluent fibroblasts at 30ºC and 37ºC. Panel B shows the protein expression of DPD, CIAO1, MMS19 and the client proteins XPD and POLD1 in exponentially growing fibroblasts. As a control, the expression of the iron-sulfur containing protein IRP1, a non-client protein of CIAO1 and MMS19, is shown.

Figure S11

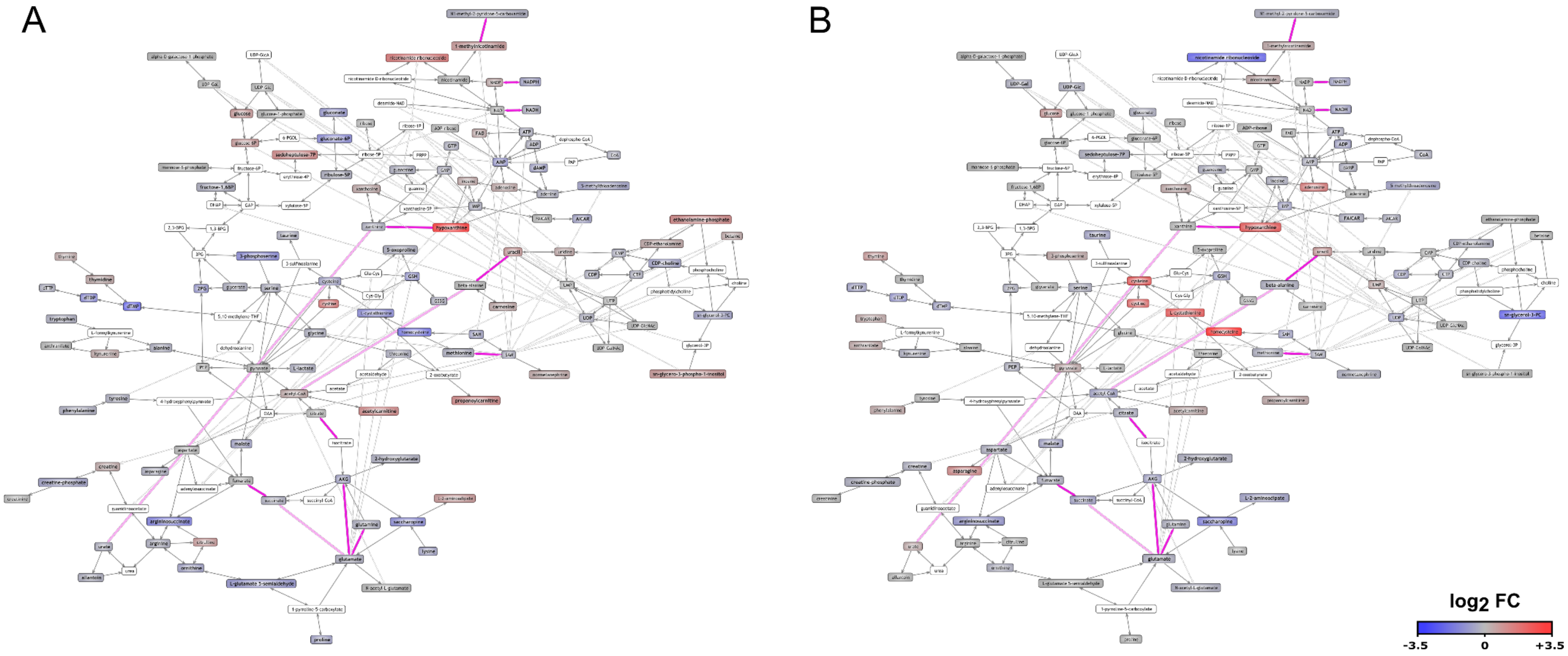

Figure S11. **Network analysis results of the metabolomics data of patients with the CIAO1 (A) and MMS19 (B) variant versus healthy control.** Metabolites (nodes) are colored according to their effect size, where red indicates a positive log fold change of the patient data with respect to control and blue a negative log fold change. Reaction paths (edges) have been highlighted in violet when they contain a reaction step catalyzed by a human enzyme containing an iron-sulfur cluster according to Lill and Freibert [40]. A high resolution interactive figure can be retrieved from https://labgmd.github.io/HRN_browser/CIAO1_MMS19.

Figure S12

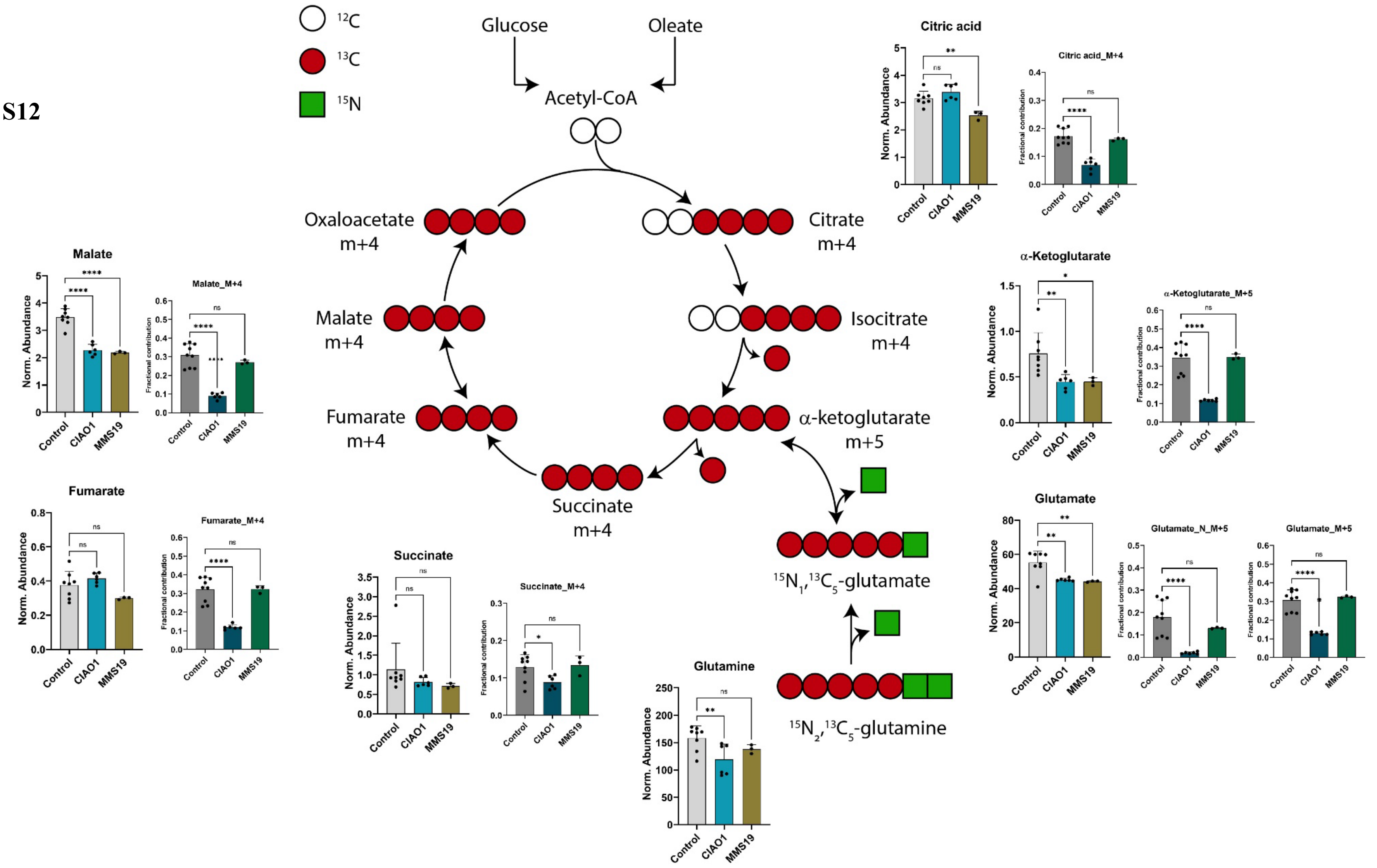

Figure S12. Metabolomics and flux analysis of stable-isotope labeled glutamine. Mass isotopomer distributions of metabolites of the citric acid cycle after introduction of fibroblasts of the CIAO1 and MMS19 patients and controls with $^{15}N_2$,$^{13}C_5$-glutamine. The mass isotopomer distributions were corrected for natural isotope abundances using mass isotopomer distribution analysis.

Figure S13

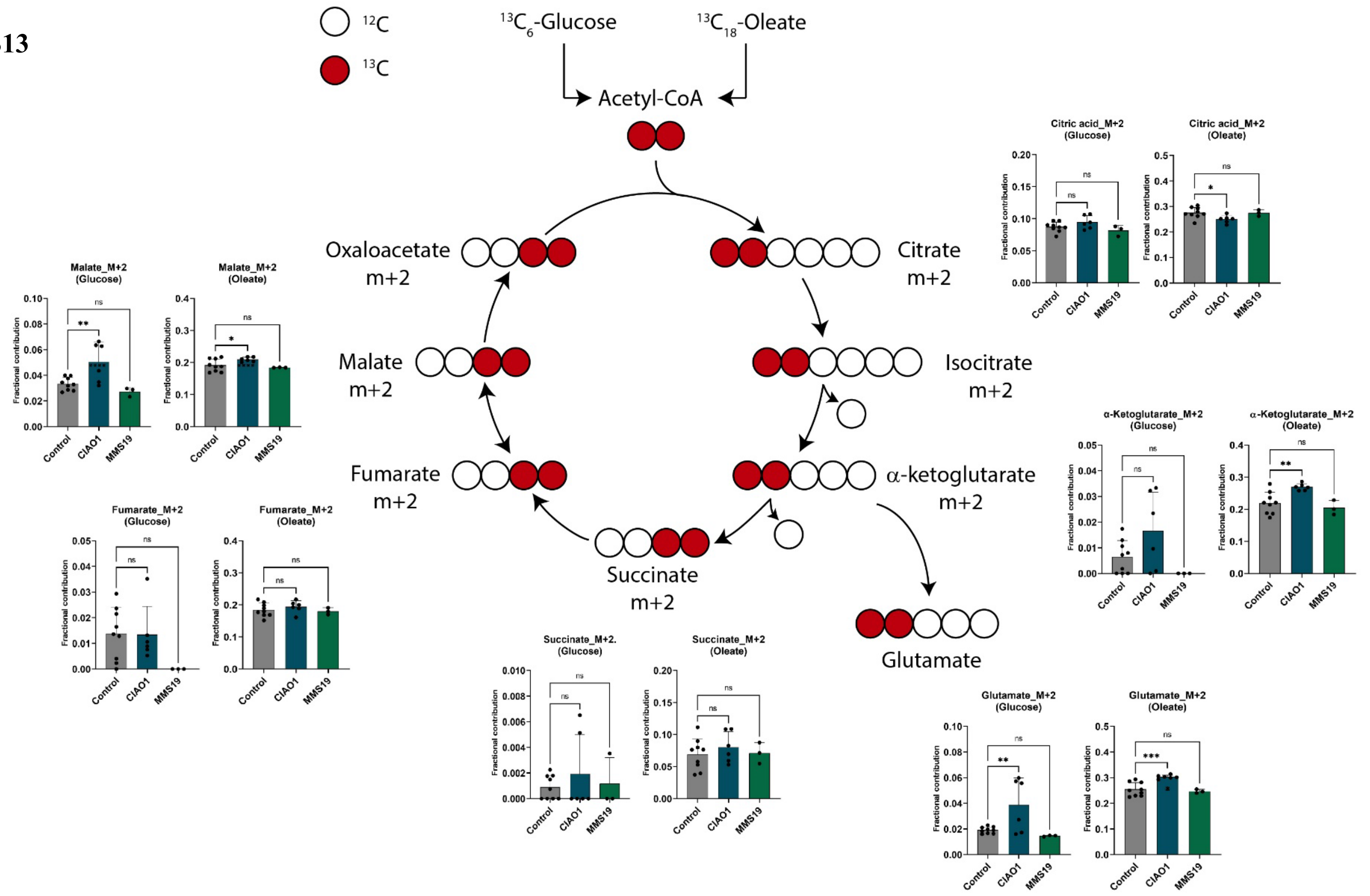

Figure S13. Flux analysis of stable-isotope labeled glucose and oleic acid. Mass isotopomer distributions of metabolites of the citric acid cycle after introduction of fibroblasts of the CIAO1 and MMS19 patients and controls with $^{13}C_6$-glucose and oleic acid or $^{13}C_{18}$-oleic acid and glucose. The mass isotopomer distributions were corrected for natural isotope abundances using mass isotopomer distribution analysis.

Figure S14

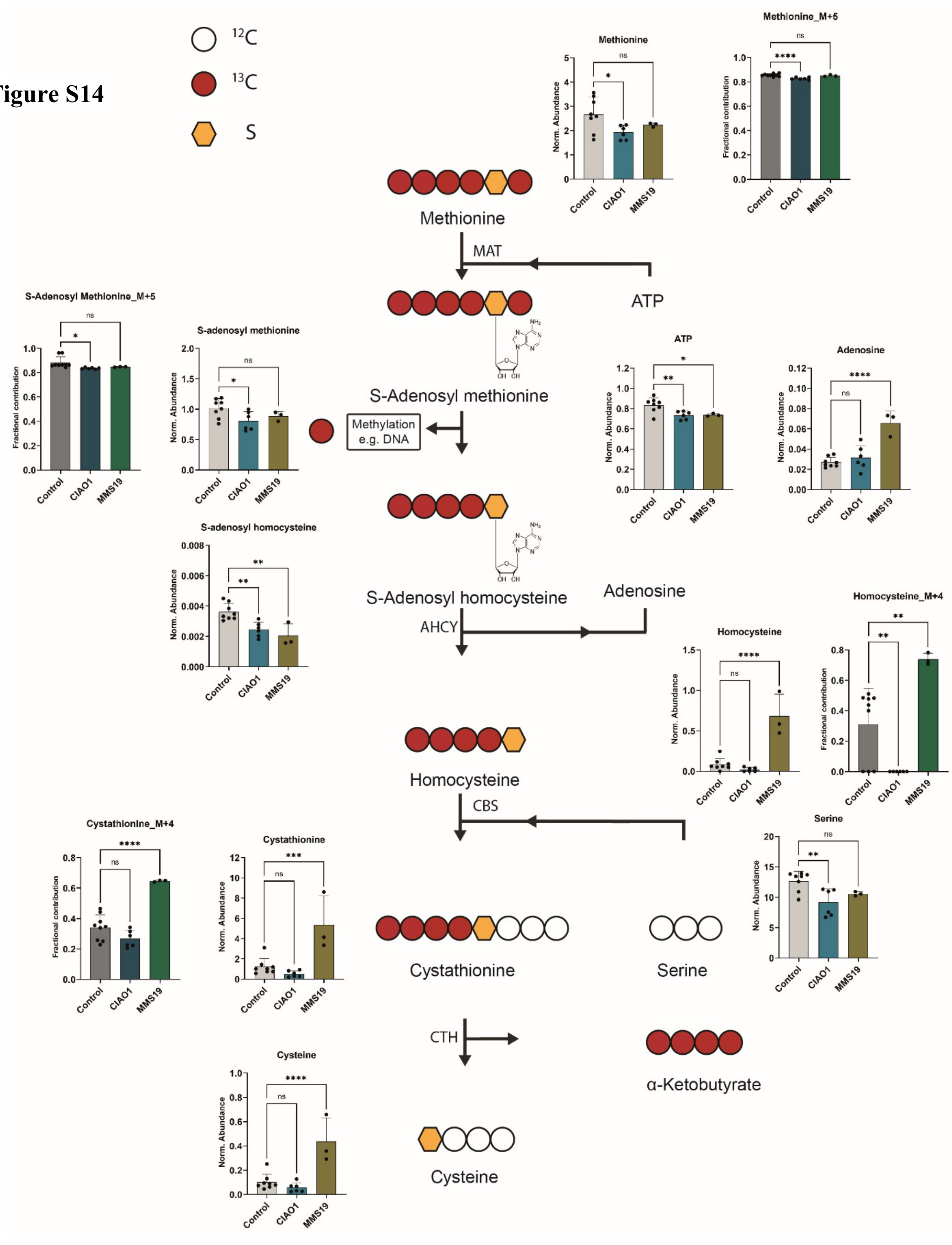

Figure S14. Metabolomics and flux analysis of stable-isotope labeled methionine. Mass isotopomer distributions of metabolites after introduction of fibroblasts of the CIAO1 and MMS19 patients and controls with $^{13}C_5$-Methionine. The mass isotopomer distributions were corrected for natural isotope abundances using mass isotopomer distribution analysis. MAT, methionine adenosyltransferase; AHCY, S-adenosylhomocysteine hydrolase; CBS, cystathionine-β-synthase; CTH, cystathionine-γ-lyase.

Figure S15

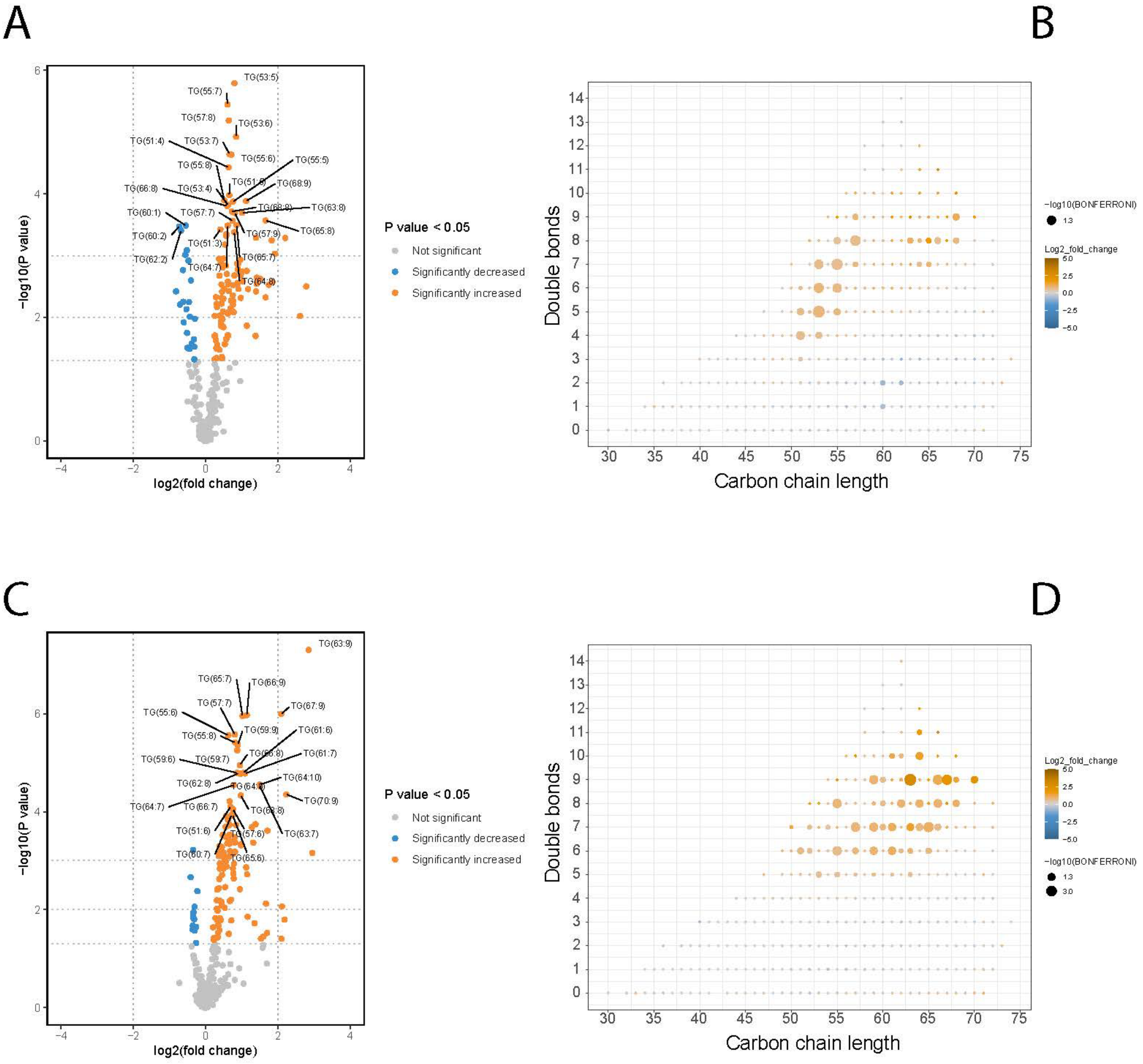

Figure S15. Altered triacylglycerols in CIAO1 and MMS19 deficient fibroblasts. Volcano plots depicting the triacylglycerols (TG) levels in CIAO1 patients (A) and the MMS19 patient (C). Significance cutoff is shown in the legend of the volcano plot. The three horizontal dotted lines indicate p-value 0.05, 0.01 and 0.001, respectively. The carbon-chain length of TG plotted versus the total number of double bonds in those chains in fibroblasts of the CIAO1 patients (B) and the MMS19 patient (D). Changes in the TG composition of CIAO1 and MMS19-deficient fibroblasts versus controls show significant increases of triacylglycerols with long carbon-chain length and double bonds.

Figure S16

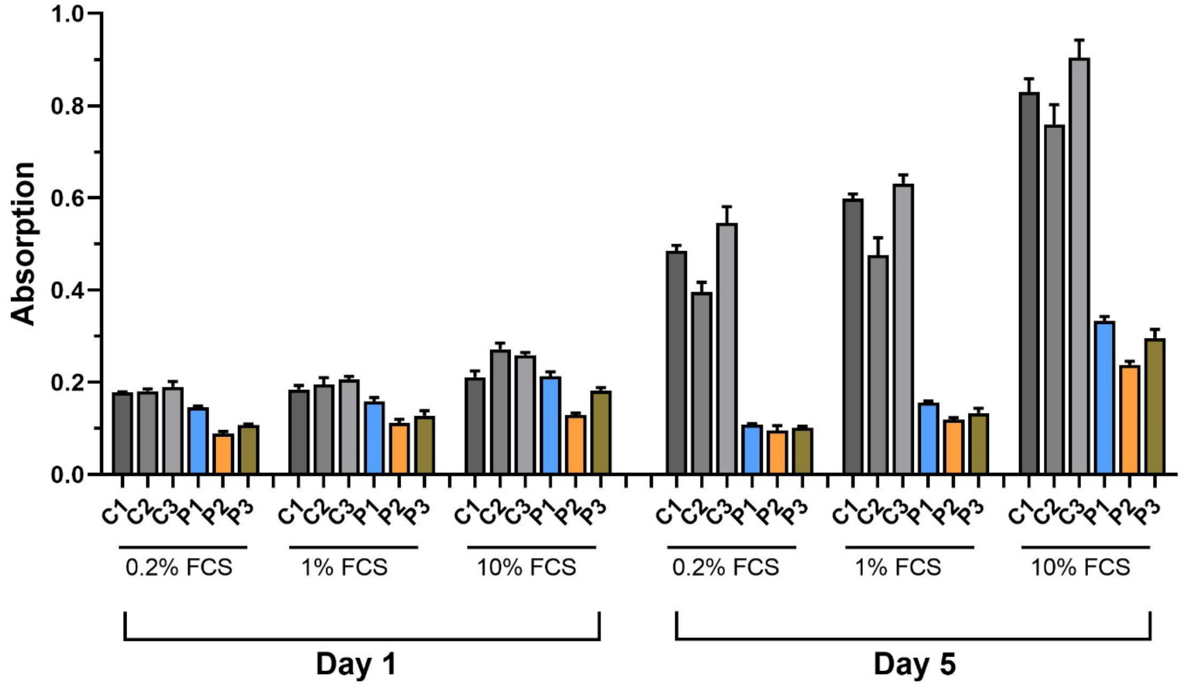

Figure S16. The effect of culturing conditions on proliferation. Fibroblasts were grown in culture medium containing 0.2%, 1% or 10% Fetal Calf Serum (FCS). The amount of viable cells was quantified on day 1 and day 5 by MTS analysis.

Figure S17

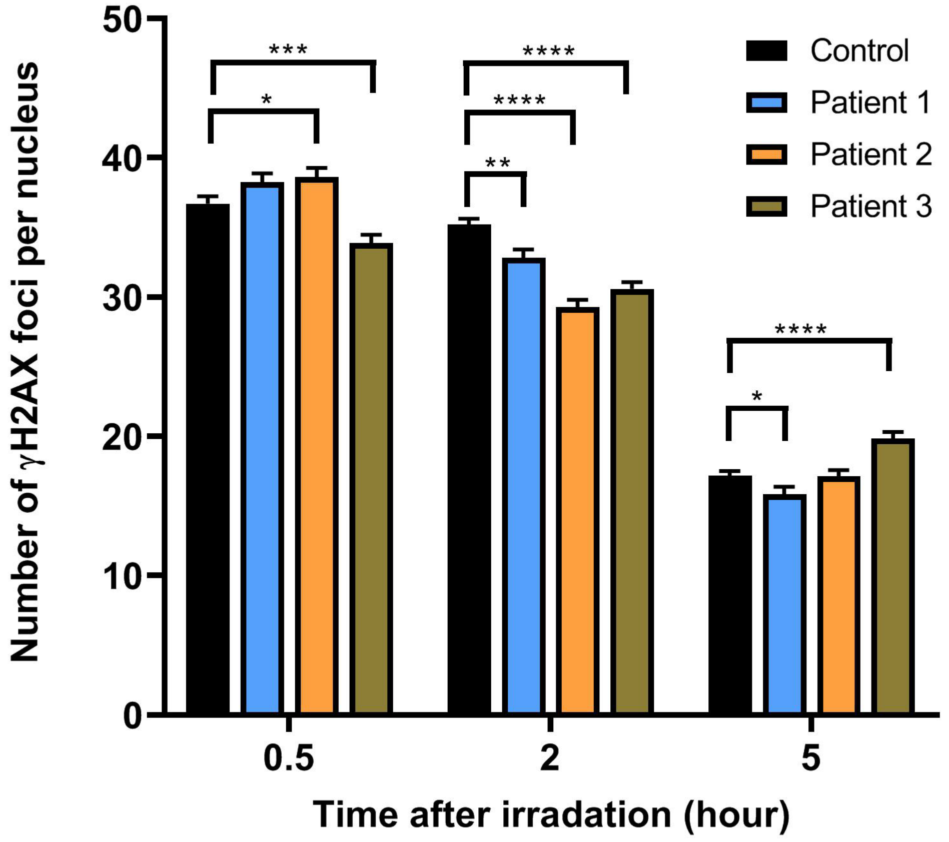

Figure S17. Detection of γ-H2AX foci. DNA double-strand breaks were detected by scoring of γ-H2AX foci, in 425 up to 987 individual cells. The bars represent the mean number of gH2AX foci per nucleus + SEM. The statistical significance between the control cell line and a patient cell line is depicted as * ($P < 0.05$); ** ($P < 0.01$); *** ($P < 0.001$); *** ($P < 0.0001$).

Figure S18

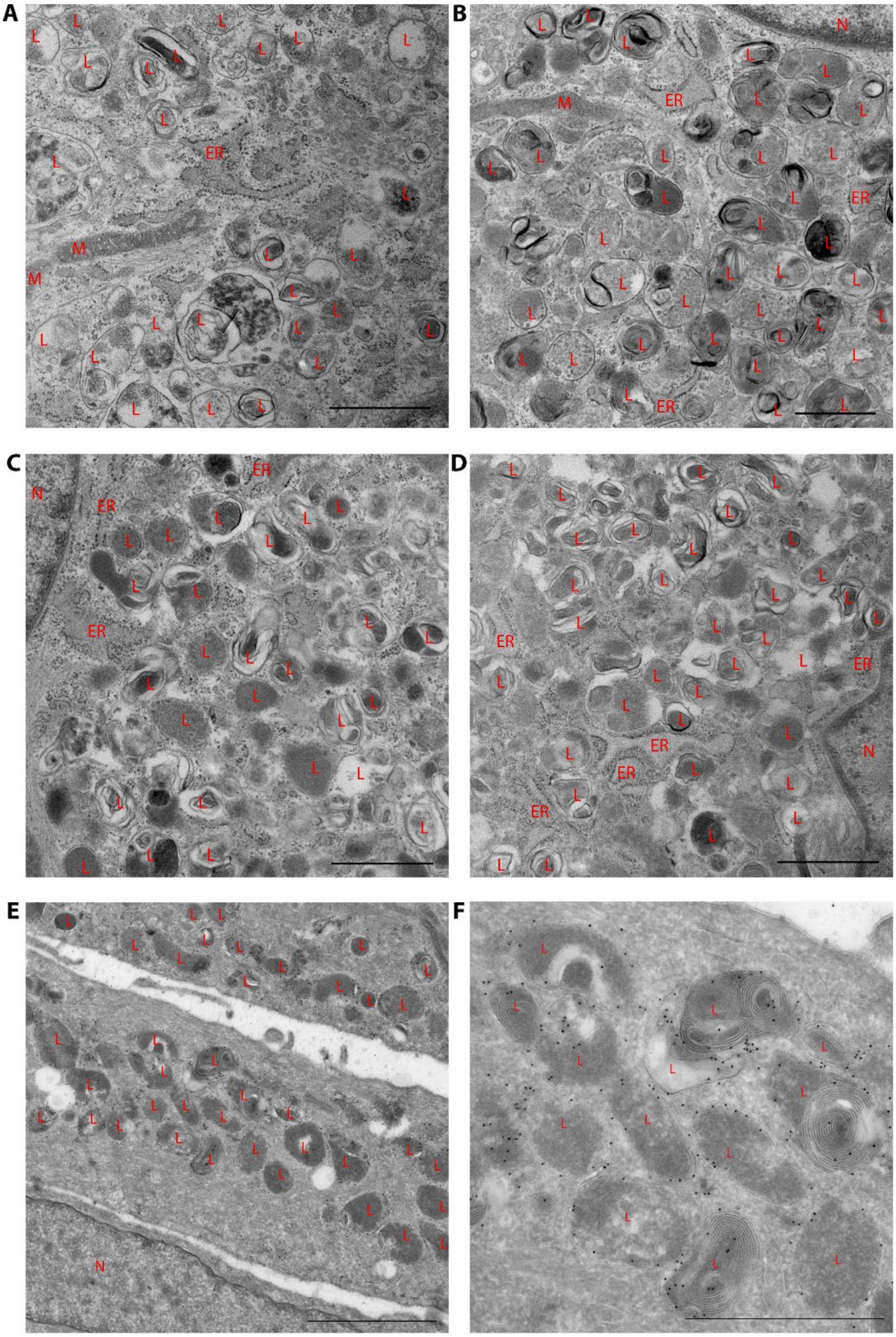

Figure S18. Electron microscopy images of fibroblasts. (A) (Control, Left top); (B) (patient 2, right top), (C) (Patient 1, middle left) and (D) (Patient 3, middle right) embedded in epon. Immunogold labelled fibroblast of patient 1 (E, bottom row) using CD63 and 10 nm gold indicating lysosomes. (F) (Right bottom figure) shows a higher magnification of a region with multiple electron-dense and multi-lamellar lysosomes. Cellular compartments are denoted L, lysosomes; N, nucleus; ER, endoplasmic reticulum; M, mitochondria and the bars represent 1 µm.

Figure S19

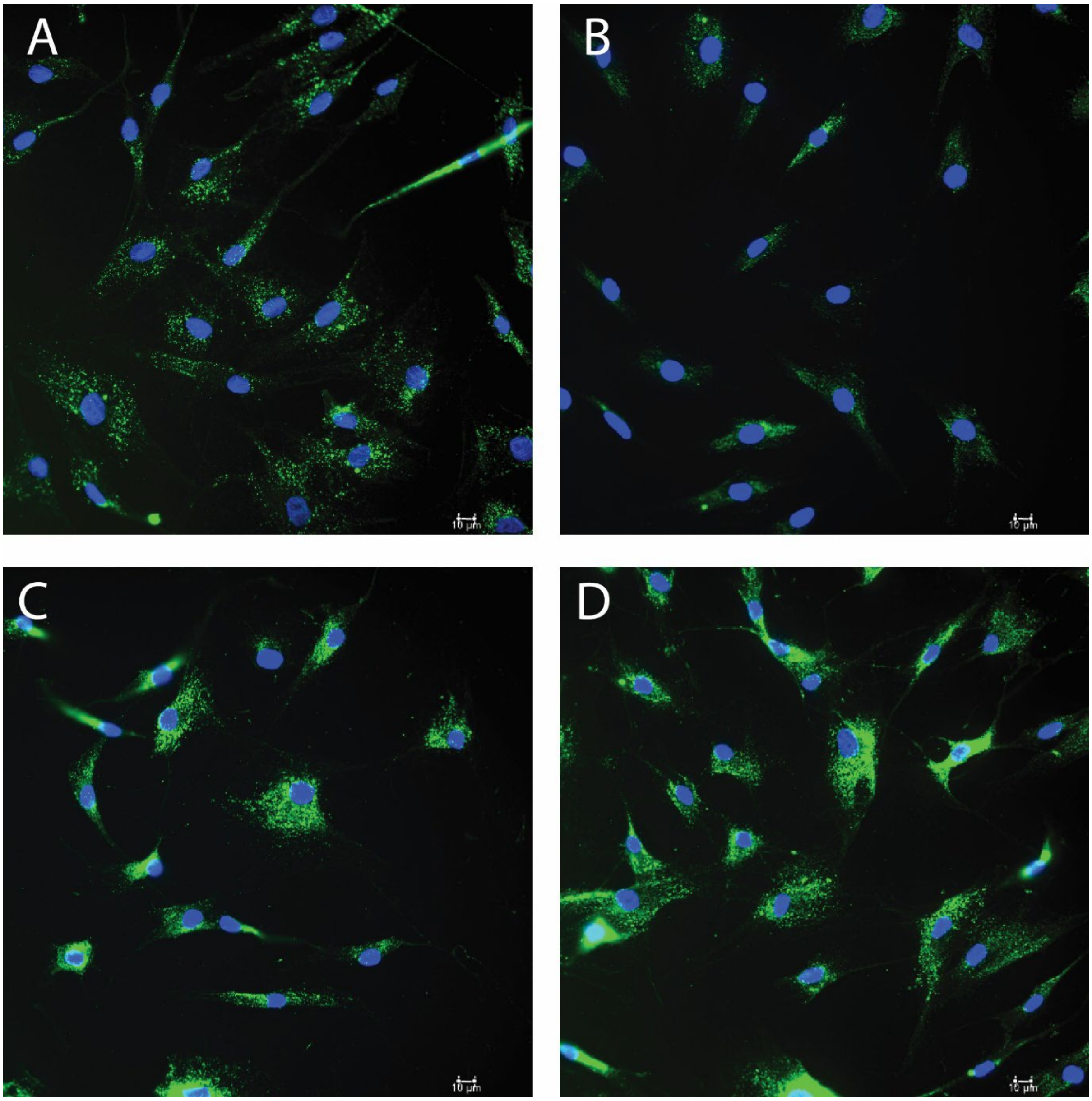

Figure S19. Immunofluorescent staining of lysosomes. The lysososmes were detected using a green fluorescent antibody against CD63, a multivesicular membrane protein. The nucleus (blue) was stained with DAPI. The immunofluorescent staining is shown for control cells (A), patient 1 (B), patient 2 (C) and patient 3 (D). The bars represent 10 µm.

Figure S20

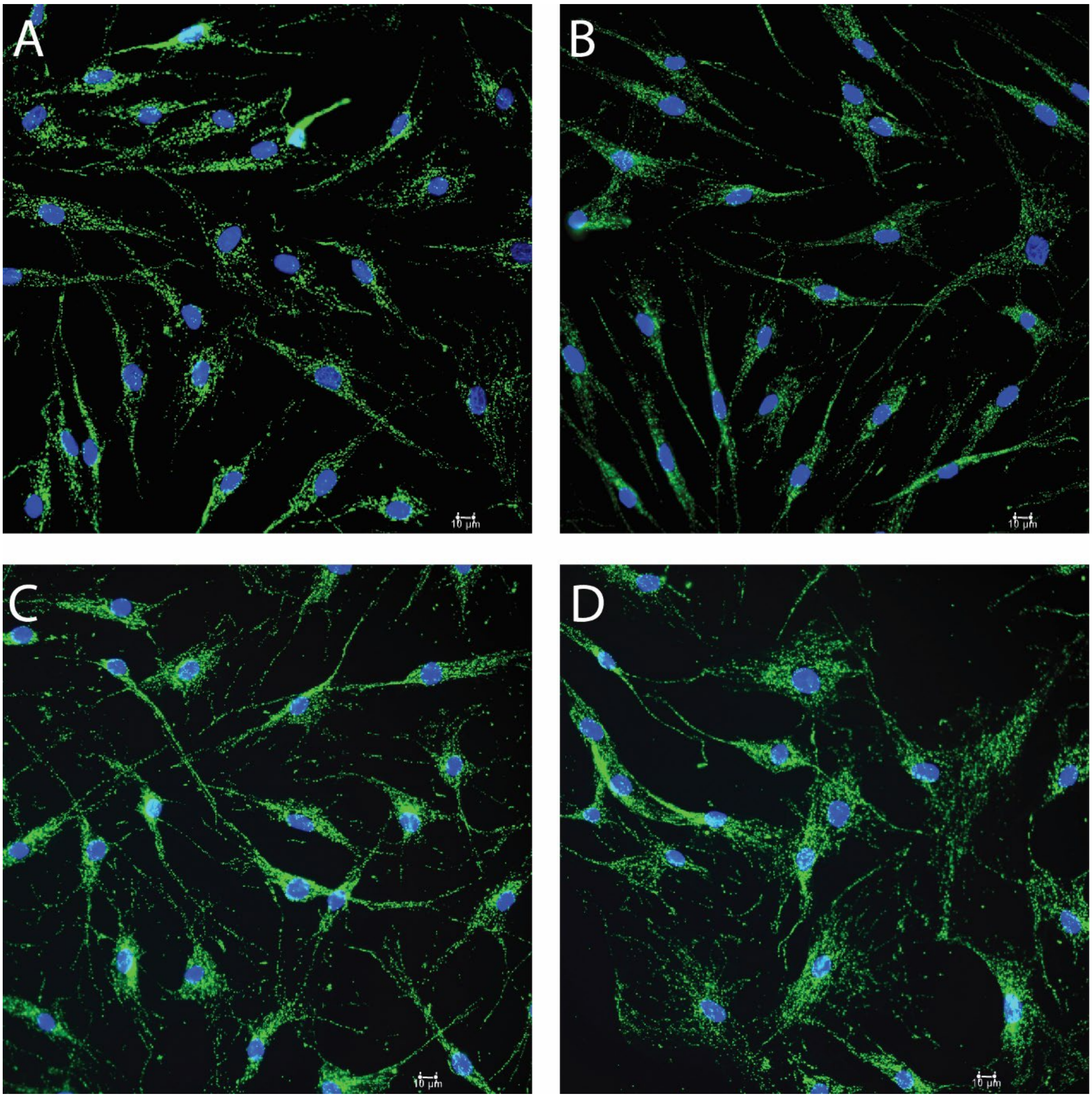

Figure S20. Immunofluorescent staining of mitochondria. The mitochondria were detected using a green fluorescent antibody against mitochondrial import receptor subunit TOM20. The nucleus (blue) was stained with DAPI. The immunofluorescent staining is shown for control cells (A), patient 1 (B), patient 2 (C) and patient 3 (D). The bars represent 10 µm.

Figure S21

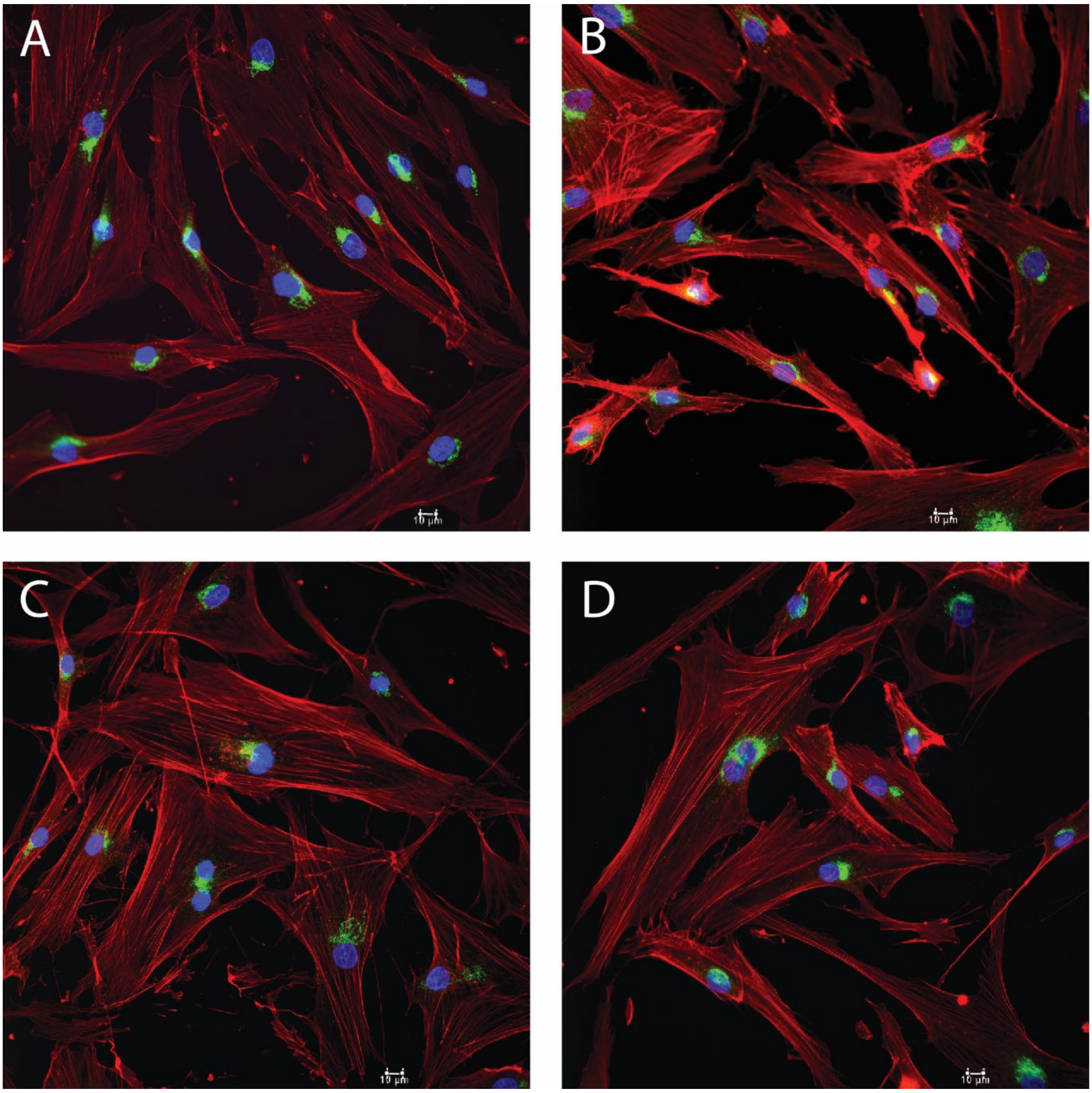

Figure S21. Immunofluorescent staining of the Golgi membrane and cytoskeleton. The Golgi was detected using a green fluorescent antibody against a RCAS1, a Golgi transmembrane protein. The cytoskeleton was stained (red) using Alexa Fluor® 555 Phalloidin which binds to F-actin. The nucleus (blue) was stained with DAPI. The immunofluorescent staining is shown for control cells (A), patient 1 (B), patient 2 (C) and patient 3 (D). The bars represent 10 μm.

Figure S22

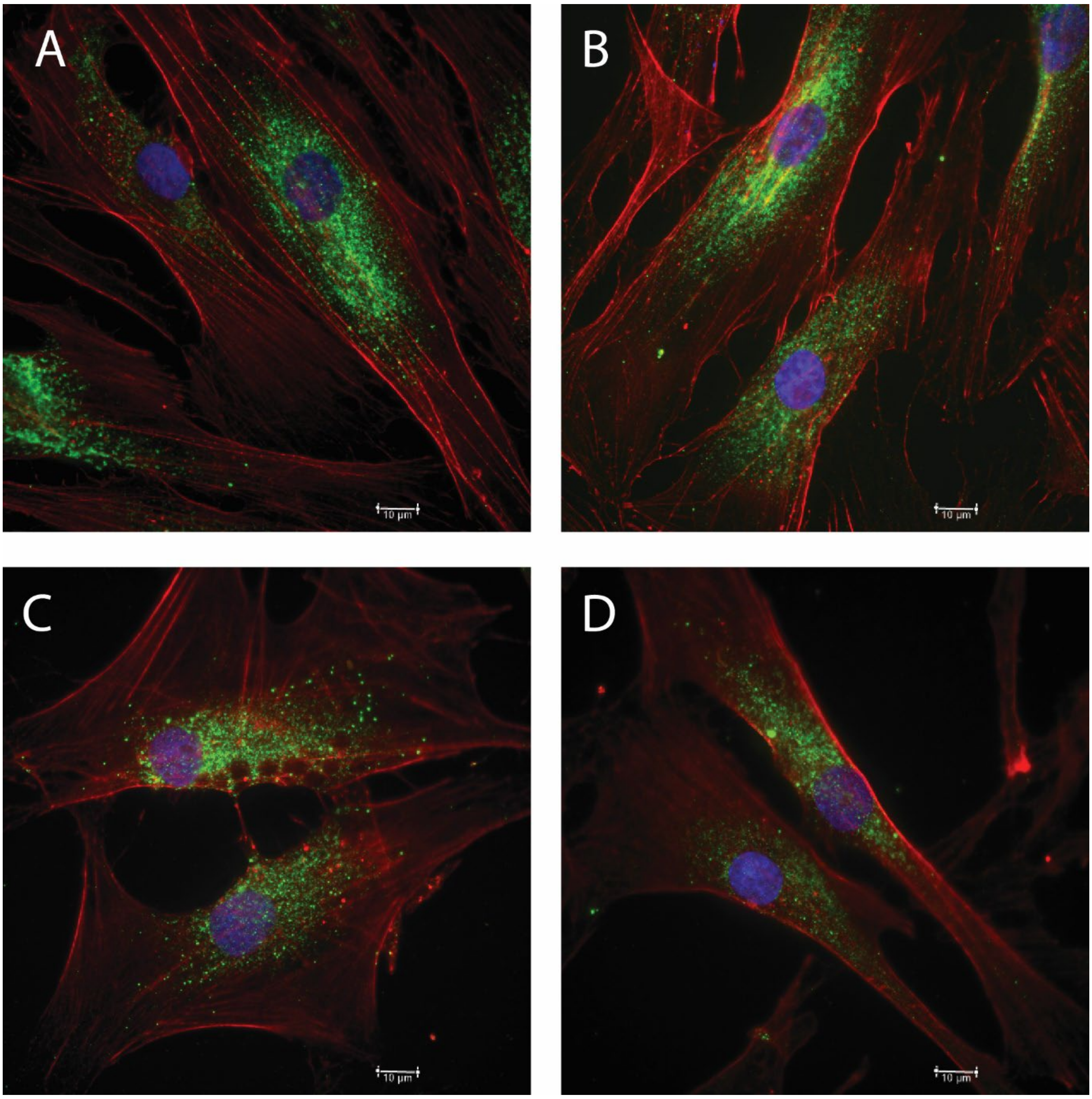

Figure S22. Immunofluorescent staining of the endoplasmic reticulum (ER) membrane and cytoskeleton. The ER was detected using a green fluorescent antibody against Protein Disulfide Isomerase. The cytoskeleton was stained (red) using Alexa Fluor® 555 Phalloidin which binds to F-actin. The nucleus (blue) was stained with DAPI. The immunofluorescent staining is shown for control cells (A), patient 1 (B), patient 2 (C) and patient 3 (D). The bars represent 10 μm.

Figure S23

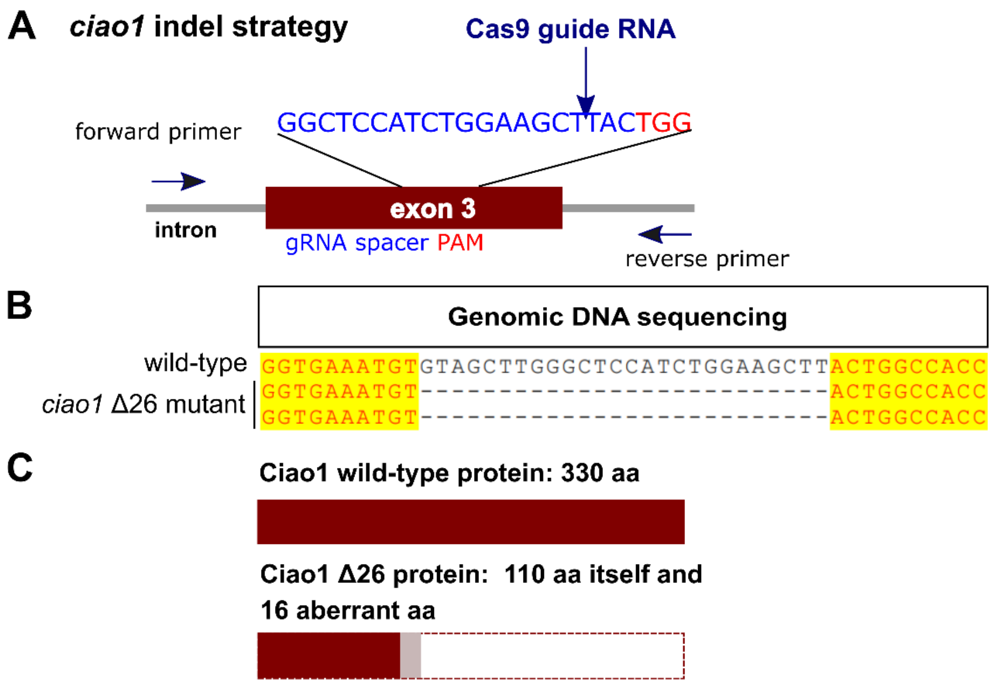

Figure S23. Development of a *ciao1*-/- zebrafish model using CRISPR/Cas9 (A) An indel strategy for targeting exon 3 of *ciao1* by a single guide RNA and Cas9. (B) The targeting strategy resulted in a 26-bp deletion overlapping the guide RNA spacer site. Intron, exon and primer features are indicated on the strategy schematic approximately to scale. The guide RNA feature is zoomed in for emphasis. (C) At the mRNA level, the 26-bp deletion in the exon 3 of *ciao1* is predicted to truncate two thirds of the resulting protein and generate 16 aberrant amino acids (aa).

Figure S24

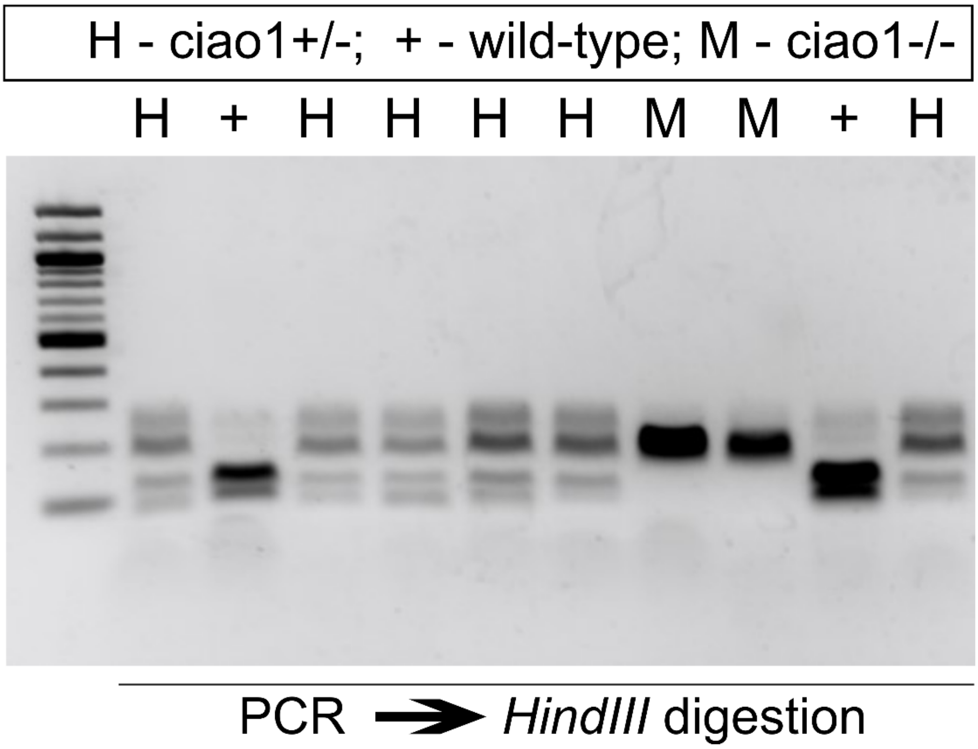

Figure S24. Genotyping of the *ciao1* 26-bp deletion mutant. Representative results of the genotyping strategy for larvae from the ciao1$^{\Delta 26/+}$ zebrafish incross. PCR followed by HindIII digestion generates different band patterns for different genotypes since the wild-type allele (+) PCR product can be fully digested, whereas the mutant allele (-) PCR product is resistant to digestion but somewhat smaller due to a 26-bp deletion. In the heterozygous samples, all bands are present as well as the digestion-resistant heteroduplex band. All samples are labeled with their inferred genotype, and the legend is shown.

Figure S25

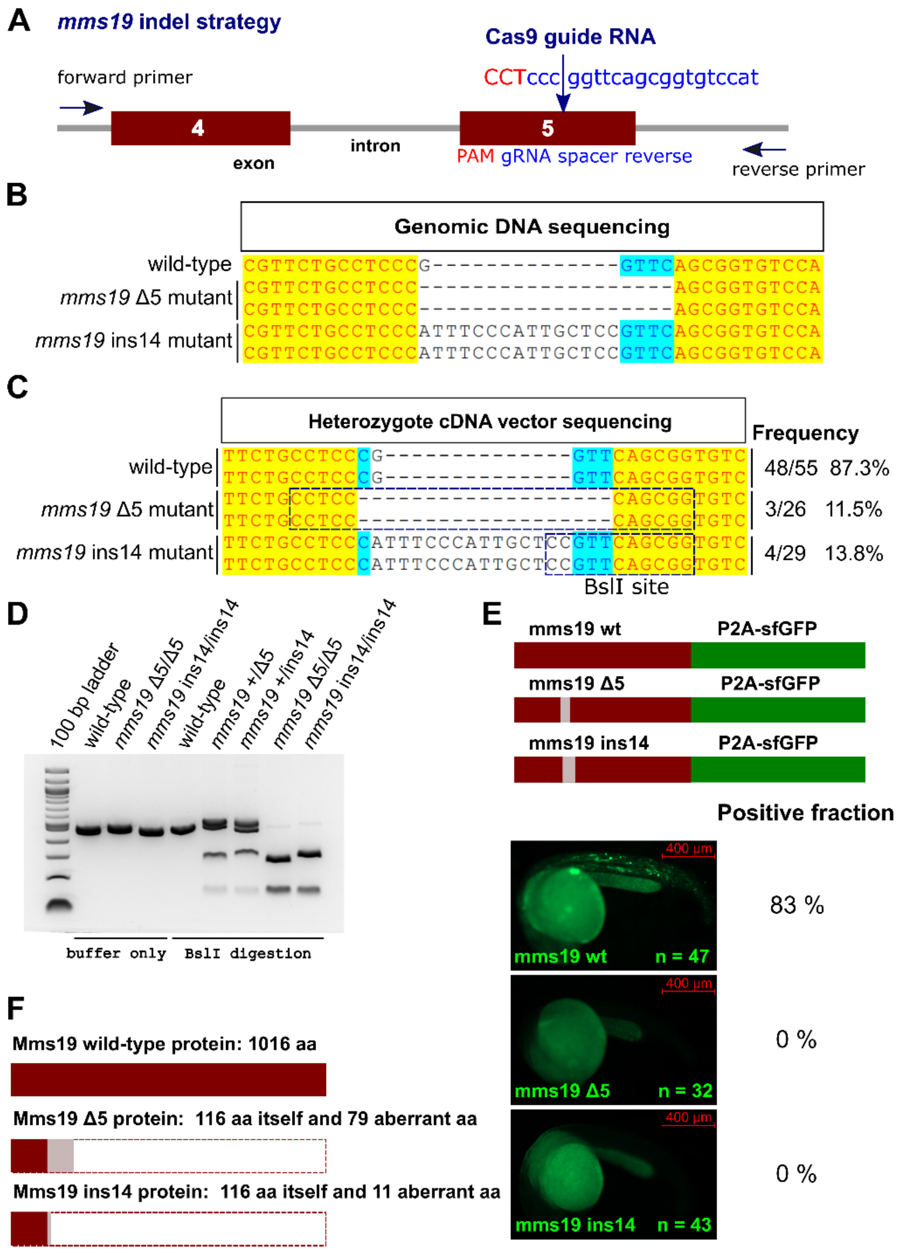

Figure S25. Development of an *mms19*-/- zebrafish model using CRISPR/Cas9 (A) CRISPR/Cas9-based mutation targeting strategy for exon 5 of *mms19* and primers used for genotyping. (B) Genomic of sequencing of individual F_2 wild-type and mutant embryos. Two sample sequences are shown for each genotype. (C) Cloning of *mms19* cDNA from mutant heterozygote results in dominant wild-type clones and decreased mutant bacterial clones. Two sample sequences are shown for each genotype as well as exact counts of each type of clone. (D) Genotyping strategy of genomic DNA samples using PCR product amplification followed by BslI digestion. (E) Cloning of 300-codon coding sequence fragments of wild-type and mutant genotypes into P2A-sfGFP fusion vector produces variant reporter vectors which label cells when the reading frame is not disrupted (wild-type[46]). Both *mms19* variants disrupt the coding sequence reading frame resulting in no fluorescent cell labeling. (F) Interpretation of variant consequences at the protein level. Scale bars (400 μm) on all zebrafish images indicate their actual sizes.

Figure S26

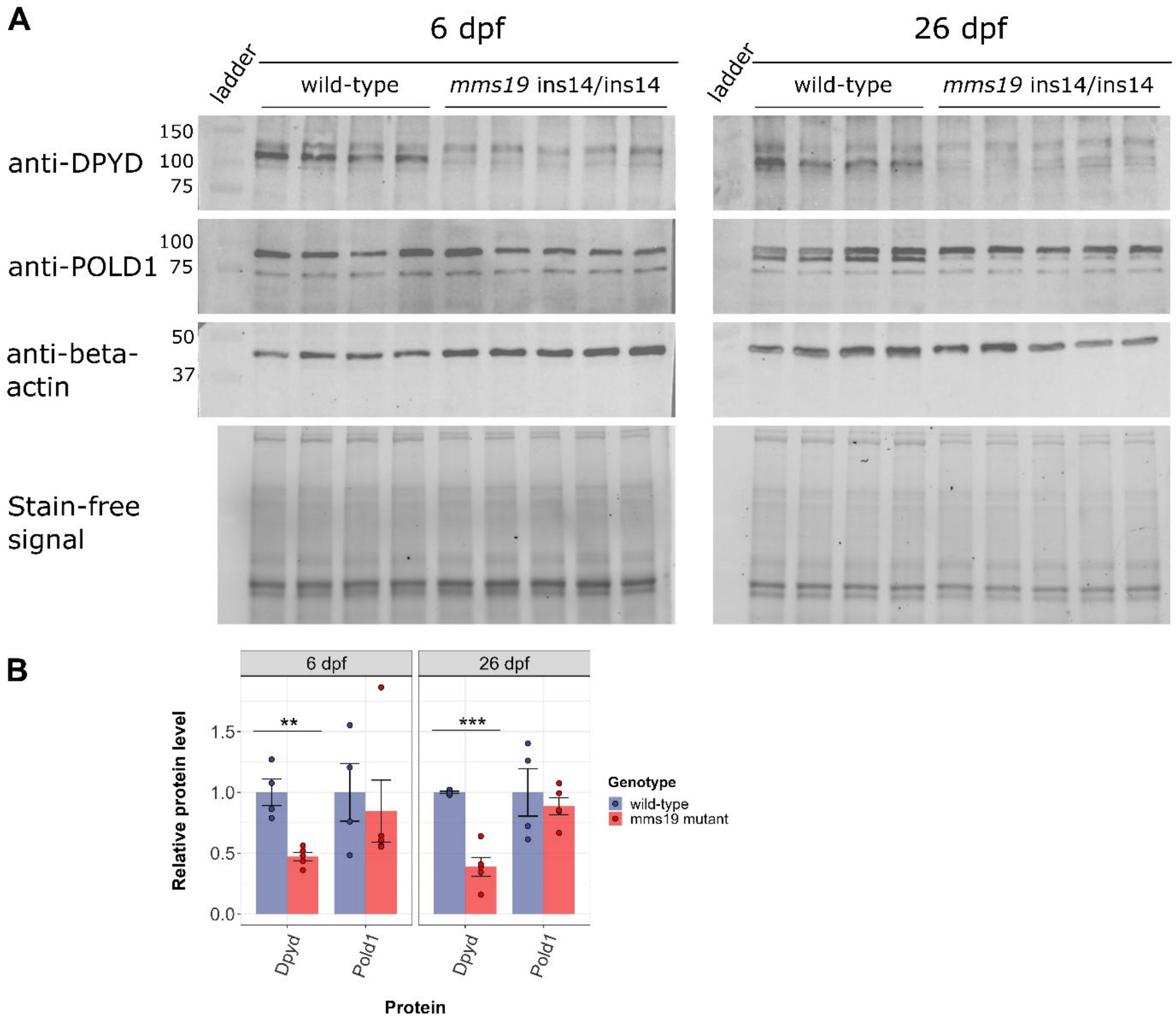

Figure S26. Quantification of two known Fe-S proteins in wild-type and *mms19* mutant zebrafish samples. (A) Western blotting results using human DPD, POLD1, β-Actin antibodies on the indicated zebrafish samples. Stain-free gel signal is shown as a robust loading control. (B) Quantification of protein levels based on the total protein stain-free signal. Significant differences in DPD protein levels between wild-type (n=4) and *mms19* mutant samples (n=5) were observed at both 6 dpf (**, Student's t-test P-value = 0.0014) and 26 dpf (***, Student's t-test P-value = 0.00021). In all graphs, bars indicate mean values and error bars indicate standard errors of the mean.

Figure S27

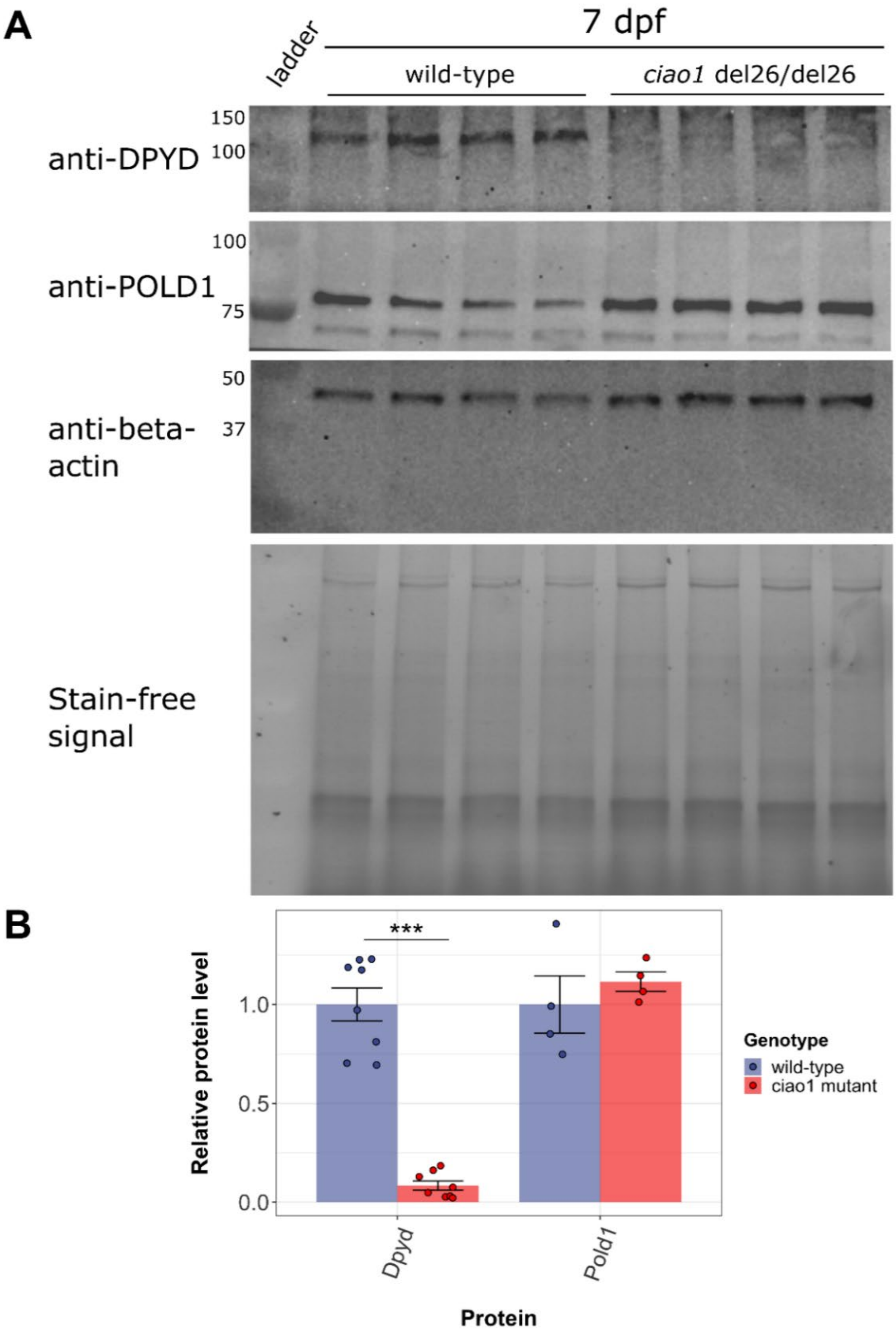

Figure S27. Quantification of two known Fe-S proteins in wild-type and *ciao1* mutant zebrafish samples. (A) Western blotting results using human DPD, POLD1, β-Actin antibodies on the indicated zebrafish samples. Stain-free gel signal is shown as a robust loading control. (B) Quantification of protein levels based on the total protein stain-free signal. Significant differences in DPD protein levels between wild-type (n=4) and *ciao1* mutant samples (n=4) were observed at 7 dpf (***, Student's t-test P-value = 4.49338E-08). In all graphs, bars indicate mean values and error bars indicate standard errors of the mean.

Figure S28

A

	Embryos from heterozygote cross				*Adults from heterozygote cross*			
	**delta5 mutant**		**ins14 mutant**		**delta5 mutant**		**ins14 mutant**	
	**Observed**	**Expected**	**Observed**	**Expected**	**Observed**	**Expected**	**Observed**	**Expected**
Wild-type	6	8	7	7.5	9	6	22	14
Heterozygote	18	16	14	15	15	12	34	28
Mutants	8	8	9	7.5	0	6	0	14

B

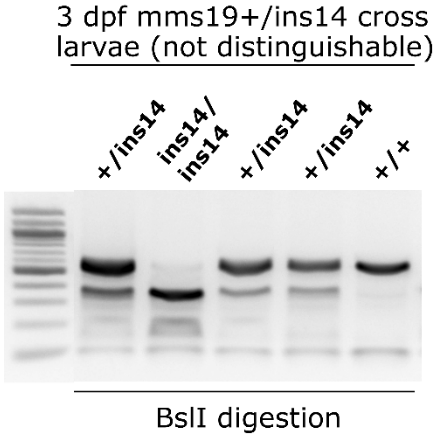

Figure S28. Variants in *mms19* lead to lethality after 3-5 weeks of development (A) Genotyping results of the progeny of both *mms19* mutant heterozygote incrosses during embryonic development and at adulthood show failure of homozygous mutants to survive. (B) Example larval genotyping results.

Figure S29

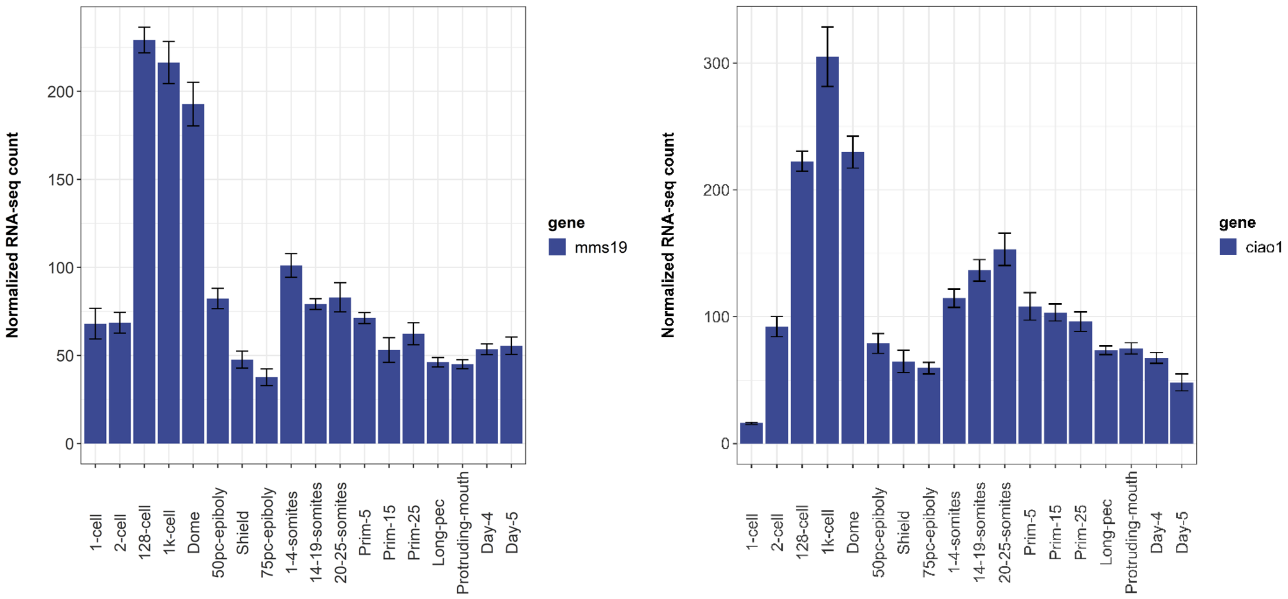

Figure S29. Temporal expression of CIA pathway genes *mms19* and *ciao1* in zebrafish. The systematic temporally-detailed RNA-sequencing data was downloaded from the article by White and colleagues[71], normalized by the total read depth at each stage and replicate and plotted from 1-cell (0 hpf) to Day-5 (120 hpf). The error bars indicate standard error of the mean based on 5 replicates at each stage. The relative height of the bar at 1-cell stage indicates the relative amount of maternal mRNA contribution for the gene.

SUPPLEMENTARY TABLES

Supplementary Table S1. Variants description

Patient	Gene	HGNC IDs	Genomic (hg19)		Transcripts		Proteins	
1	CIAO1	14280	g.96934282C>T	NC_000002.11	c.577C>T	NM_004804.2	p.(His193Tyr)	NP_004795.1
			g.96933112C>T		c.193C>T		p.(Arg65Trp)	
2	CIAO1	14280	g.96934257G>C		c.552G>C		p.(Trp184Cys)	
			g.96933112C>T		c.193C>T		p.(Arg65Trp)	
3	MMS19	13824	g.99236485_99236487del	NC_000010.10	c.637_639del	NM_001289405.1	p.(Glu213del)	NP_071757

Patient	Variant	Variant ID	ACMG criteria assigned	ACMG classification assigned
1	p.(His193Tyr)	rs902409423	PS3, PM2, PM3, PP3	Likely pathogenic
	p.(Arg65Trp)	rs11544859	PS3, PM2, PM3, PP3	Likely pathogenic
2	p.(Trp184Cys)	/	PS3, PM2, PM3, PP3	Likely pathogenic
	p.(Arg65Trp)	rs11544859	PS3, PM2, PM3, PP3	Likely pathogenic
3	p.(Glu213del)	/	PS3, PM2, PM4	Likely pathogenic

Supplementary Table S2. Antibodies used for immunoblotting

Protein	Primary antibody	Dilution	Secondary antibody	Dilution
**CIAO1**	CIAO1 (D1B4G) Rabbit mAb (Cell Signaling)	1:1.000	IRDye®800 Goat Anti-Rabbit LI-COR)	1:10.000
**MMS19**	MMS19 (D5J8J) Rabbit mAb (Cell Signaling)	1:1.000	IRDye®800 Goat Anti-Rabbit (LI-COR)	1:10.000
**MMS19**	MMS19 (G-12) Mouse mAb (SantaCruz)	1:1000	IRDye®800 Goat Anti-Mouse (LI-COR)	1:10.000
**GPAT**	PPAT Antibody (PA5-12333) Thermo Fisher	1:1.000	IRDye®800 Goat Anti-Rabbit (LI-COR)	1:10.000
**XPD**	XPD (D3Z6I) Rabbit mAb (Cell Signaling)	1:1.000	IRDye®800 Goat Anti-Rabbit (LI-COR)	1:10.000
**IRP**	IRP (D6S4J) Rabbit mAb (Cell Signaling)	1:1.000	IRDye®800 Goat Anti-Rabbit (LI-COR)	1:10.000
**DPD**	Anti-DPYD (H00001806-M01) mAb ABNOVA	1:1.000	Polyclonal Goat Anti-Mouse Immunoglobulins/HRP (P0447) Dako	1:2.000
**α-tubulin**	mouse antibody against α-tubulin (T6199) Sigma-Aldrich	1:5.000	IRDye®680 Donkey Anti-Mouse (LI-COR)	1:10.000
**V5**	V5 Tag (R960-25) mAb Thermo Fisher	1:1000	IRDye®800 Goat Anti-Mouse (LI-COR)	1:10.000
**α-tubulin**	a-tubuline against Rabbit (2144S) Cell Signaling	1:5.000	IRDye®680 Donkey Anti-Rabbit (LI-COR)	1:10.000

Supplementary Table S3. Oligos used for generating and genotyping *mms19* zebrafish

Oligo name	Sequence	Purpose
mms19_sgRNA_sense	TAATACGACTCACTATAGGATGGACACCGCTGAACCG GGGTTTTAGAGCTAGAAATAGC	sgRNA synthesis
Rev_sgRNA_scaffold	ggatccGCACCGACTCGGTGCCACTTTTTCAAGTTGATAA CGGACTAGCCTTATTTTAACTTGCTATTTCTAGCTCTAA AAC	sgRNA synthesis
mms19_EX45_del_for	TGATGCTTTGTGTTTGGCTTA	Genotyping
mms19_EX45_del_rev	CAGAGGCTTTAGAACAGTAACAAAAA	Genotyping
PacI-T3_mms19_mutrep_for	GAGGTTAATTAAAATTAACCCTCACTAAAGGTTTGAA GCCCTGACTGGCT	Variant reporter cloning
AscI-mms19_mutrep_2_rev	GATCGGCGCGCCcatggagtccactttagcgc	Variant reporter cloning
mms19_ISH_for	gactcgtggcagaggagttc	Variant reporter cloning
mms19_ISH-3_for	cggtttctctgtggaggaag	In situ probe
T7_mms19_ISH-3_rev	TAATACGACTCACTATAGGGaggcagacgagacagagcat	In situ probe

REFERENCES

1. van Kuilenburg, A.B., Meijer, J., Maurer, D., Dobritzsch, D., Meinsma, R., Los, M., Knegt, L.C., Zoetekouw, L., Jansen, R.L., Dezentje, V., et al. (2017). Severe fluoropyrimidine toxicity due to novel and rare DPYD missense mutations, deletion and genomic amplification affecting DPD activity and mRNA splicing. Biochim Biophys Acta *1863*, 721-730. 10.1016/j.bbadis.2016.12.010.
2. van Kuilenburg, A.B.P., Meijer, J., Mul, A.N.P., Hennekam, R.C.M., Hoovers, J.M.N., de Die-Smulders, C.E.M., Weber, P., Mori, A.C., Bierau, J., Fowler, B., et al. (2009). Analysis of severely affected patients with dihydropyrimidine dehydrogenase deficiency reveals large intragenic rearrangements of DPYD and a de novo interstitial deletion del(1)(p13.3p21.3). Hum Genet *125*, 581-590. 10.1007/s00439-009-0653-6.
3. van Kuilenburg, A.B.P., Tarailo-Graovac, M., Meijer, J., Drogemoller, B., Vockley, J., Maurer, D., Dobritzsch, D., Ross, C.J., Wasserman, W., Meinsma, R., Zoetekouw, L., and van Karnebeek, C.D.M. (2018). Genome sequencing reveals a novel genetic mechanism underlying dihydropyrimidine dehydrogenase deficiency: A novel missense variant c.1700G > A and a large intragenic inversion in DPYD spanning intron 8 to intron 12. Hum. Mutat. *39*, 947-953. 10.1002/humu.23538.
4. van Kuilenburg, A.B.P., van Lenthe, H., Tromp, A., Veltman, P.C.J., and van Gennip, A.H. (2000). Pitfalls in the diagnosis of patients with a partial dihydropyrimidine dehydrogenase deficiency. Clin Chem *46*, 9-17. 10620566.
5. van Lenthe, H., van Kuilenburg, A.B.P., Ito, T., Bootsma, A.H., van Cruchten, A.G., Wada, Y., and van Gennip, A.H. (2000). Defects in pyrimidine degradation identified by HPLC-electrospray tandem mass spectrometry of urine specimens or urine-soaked filter paper strips. Clin Chem *46*, 1916-1922. 11106323.
6. van Kuilenburg, A.B.P., van Lenthe, H., van Cruchten, A.G., and Kulik, W. (2004). Quantification of 5,6-dihydrouracil by HPLC-electrospray tandem mass spectrometry. Clin Chem *50*, 236-238. 10.1373/clinchem.2003.026229.
7. Ebberink, M.S., Mooijer, P.A., Gootjes, J., Koster, J., Wanders, R.J., and Waterham, H.R. (2011). Genetic classification and mutational spectrum of more than 600 patients with a Zellweger syndrome spectrum disorder. Hum. Mutat. *32*, 59-69. 10.1002/humu.21388.
8. Li, H., and Durbin, R. (2009). Fast and accurate short read alignment with Burrows-Wheeler transform. Bioinformatics *25*, 1754-1760. 10.1093/bioinformatics/btp324.
9. Pedersen, B.S., and Quinlan, A.R. (2018). Mosdepth: quick coverage calculation for genomes and exomes. Bioinformatics *34*, 867-868. 10.1093/bioinformatics/btx699.
10. Tarailo-Graovac, M., Shyr, C., Ross, C.J., Horvath, G.A., Salvarinova, R., Ye, X.C., Zhang, L.H., Bhavsar, A.P., Lee, J.J., Drogemoller, B.I., et al. (2016). Exome Sequencing and the Management of Neurometabolic Disorders. N. Engl. J. Med. *374*, 2246-2255. 10.1056/NEJMoa1515792.
11. Van der Auwera, G.A., Carneiro, M.O., Hartl, C., Poplin, R., Del Angel, G., Levy-Moonshine, A., Jordan, T., Shakir, K., Roazen, D., Thibault, J., et al. (2013). From FastQ data to high confidence variant calls: the Genome Analysis Toolkit best practices pipeline. Current protocols in bioinformatics *43*, 11.10.11-11.10.33. 10.1002/0471250953.bi1110s43.
12. Pedersen, B.S., Layer, R.M., and Quinlan, A.R. (2016). Vcfanno: fast, flexible annotation of genetic variants. Genome Biol. *17*, 118. 10.1186/s13059-016-0973-5.
13. van Kuilenburg, A.B.P., Tarailo-Graovac, M., Richmond, P.A., Drogemoller, B.I., Pouladi, M.A., Leen, R., Brand-Arzamendi, K., Dobritzsch, D., Dolzhenko, E., Eberle, M.A., et al. (2019). Glutaminase Deficiency Caused by Short Tandem Repeat Expansion in GLS. N. Engl. J. Med. *380*, 1433-1441. 10.1056/NEJMoa1806627.

14. Layer, R.M., Chiang, C., Quinlan, A.R., and Hall, I.M. (2014). LUMPY: a probabilistic framework for structural variant discovery. Genome Biol. *15*, R84. 10.1186/gb-2014-15-6-r84.
15. Geoffroy, V., Herenger, Y., Kress, A., Stoetzel, C., Piton, A., Dollfus, H., and Muller, J. (2018). AnnotSV: an integrated tool for structural variations annotation. Bioinformatics *34*, 3572-3574. 10.1093/bioinformatics/bty304.
16. Gardner, E.J., Lam, V.K., Harris, D.N., Chuang, N.T., Scott, E.C., Pittard, W.S., Mills, R.E., Genomes Project, C., and Devine, S.E. (2017). The Mobile Element Locator Tool (MELT): population-scale mobile element discovery and biology. Genome Res. *27*, 1916-1929. 10.1101/gr.218032.116.
17. Calabrese, C., Simone, D., Diroma, M.A., Santorsola, M., Guttà, C., Gasparre, G., Picardi, E., Pesole, G., and Attimonelli, M. (2014). MToolBox: a highly automated pipeline for heteroplasmy annotation and prioritization analysis of human mitochondrial variants in high-throughput sequencing. Bioinformatics *30*, 3115-3117. 10.1093/bioinformatics/btu483.
18. Chatzispyrou, I.A., Alders, M., Guerrero-Castillo, S., Zapata Perez, R., Haagmans, M.A., Mouchiroud, L., Koster, J., Ofman, R., Baas, F., Waterham, H.R., et al. (2017). A homozygous missense mutation in ERAL1, encoding a mitochondrial rRNA chaperone, causes Perrault syndrome. Hum. Mol. Genet. *26*, 2541-2550. 10.1093/hmg/ddx152.
19. Dull, T., Zufferey, R., Kelly, M., Mandel, R.J., Nguyen, M., Trono, D., and Naldini, L. (1998). A third-generation lentivirus vector with a conditional packaging system. J. Virol. *72*, 8463-8471.
20. Jumper, J., Evans, R., Pritzel, A., Green, T., Figurnov, M., Ronneberger, O., Tunyasuvunakool, K., Bates, R., Žídek, A., Potapenko, A., et al. (2021). Highly accurate protein structure prediction with AlphaFold. Nature *596*, 583-589. 10.1038/s41586-021-03819-2.
21. Varadi, M., Anyango, S., Deshpande, M., Nair, S., Natassia, C., Yordanova, G., Yuan, D., Stroe, O., Wood, G., Laydon, A., et al. (2022). AlphaFold Protein Structure Database: massively expanding the structural coverage of protein-sequence space with high-accuracy models. Nucleic Acids Res. *50*, D439-d444. 10.1093/nar/gkab1061.
22. Emsley, P., Lohkamp, B., Scott, W.G., and Cowtan, K. (2010). Features and development of Coot. Acta Crystallogr D Biol Crystallogr *66*, 486-501. 10.1107/S0907444910007493.
23. DeLano, W.L. (2002). *The PyMOL Molecular Graphics System*. DeLano Scientific, Palo Alto, CA, USA. http://www.pymol.org.
24. Franken, N.A., Hovingh, S., Ten Cate, R., Krawczyk, P., Stap, J., Hoebe, R., Aten, J., and Barendsen, G.W. (2012). Relative biological effectiveness of high linear energy transfer α-particles for the induction of DNA-double-strand breaks, chromosome aberrations and reproductive cell death in SW-1573 lung tumour cells. Oncol. Rep. *27*, 769-774. 10.3892/or.2011.1604.
25. Hughes, C.S., Foehr, S., Garfield, D.A., Furlong, E.E., Steinmetz, L.M., and Krijgsveld, J. (2014). Ultrasensitive proteome analysis using paramagnetic bead technology. Mol. Syst. Biol. *10*, 757. 10.15252/msb.20145625.
26. Sherman, B.T., Hao, M., Qiu, J., Jiao, X., Baseler, M.W., Lane, H.C., Imamichi, T., and Chang, W. (2022). DAVID: a web server for functional enrichment analysis and functional annotation of gene lists (2021 update). Nucleic Acids Res. *50*, W216-W221. 10.1093/nar/gkac194.
27. Molenaars, M., Schomakers, B.V., Elfrink, H.L., Gao, A.W., Vervaart, M.A.T., Pras-Raves, M.L., Luyf, A.C., Smith, R.L., Sterken, M.G., Kammenga, J.E., et al. (2021). Metabolomics and lipidomics in Caenorhabditis elegans using a single-sample preparation. Dis. Model. Mech. *14*. 10.1242/dmm.047746.
28. Schomakers, B.V., Hermans, J., Jaspers, Y.R.J., Salomons, G., Vaz, F.M., van Weeghel, M., and Houtkooper, R.H. (2022). Polar metabolomics in human muscle biopsies using a liquid-liquid extraction and full-scan LC-MS. STAR Protocols *3*, 101302. 10.1016/j.xpro.2022.101302.
29. Gentleman, R.C., Carey, V.J., Bates, D.M., Bolstad, B., Dettling, M., Dudoit, S., Ellis, B., Gautier, L., Ge, Y., Gentry, J., et al. (2004). Bioconductor: open software development for computational biology and bioinformatics. Genome Biol. *5*, R80. 10.1186/gb-2004-5-10-r80.

30. Rohart, F., Gautier, B., Singh, A., and KA, L.C. (2017). mixOmics: An R package for 'omics feature selection and multiple data integration. PLoS Comput. Biol. *13*, e1005752. 10.1371/journal.pcbi.1005752.
31. Ritchie, M.E., Phipson, B., Wu, D., Hu, Y., Law, C.W., Shi, W., and Smyth, G.K. (2015). limma powers differential expression analyses for RNA-sequencing and microarray studies. Nucleic Acids Res. *43*, e47-e47. 10.1093/nar/gkv007.
32. Law, C.W., Chen, Y., Shi, W., and Smyth, G.K. (2014). voom: precision weights unlock linear model analysis tools for RNA-seq read counts. Genome Biol. *15*, R29. 10.1186/gb-2014-15-2-r29.
33. Wickham, H. (2016). Programming with ggplot2. In ggplot2: Elegant Graphics for Data Analysis, (Springer International Publishing), pp. 241-253. 10.1007/978-3-319-24277-4_12.
34. Held, N.M., Kuipers, E.N., van Weeghel, M., van Klinken, J.B., Denis, S.W., Lombès, M., Wanders, R.J., Vaz, F.M., Rensen, P.C.N., Verhoeven, A.J., Boon, M.R., and Houtkooper, R.H. (2018). Pyruvate dehydrogenase complex plays a central role in brown adipocyte energy expenditure and fuel utilization during short-term beta-adrenergic activation. Sci. Rep. *8*, 9562. 10.1038/s41598-018-27875-3.
35. Held, N.M., Buijink, M.R., Elfrink, H.L., Kooijman, S., Janssens, G.E., Luyf, A.C.M., Pras-Raves, M.L., Vaz, F.M., Michel, S., Houtkooper, R.H., and van Weeghel, M. (2021). Aging selectively dampens oscillation of lipid abundance in white and brown adipose tissue. Sci. Rep. *11*, 5932. 10.1038/s41598-021-85455-4.
36. Mardinoglu, A., Agren, R., Kampf, C., Asplund, A., Uhlen, M., and Nielsen, J. (2014). Genome-scale metabolic modelling of hepatocytes reveals serine deficiency in patients with non-alcoholic fatty liver disease. Nature communications *5*, 3083. 10.1038/ncomms4083.
37. Hastings, J., Owen, G., Dekker, A., Ennis, M., Kale, N., Muthukrishnan, V., Turner, S., Swainston, N., Mendes, P., and Steinbeck, C. (2016). ChEBI in 2016: Improved services and an expanding collection of metabolites. Nucleic Acids Res. *44*, D1214-1219. 10.1093/nar/gkv1031.
38. Uhlén, M., Fagerberg, L., Hallström, B.M., Lindskog, C., Oksvold, P., Mardinoglu, A., Sivertsson, Å., Kampf, C., Sjöstedt, E., Asplund, A., et al. (2015). Proteomics. Tissue-based map of the human proteome. Science *347*, 1260419. 10.1126/science.1260419.
39. The Genotype-Tissue Expression (GTEx) project. (2013). Nat. Genet. *45*, 580-585. 10.1038/ng.2653.
40. Lill, R., and Freibert, S.A. (2020). Mechanisms of Mitochondrial Iron-Sulfur Protein Biogenesis. Annu Rev Biochem *89*, 471-499. 10.1146/annurev-biochem-013118-111540.
41. Westerfield, M. (2000). The zebrafish book. A guide for the laboratory use of zebrafish (Danio rerio), 4th ed. Edition (Univ. of Oregon Press).
42. White, R.M., Sessa, A., Burke, C., Bowman, T., LeBlanc, J., Ceol, C., Bourque, C., Dovey, M., Goessling, W., Burns, C.E., and Zon, L.I. (2008). Transparent adult zebrafish as a tool for in vivo transplantation analysis. Cell Stem Cell *2*, 183-189. 10.1016/j.stem.2007.11.002.
43. Pena, I.A., Roussel, Y., Daniel, K., Mongeon, K., Johnstone, D., Weinschutz Mendes, H., Bosma, M., Saxena, V., Lepage, N., Chakraborty, P., et al. (2017). Pyridoxine-Dependent Epilepsy in Zebrafish Caused by Aldh7a1 Deficiency. Genetics *207*, 1501-1518. 10.1534/genetics.117.300137.
44. Sander, J.D., Maeder, M.L., Reyon, D., Voytas, D.F., Joung, J.K., and Dobbs, D. (2010). ZiFiT (Zinc Finger Targeter): an updated zinc finger engineering tool. Nucleic Acids Res. *38*, W462-468. 10.1093/nar/gkq319.
45. Zhu, X., Xu, Y., Yu, S., Lu, L., Ding, M., Cheng, J., Song, G., Gao, X., Yao, L., Fan, D., et al. (2014). An efficient genotyping method for genome-modified animals and human cells generated with CRISPR/Cas9 system. Sci. Rep. *4*, 6420. 10.1038/srep06420.
46. Prykhozhij, S.V., Steele, S.L., Razaghi, B., and Berman, J.N. (2017). A rapid and effective method for screening, sequencing and reporter verification of engineered frameshift mutations in zebrafish. Dis. Model. Mech. *10*, 811-822. 10.1242/dmm.026765.

47. Hill, J.T., Demarest, B.L., Bisgrove, B.W., Su, Y.C., Smith, M., and Yost, H.J. (2014). Poly peak parser: Method and software for identification of unknown indels using sanger sequencing of polymerase chain reaction products. Dev. Dyn. *243*, 1632-1636. 10.1002/dvdy.24183.
48. Kosuta, C., Daniel, K., Johnstone, D.L., Mongeon, K., Ban, K., LeBlanc, S., MacLeod, S., Et-Tahiry, K., Ekker, M., MacKenzie, A., and Pena, I. (2018). High-throughput DNA Extraction and Genotyping of 3dpf Zebrafish Larvae by Fin Clipping. Journal of visualized experiments : JoVE. 10.3791/58024.
49. Lauter, G., Söll, I., and Hauptmann, G. (2011). Two-color fluorescent in situ hybridization in the embryonic zebrafish brain using differential detection systems. BMC Dev. Biol. *11*, 43. 10.1186/1471-213x-11-43.
50. Therneau, T. (2022). A Package for Survival Analysis in R. https://CRAN.R-project.org/package=survival.
51. Schindelin, J., Arganda-Carreras, I., Frise, E., Kaynig, V., Longair, M., Pietzsch, T., Preibisch, S., Rueden, C., Saalfeld, S., Schmid, B., et al. (2012). Fiji: an open-source platform for biological-image analysis. Nat. Methods *9*, 676-682. 10.1038/nmeth.2019.
52. Eslami, A., and Lujan, J. (2010). Western blotting: sample preparation to detection. Journal of visualized experiments : JoVE. 10.3791/2359.
53. Tarailo-Graovac, M., Drogemoller, B.I., Wasserman, W.W., Ross, C.J., van den Ouweland, A.M., Darin, N., Kollberg, G., van Karnebeek, C.D., and Blomqvist, M. (2017). Identification of a large intronic transposal insertion in SLC17A5 causing sialic acid storage disease. Orphanet J. Rare Dis. *12*, 28. 10.1186/s13023-017-0584-6.
54. Maroilley, T., Wright, N.A.M., Diao, C., MacLaren, L., Pfeffer, G., Sarna, J.R., Billie Au, P.Y., and Tarailo-Graovac, M. (2022). Case Report: Biallelic Loss of Function ATM due to Pathogenic Synonymous and Novel Deep Intronic Variant c.1803-270T > G Identified by Genome Sequencing in a Child With Ataxia-Telangiectasia. Frontiers in genetics *13*, 815210. 10.3389/fgene.2022.815210.
55. Matthews, A.M., Tarailo-Graovac, M., Price, E.M., Blydt-Hansen, I., Ghani, A., Drögemöller, B.I., Robinson, W.P., Ross, C.J., Wasserman, W.W., Siden, H., and van Karnebeek, C.D. (2017). A de novo mosaic mutation in SPAST with two novel alternative alleles and chromosomal copy number variant in a boy with spastic paraplegia and autism spectrum disorder. Eur. J. Med. Genet. *60*, 548-552. 10.1016/j.ejmg.2017.07.015.
56. Fong, H.K., Hurley, J.B., Hopkins, R.S., Miake-Lye, R., Johnson, M.S., Doolittle, R.F., and Simon, M.I. (1986). Repetitive segmental structure of the transducin beta subunit: homology with the CDC4 gene and identification of related mRNAs. Proc. Natl. Acad. Sci. U. S. A. *83*, 2162-2166. 10.1073/pnas.83.7.2162.
57. Wu, X.H., Chen, R.C., Gao, Y., and Wu, Y.D. (2010). The effect of Asp-His-Ser/Thr-Trp tetrad on the thermostability of WD40-repeat proteins. Biochemistry *49*, 10237-10245. 10.1021/bi101321y.
58. Rouault, T.A. (2012). Biogenesis of iron-sulfur clusters in mammalian cells: new insights and relevance to human disease. Dis. Model. Mech. *5*, 155-164. 10.1242/dmm.009019.
59. Srinivasan, V., Netz, D.J., Webert, H., Mascarenhas, J., Pierik, A.J., Michel, H., and Lill, R. (2007). Structure of the yeast WD40 domain protein Cia1, a component acting late in iron-sulfur protein biogenesis. Structure *15*, 1246-1257. 10.1016/j.str.2007.08.009.
60. Kim, K.S., Maio, N., Singh, A., and Rouault, T.A. (2018). Cytosolic HSC20 integrates de novo iron-sulfur cluster biogenesis with the CIAO1-mediated transfer to recipients. Hum. Mol. Genet. *27*, 837-852. 10.1093/hmg/ddy004.
61. Seki, M., Takeda, Y., Iwai, K., and Tanaka, K. (2013). IOP1 protein is an external component of the human cytosolic iron-sulfur cluster assembly (CIA) machinery and functions in the MMS19 protein-dependent CIA pathway. J. Biol. Chem. *288*, 16680-16689. 10.1074/jbc.M112.416602.

62. Balk, J., Pierik, A.J., Netz, D.J., Mühlenhoff, U., and Lill, R. (2004). The hydrogenase-like Nar1p is essential for maturation of cytosolic and nuclear iron-sulphur proteins. EMBO J. *23*, 2105-2115. 10.1038/sj.emboj.7600216.
63. Maione, V., Grifagni, D., Torricella, F., Cantini, F., and Banci, L. (2020). CIAO3 protein forms a stable ternary complex with two key players of the human cytosolic iron-sulfur cluster assembly machinery. J. Biol. Inorg. Chem. *25*, 501-508. 10.1007/s00775-020-01778-z.
64. Xu, C., and Min, J. (2011). Structure and function of WD40 domain proteins. Protein & cell *2*, 202-214. 10.1007/s13238-011-1018-1.
65. Kassube, S.A., and Thomä, N.H. (2020). Structural insights into Fe-S protein biogenesis by the CIA targeting complex. Nat. Struct. Mol. Biol. *27*, 735-742. 10.1038/s41594-020-0454-0.
66. Paul, V.D., and Lill, R. (2015). Biogenesis of cytosolic and nuclear iron-sulfur proteins and their role in genome stability. Biochim Biophys Acta *1853*, 1528-1539. 10.1016/j.bbamcr.2014.12.018.
67. Stehling, O., Vashisht, A.A., Mascarenhas, J., Jonsson, Z.O., Sharma, T., Netz, D.J., Pierik, A.J., Wohlschlegel, J.A., and Lill, R. (2012). MMS19 assembles iron-sulfur proteins required for DNA metabolism and genomic integrity. Science *337*, 195-199. 10.1126/science.1219723.
68. Petronek, M.S., and Allen, B.G. (2023). Maintenance of genome integrity by the late-acting cytoplasmic iron-sulfur assembly (CIA) complex. Frontiers in genetics *14*, 1152398. 10.3389/fgene.2023.1152398.
69. Ferlazzo, M., Berthel, E., Granzotto, A., Devic, C., Sonzogni, L., Bachelet, J.T., Pereira, S., Bourguignon, M., Sarasin, A., Mezzina, M., and Foray, N. (2020). Some mutations in the xeroderma pigmentosum D gene may lead to moderate but significant radiosensitivity associated with a delayed radiation-induced ATM nuclear localization. Int J Radiat Biol *96*, 394-410. 10.1080/09553002.2020.1694189.
70. Gari, K., León Ortiz, A.M., Borel, V., Flynn, H., Skehel, J.M., and Boulton, S.J. (2012). MMS19 links cytoplasmic iron-sulfur cluster assembly to DNA metabolism. Science *337*, 243-245. 10.1126/science.1219664.
71. White, R.J., Collins, J.E., Sealy, I.M., Wali, N., Dooley, C.M., Digby, Z., Stemple, D.L., Murphy, D.N., Billis, K., Hourlier, T., et al. (2017). A high-resolution mRNA expression time course of embryonic development in zebrafish. Elife *6*. 10.7554/eLife.30860.
72. Plaster, N., Sonntag, C., Busse, C.E., and Hammerschmidt, M. (2006). p53 deficiency rescues apoptosis and differentiation of multiple cell types in zebrafish flathead mutants deficient for zygotic DNA polymerase delta1. Cell Death Differ *13*, 223-235. 10.1038/sj.cdd.4401747.
73. Ferreira, C.R., Rahman, S., Keller, M., and Zschocke, J. (2021). An international classification of inherited metabolic disorders (ICIMD). J. Inherit. Metab. Dis. *44*, 164-177. 10.1002/jimd.12348.
74. Lee, J.J.Y., Wasserman, W.W., Hoffmann, G.F., van Karnebeek, C.D.M., and Blau, N. (2018). Knowledge base and mini-expert platform for the diagnosis of inborn errors of metabolism. Genet. Med. *20*, 151-158. 10.1038/gim.2017.108.
